# Imidazoles as Potential Anticancer Agents: An Update on Recent Studies

**DOI:** 10.3390/molecules26144213

**Published:** 2021-07-11

**Authors:** Pankaj Sharma, Chris LaRosa, Janet Antwi, Rajgopal Govindarajan, Karl A. Werbovetz

**Affiliations:** 1 Division of Medicinal Chemistry and Pharmacognosy, College of Pharmacy, The Ohio State University, Columbus, OH 43210, USA; sharma.1032@osu.edu (P.S.); larosa.18@osu.edu (C.L.); 2 Division of Mathematics, Computer & Natural Sciences Division, Ohio Dominican University, Columbus, OH 43219, USA; antwij@ohiodominican.edu; 3 Division of Pharmaceutics and Pharmacology, College of Pharmacy, The Ohio State University, Columbus, OH 43210, USA; govindarajan.21@osu.edu

**Keywords:** imidazole, benzimidazole, purine, anticancer, antimicrotubule, kinase inhibitor

## Abstract

Nitrogen-containing heterocyclic rings are common structural components of marketed drugs. Among these heterocycles, imidazole/fused imidazole rings are present in a wide range of bioactive compounds. The unique properties of such structures, including high polarity and the ability to participate in hydrogen bonding and coordination chemistry, allow them to interact with a wide range of biomolecules, and imidazole-/fused imidazole-containing compounds are reported to have a broad spectrum of biological activities. This review summarizes recent reports of imidazole/fused imidazole derivatives as anticancer agents appearing in the peer-reviewed literature from 2018 through 2020. Such molecules have been shown to modulate various targets, including microtubules, tyrosine and serine-threonine kinases, histone deacetylases, p53-Murine Double Minute 2 (MDM2) protein, poly (ADP-ribose) polymerase (PARP), G-quadraplexes, and other targets. Imidazole-containing compounds that display anticancer activity by unknown/undefined mechanisms are also described, as well as key features of structure-activity relationships. This review is intended to provide an overview of recent advances in imidazole-based anticancer drug discovery and development, as well as inspire the design and synthesis of new anticancer molecules.

## 1. Introduction

According to the 2020 World Cancer Report, slightly over 18 million new cases of cancer and nearly 10 million cancer-related deaths occurred across the globe in 2018 [1]. Cancer is also the first or second leading cause of premature death in people of ages 30–69 in most countries worldwide. It is characterized by uncontrolled cell growth which may spread to other parts of the body (known as metastasis) and invade other tissues. Although prevention efforts are critical to limit cancer incidence, the treatment of cancer often involves pharmacologic intervention. Cytotoxic chemotherapeutic agents continue to play an important role in cancer pharmacotherapy, but discovery efforts have increasingly turned to targeted therapies (drugs interfering with processes unique to the proliferation and spread of cancer cells) and immunotherapy (boosting the immune system or changing how the immune system works) as effective and less toxic forms of pharmacotherapy for cancer [2,3]. While numerous anticancer drugs are available, many forms remain difficult to cure, resulting in the mortality rates mentioned above. The toxicity, rapid development of resistance, and limited efficacy associated with currently available anticancer agents highlight the urgency to discover new compounds that can overcome the limitations of existing drugs [4].

**Figure 1:**
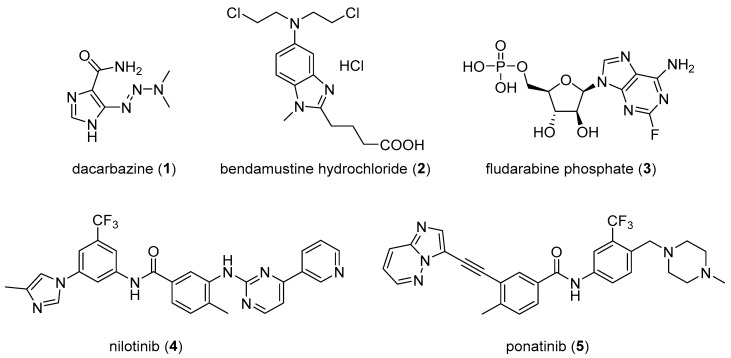
Selected anticancer drugs containing imidazole and fused imidazole moieties.

An analysis of U.S. FDA approved drugs revealed that 59% of these small-molecule agents include nitrogen-containing heterocycles [5]. Imidazole is among the top ten and fused imidazoles (benzimidazole and imidazopyrimidine (purine)) are among the top 25 most frequently appearing nitrogen heterocycles in such small molecule drugs [5]. These ring systems are key components of structural scaffolds occurring in modern medicinal chemistry, thus forming critical building blocks for new drug design. Compounds containing an imidazole ring display a wide range of pharmacological activities including anticancer [6], antibacterial [7], antiviral [8], antiepileptic [9], antitubercular [10], and antifungal activities [11]. A range of anticancer drugs, such as dacarbazine (**1**), bendamustine hydrochloride (**2**), fludarabine phosphate (**3**), nilotinib (**4**), and ponatinib (**5**), contain imidazole and fused imidazole as structural components (see Figure 1). Many biological targets have been investigated and identified for imidazole and fused imidazole derivatives through which they are thought to exhibit their anticancer activities. These proposed targets include tubulin/microtubules, a range of kinases, histone deacetylases and other proteins that regulate gene expression, and additional targets as detailed in this review article.

Imidazole, a five-membered heterocycle, is highly polar (experimental logP close to zero) [12] due to the presence of two nitrogen atoms. This ring system can serve as an acid or a base; imidazole can act as a hydrogen bond donor if it is not substituted at N1 and is also capable of coordinating metals [13,14]. In addition to hydrogen bonding and coordination, imidazole can participate in van der Waals interactions, π–π stacking, cation–π interactions, and other interactions [15]. The versatility of imidazole in terms of its molecular interactive properties is reflected by its presence in the amino acid histidine and in the purine bases, thus making imidazole ubiquitous in biochemistry. The first synthesis of imidazole, in which glyoxal, ammonia, and formaldehyde reacted to prepare the ring in low yield, was reported by Debus in 1858 [16]. The Radiszewski synthesis [17] is an extension of the Debus synthesis in which 1,2-dicarbonyl compounds, aldehydes, and amines react to form substituted imidazoles, a method that is still employed for the synthesis of various substituted imidazoles. The Wallach synthesis [18] and the Marckwald synthesis [19] are other named reactions employed for the synthesis of imidazoles, but numerous approaches have been reported for the preparation of this ring system. Reviews by Sharma et al. [20], Vessaly et al. [21], Hossain and Nanda [22], Soni et al. [23], and Shabalin and Camp [24] have described various methods used for the synthesis of imidazoles. The review by Alaqueel et al. reported that benzimidazole preparation is generally through the reaction of *o*-phenylenediamine with different benzaldehydes [25], while the purine nucleus is often prepared by the Traube synthesis [26] or its modern variations [27]. Purine ring systems may also be preformed and modified at electrophilic sites in the process of analog preparation. Imidazole-containing heterocycles are also known as privileged scaffolds due to their wide range of biological activities, prompting the publication of comprehensive review articles detailing such activities [15,28,29]. In 2017, Ali et al. summarized the potential of imidazoles as anticancer agents [6]. Since this review by Ali et al., numerous studies detailing the anticancer potential of such compounds have appeared. Therefore, the present review addresses recent developments concerning imidazole-based compounds displaying anticancer activity published from 2018–2020. We apologize for any unintentional omission of relevant published research articles which we have missed in preparing this manuscript. Nevertheless, this review summarizes approximately 120 original studies. The brief accounts of these publications included below recap the activity of imidazole-containing compounds against different cancer cell lines, offer a synopsis of structure-activity relationships in many cases, and provide highlights concerning in vivo efficacy. A strong focus has also been placed on mechanism of action studies. To this end, the candidate molecules described in this review are grouped according to their proposed molecular target, when possible, with an additional section containing molecules with unknown/uncharacterized targets. Thus, another goal of this review is to provide a representative sampling of the molecular targets that are being explored in current anticancer drug design. We hope that this review of the recent literature on imidazole- and fused imidazole-based compounds as anticancer candidates will be useful and motivating to researchers worldwide to aid the design and synthesis of compounds with improved potency along with high selectivity for different types of cancers.

## 2. Imidazoles as Tubulin Polymerization Inhibitors

Microtubules are the cylindrical components of the cytoskeleton (composed of α- and β-tubulin heterodimers) which are critical players in various cellular functions including the maintenance of cell shape, cell signaling, vesicular transport, and cell division. Because they are the primary component of the mitotic spindle, microtubules play an important role in the proliferation of cancer cells, thus making microtubules one of the most attractive targets for anticancer drugs (reviewed in reference [30]). Microtubule targeting agents can disrupt the formation of mitotic spindles at the metaphase/anaphase transition, altering tubulin assembly kinetics and causing cell-cycle arrest and apoptotic cell death. Although tubulin targeting compounds, such as taxanes [31], vinca alkaloids [32], and some newer agents, such as ixabepilone [33], are used for the treatment of cancer, multidrug resistance and poor bioavailability associated with these drugs pose challenges, as well as provide motivation, for researchers to identify new microtubule-targeted agents with high efficacy, an acceptable side effect profile, and good bioavailability. 

**Figure 2:**
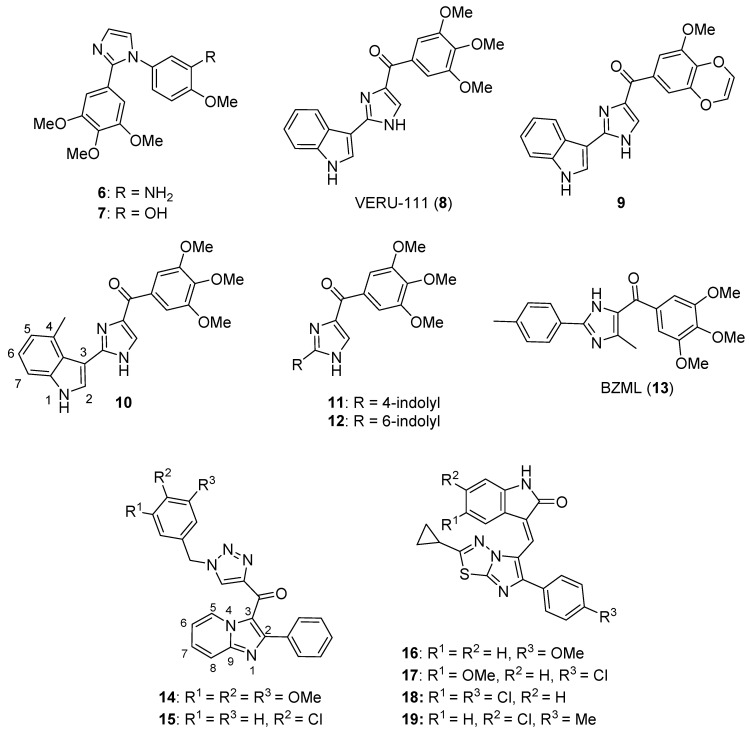
Imidazole-containing tubulin assembly inhibitors reported in the literature (compounds **6**–**19**).

1-Substituted-2-aryl imidazoles were synthesized by Li et al. as potential tubulin-targeted anticancer agents [34]. The target compounds were tested on MDA-MB-468, MDA-MB-231, T47D, HCT-15, HT29, and HeLa cancer cell lines along with a normal human umbilical vein endothelial cell line (HUVEC). Many of the compounds possessing an aromatic ring on the imidazole nitrogen atom displayed potent antiproliferative activities with IC_50_ values in the 80–1000 nM range. Among these, compound **6** (see Figure 2 for structures of compounds **6**–**19**) showed the highest potency, with IC_50_ values from 80–200 nM against HCT-15, HT29, HeLa, and MDA-MB-468 cells, while compound **7** exhibited good potency against HeLa and HCT-15 cells (IC_50_ values of 100 and 200 nM, respectively). In terms of the SAR, the placement of an aliphatic group on the imidazole nitrogen and the replacement of imidazole with an amide or ester group led to a loss of activity. Compound **6** was a better inhibitor of porcine brain tubulin polymerization (IC_50_ = 0.4 µM) than either colchicine (IC_50_ = 7.5 µM) or combretastatin A-4 (IC_50_ = 1.1 µM). At its IC_50_ concentration, compound **6** caused arrest of MDA-MB-468 cells in the G_2_/M phase of the cell cycle. In an MDA-MB-468 breast cancer xenograft model performed in nude mice, i.p. administration of compound **6** (60 mg/kg every other day for 21 days) suppressed tumor growth by 77% compared to control, without causing obvious weight loss in the animals.

Wang et al. designed novel anticancer compounds based on the clinical drug candidate VERU-111 (**8**) where structural modification of the 3,4,5-trimethoxyphenyl group in this compound led to improved anticancer activity [35]. The authors presented an elegant synthetic approach where 3-methoxybenzo[4,5]-dioxene (a ring system found in the most potent compound) was synthesized using a second-generation Grubbs catalyst via ring closing metathesis. The antiproliferative effects of these compounds were evaluated on A375, M14, and RPMI7951 human melanoma cell lines. Compound **9** (containing a 3-methoxybenzo[4,5]-dioxene ring system) was the most active, with IC_50_ values of 1.1 nM, 1.2 nM and 3.3 nM on A375, M14, and RPMI7951 cell lines, respectively. When the trimethoxyphenyl group present in compound **8** was replaced with 4-methoxybenzo[*d*][1,3]dioxole, 5-methoxy-2,3-dihydrobenzo[*b*][1,4]dioxene, or 6-methoxy-3,4-dihydro-2*H*-benzo[*b*][1,4]dioxepine, the activity was greatest with the dioxole ring-containing compound and decreased with increasing ring size. Incubation of compound **9** with bovine brain tubulin at a concentration of 10 μM resulted in nearly complete inhibition of tubulin assembly. The X-ray crystal structure of compound **9** bound to the tubulin/stathmin-like domain of RB-3/tubulin tyrosine ligase complex confirmed that **9** binds to the colchicine site on β-tubulin. When given at a dose of 30 mg/kg every other day for 15 days by i.p. injection, compound **9** inhibited tumor growth by 73.9% in an A375 murine melanoma xenograft model without significant weight loss.

Additional work regarding **8** (also known as ABI-231) conducted by Wang et al. reported structure-activity relationship (SAR) studies around the 3-indole moiety of this molecule [36]. Target compounds were screened against the A375, WM164, and M14 human melanoma cell lines. Among these compounds, **10**–**12** displayed the greatest potency, with IC_50_ values ranging from 1.6–8.0 nM against the cancer cell lines tested. In terms of the SAR for 3-indolyl compounds, derivatives bearing bulky groups at the other positions on the indole ring system displayed reduced activity, while substitution with a methyl group at the 4-position of indole (compound **10**) resulted in a marked increase in antiproliferative activity. When the point of attachment to the indole ring system was changed, compounds where trimethoxybenzoyl imidazole was placed at the 4-, 5-, or 6-position of indole displayed superior antiproliferative activity compared to the compound harboring this substitution at the C7 position of indole. Compounds **10**–**12** were tested against the NCI 60 cell line panel and displayed low nanomolar IC_50_ values against most of these cell lines. At a concentration of 10 μM, compounds **10** and **11** completely inhibited the polymerization of purified bovine brain tubulin in vitro. Further, X-ray crystallographic studies verified that the lead compound **8**, as well as compounds **10** and **11**, bind at the colchicine site on tubulin. Intraperitoneal injection of compound **11** (30 mg/kg/day on alternate days for 20 days) resulted in 90.6% tumor growth inhibition in an A375 melanoma xenograft model in nude mice. In a lung metastasis model conducted in C57BL mice, animals treated with 30 mg/kg compound **11** for two weeks (five days per week) by intraperitoneal injection showed an 80.9% decrease in metastasis. Moreover, in a taxane-resistant PC-3 (PC-3/TxR) mouse model, administration of compound **11** (30 mg/kg) resulted in an 83.8% reduction in tumor growth and 62.8% decrease in the average tumor weight compared to controls.

Bai et al. reported on 5-(3,4,5-trimethoxybenzoyl)-4-methyl-2-(*p*-tolyl) imidazole (BZML, **13**), a compound with potent activity against colorectal cancer cell lines that inhibits tubulin polymerization and causes DNA damage [37]. Compound **13** displayed potent IC_50_ values of 27.42, 23.12, and 33.14 nM against SW480, HCT116, and Caco-2 cells, respectively, while the Caco-2 cell line was insensitive to both paclitaxel and doxorubicin (IC_50_ values > 1800 nM). When tested at a concentration of 60 nM, compound **13** disrupted microtubules in these cell lines as assessed by immunofluorescence microscopy and increased the number of γ-H2AX foci in the cell lines mentioned above, indicating the induction of DNA damage. Moreover, this compound decreased P-glycoprotein (P-gp) expression and enhanced the activity of both doxorubicin and paclitaxel in the Caco-2 cell line at a concentration of 60 nM.

A series of new imidazopyridine-triazole conjugates were reported as potential tubulin polymerization inhibitors and were screened against A549, DU-145, HCT116, and MDA-MB 231 cancer cell lines [38]. Compounds **14** and **15** displayed IC_50_ values of 0.51 and 0.63 µM against the A549 cell line, respectively, and exhibited the greatest potency against the four cancer cell lines overall. At a concentration of 3 µM, compounds **14** and **15** inhibited tubulin polymerization in a fluorescence-based assay by 59% and 56%, respectively, while the standard compound nocodazole displayed 55% inhibition. At 1 µM concentrations, compounds **14** and **15** also caused a dramatic increase in the percentage of A549 cells in the G_2_/M phase. Molecules bearing an unsubstituted phenyl ring at the C2 position of the imidazopyridine system generally displayed the greatest potency.

Narasimha Rao et al. synthesized a library of imidazothiadiazole-oxindole conjugates and evaluated their antimicrotubule activity, as well as their antiproliferative activity, against HeLa, MCF-7, and MIAPaCa-2 cancer cell lines and HEK-293 human embryonic kidney cells [39]. Compounds **16**–**19** displayed the greatest activity overall, exhibiting submicromolar or low micromolar GI_50_ values against all three cell lines. When compound **18** was screened against the NCI 60 cancer cell line panel, GI_50_ values below 5.0 μM were observed against most of these cell lines, with the exception of leukemia and colon cancer cells. Compounds **16**, **17**, and **18** were inhibitors of bovine brain tubulin polymerization, displaying IC_50_ values of 5.6, 2.8, and 4.6 μM, respectively. Accumulation of HeLa cells in the G_2_/M phase was observed upon incubation with 5 µM concentrations of compounds **16**–**18**, while these compounds activated caspase-3 in HeLa cells at 2 µM concentrations. While the SAR was complex, inclusion of a nitro group either on the oxindole system or on the phenyl ring generally decreased the anticancer activity.

**Figure 3:**
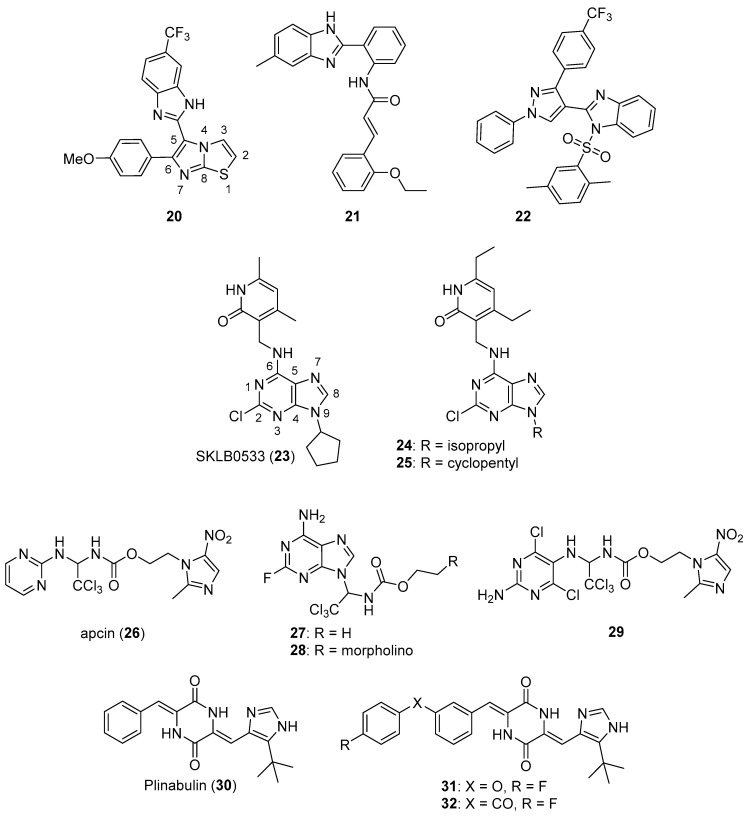
Additional imidazole-containing tubulin assembly inhibitors reported in the literature (compounds **20**–**32**).

Baig et al. synthesized imidazothiazole-benzimidazole derivatives as potential tubulin polymerization inhibitors and tested their cytotoxicity against HeLa, A549, MCF-7, and DU-145 cancer cell lines [40]. Compound **20** (see Figure 3 for structures of compounds **20**–**32**) exhibited an IC_50_ value of 1.09 µM against the A549 cancer cell line. Analogs bearing a 4-methoxy substitution on the phenyl ring at C6 of the imidazothiazole ring system generally displayed high activity against the A549 cell line, although the substitutions on the benzimidazole ring system at C5 also influenced activity. Compound **20** inhibited porcine tubulin polymerization in a fluorescence-based assay with an IC_50_ value of 1.68 µM, while the IC_50_ value for the standard compound nocodazole was 1.99 µM in this assay. At a concentration of 2 µM, compound **20** caused the accumulation of A549 cells in the G_2_/M phase of the cell cycle.

Donthiboina et al. synthesized benzimidazole-cinnamide derivatives as potential tubulin polymerization inhibitors and evaluated their activity against A549, MDA-MB-231, B16F10, BT474, and 4T1 cell lines [41]. Among the synthesized compounds, **21** displayed IC_50_ values in the range between 0.29 to 1.48 μM against the cancer cell lines tested, exhibiting the greatest potency against A549 cells. Compound **21** also displayed an IC_50_ value of 1.58 μM against a normal rat kidney epithelial cell line (NRK-52E cells). This compound inhibited porcine tubulin polymerization with an IC_50_ value of 4.64 μM, while incubation of A549 cells with 0.5 μM concentrations of this compound caused an increase in the percentage of cells in the G_2_/M phase of the cell cycle. In general, compounds possessing electron donating substituents on the phenyl portion of the benzimidazole group were more active than compounds bearing electron withdrawing substituents.

Wang et al. synthesized a series of benzimidazole sulfonamides containing a substituted pyrazole ring at the C2 position [42]. These derivatives displayed IC_50_ values in the range between 0.15–7.26 μM against A549, HeLa, HepG2, and MCF-7 cancer cell lines. Compound **22** was the most potent derivative, displaying IC_50_ values of 0.15, 0.21, 0.33, and 0.17 μM against A549, HeLa, HepG2, and MCF-7 cells, respectively. This compound also showed low toxicity to murine primary hepatocytes and 293T cells (CC_50_ values of 132 and 223 μM, respectively). In terms of the SAR for these derivatives against the cancer cell lines, placement of the benzene sulfonyl group on the benzimidazole nitrogen enhanced activity and the inclusion of 2,5-dimethyl, 4-methyl and 4-methoxy groups on the phenylsulfonyl ring increased potency. Compound **22** displayed an IC_50_ value of 1.52 μM against porcine brain tubulin polymerization, while colchicine and combretastatin A4 displayed IC_50_ values of 2.26 μM and 1.61 μM in the same assay, respectively. At a concentration of 5 μM, compound **22** inhibited the binding of [^3^H]colchicine (5 μM) to tubulin by 91%. Incubation with this compound at a concentration of 0.5 μM resulted in an increase in the percentage of A549 cells in the G_2_/M phase.

Zhang et al. synthesized aminomethyl dimethylpyridinones as potential anticancer agents against HepG2, HCT116, and A549 cancer cell lines and also evaluated their tubulin polymerization inhibitory activity [43]. Most of these compounds exhibited antiproliferative activity with IC_50_ values of <10 μM against these cell lines. Among these compounds, SKLB0533 (**23**) was the most potent, displaying IC_50_ values of 0.08, 0.06, and 0.10 μM against HepG2, HCT116, and A549 cells compared to the standard compound myoseverin (IC_50_ values of 3.06, 7.82, and >10 μM, respectively). In general, substitution with 2-propyl, 3-pentyl, or cyclopentyl groups at N9 resulted in increased antiproliferative activity compared to N9-tetrahydropyranyl derivatives. Compound **23** inhibited the assembly of porcine tubulin in vitro in a dose-dependent manner and caused the dose-dependent accumulation of HCT116 and SW620 cells in the G_2_/M cell cycle phase (the highest concentration tested was 75 nM in cell cycle analysis experiments). Moreover, compound **23** displayed no activity against a panel of 420 kinases when tested at a concentration of 1 µM, suggesting its specific tubulin inhibitory activity. This compound displayed high activity against a panel of other colorectal cancer cell lines (SW620, IC_50_ = 60 nM; CT26, IC_50_ = 45.5 nM; SW480, IC_50_ = 64.5 nM; DLD-1, IC_50_ = 135.5 nM; HCT-15, IC_50_ = 61.0 nM, and HT29, IC_50_ = 71.0 nM). In a murine xenograft model employing HCT116 cells, oral administration of compound **23** at 40 or 80 mg/kg once daily for 28 days resulted in tumor growth inhibition (TGI) of 39.9% and 71.6% compared to the control group, respectively, while the TGI for the group receiving oral capecitabine at 540 mg/kg once daily for 28 days was 61.6%.

In a further extension of the work described above, Hu et al. synthesized a series of derivatives based on compound **23** [44]. These target compounds displayed high potency against CT26, SW620, and HCT116 colorectal cancer cell lines. Compound **24** displayed IC_50_ values of 0.022, 0.009, and 0.058 μM against the cell lines mentioned above, respectively, while SKLB0565 (**25**) exhibited IC_50_ values of 0.057, 0.040, and 0.021 μM, respectively, against these cell lines. Compound **25** displayed concentration-dependent inhibition of porcine tubulin assembly and also caused concentration-dependent accumulation of HCT116 and SW620 cells in the G_2_/M cell cycle phase at 25–100 nM concentrations. In addition, this compound displayed activity against other colorectal cancer cell lines (SW480, IC_50_ = 0.081 μM; SW48, IC_50_ = 0.015 μM; DLD-1, IC_50_ = 0.074 μM; HCT-15, IC_50_ = 0.029 μM; and HT29, IC_50_ = 0.029 μM). At concentrations of 50 nM and 100 nM, compound **25** displayed in vitro anti-vascular activity in HUVECs in wound healing and capillary tube formation assays.

Huang et al. designed new purine and pyrimidine molecules based on the structural modification of apcin (**26**), a substituted 2-aminopyrimidine compound [45]. Compound **26** is a specific inhibitor of Cdc20, a protein which activates the key E3 ubiquitin ligase APC/C that controls cell cycle progression; Cdc20 is overexpressed in several cancers (see Reference [46] for a review of Cdc20 as an anticancer target). Of the compounds tested, **27** and **28** generally displayed the greatest potency against cancer cell lines, with IC_50_ values in the range between 0.2–1.8 μM against MCF-7, A375, A549, OVCAR-3, Caov-3, HepG2, and HeLa cancer cell lines. By comparison, the IC_50_ values of **26** were greater than 193 μM against these cell lines. In terms of the SAR for the purine-containing compounds against cancer cell lines, substitution with fluorine rather than chlorine at C2 of the purine ring system resulted in greater potency. Compound **29** displayed a K_d_ = 49 μM for Cdc20 as measured by surface plasmon resonance (SPR), while the binding of **26** and **28** to Cdc20 was weaker (K_d_ values of 123 μM and 119 μM, respectively). Compounds **29** (30 μM) and **28** (0.3 μM) both blocked the exit of HepG2 cells from mitosis as assessed by the increased levels of phosphohistone H3, a marker of mitosis. Given the potency of **28** compared to **29**, the authors hypothesized that the former compound could possess an additional mechanism of action. Compound **28** was shown to dose-dependently (3 to 300 μM) inhibit the assembly of porcine tubulin in vitro, consistent with this molecule acting as an antimicrotubule agent.

Plinabulin (**30**), a microtubule targeting anticancer drug candidate with multiple proposed mechanisms of action, has undergone multiple clinical trials [47]. Ding et al. designed and synthesized new compounds based on a co-crystal structure of plinabulin bound to tubulin [48]. Compound **31** displayed IC_50_ values against NCI-H460, HepG2, HCT116, MCF-7, and HeLa cells of 4.0, 3.0, 3.8, 9.0, and 8.4 nM, while plinabulin displayed IC_50_ values of 26.2, 4.8, 6.0, 29.8, and 9.0 nM against these cancer cell lines. At concentrations of 5 µM, compound **31** inhibited tubulin polymerization by 39.7%, while plinabulin and compound **32** (which exhibited an IC_50_ value of 3.8 nM against NCI-H460 cells) displayed 13.5% and 47.3% inhibition, respectively. Compounds **31** and **32** also disrupted microtubules in NCI-H460 cells at a concentration of 5 nM as assessed by immunofluorescence.

## 3. Imidazoles as Kinase Inhibitors

Protein kinases play an important role in the progression of various cellular processes, such as growth, differentiation, and apoptosis. These enzymes are classified as serine/threonine or tyrosine kinases based on the identity of the phosphorylated amino acid. While expression of these kinases is strictly controlled in normal cells, overexpression of serine/threonine and tyrosine kinases has been reported in a variety of cancers. Thus, the inhibition of these kinases with small molecules can provide a strategy for cancer treatment. Tyrosine kinases discussed below include vascular endothelial growth factor receptor (VEGFR), Src family kinase (SFK), and activin receptor-like kinase 5 (ALK5). Serine-threonine kinases mentioned in this section include checkpoint kinases 1 and 2 (Chk1 and Chk2), rapidly accelerated fibrosarcoma (RAF) kinase, cyclin-dependent kinase (CDK), Polo like kinase 1 (PLK1), Aurora kinase (AURK), and AKT. Phosphatidylinositol-3-kinase (PI3K) is a lipid kinase that transforms phosphatidylinositol (4,5)-bisphosphate into the second messenger phosphatidylinositol (3,4,5)-trisphosphate. A variety of drugs targeting receptor tyrosine kinases, non-receptor tyrosine kinases and serine-threonine kinases have been developed (reviewed in Reference [49]), and the discovery of selective kinase inhibitors continues to be the focus of many medicinal chemists.

### 3.1. Tyrosine Kinase Inhibitors

#### 3.1.1. Vascular Endothelial Growth Factor Receptor (VEGFR) Inhibitors

The formation of new blood vessels (angiogenesis) is required for cell and tissue growth. Cancer growth and metastasis depend directly on tumor angiogenesis and vascularization. Vascular endothelial growth factor (VEGF) is a signaling protein that plays an important role in cellular proliferation, survival, and migration during the development of new vasculature, and most human cancers display up-regulated expression of VEGF mRNA. VEGF mediates its biological effects through binding to VEGF receptors (VEGFRs), tyrosine kinases that dimerize and become autophosphorylated upon binding VEGF, triggering a range of downstream effects (for recent reviews of VEGF and VEGF signaling, see references [50,51]). Various therapeutic agents, such as monoclonal antibodies (bevacizumab) and small molecules (sunitinib and sorafenib), have been approved as VEGFR inhibitors, but side effects, such as hypertension, are associated with the small molecule agents [52]. New VEGFR inhibitors with an improved therapeutic window are, thus, being sought.

**Figure 4:**
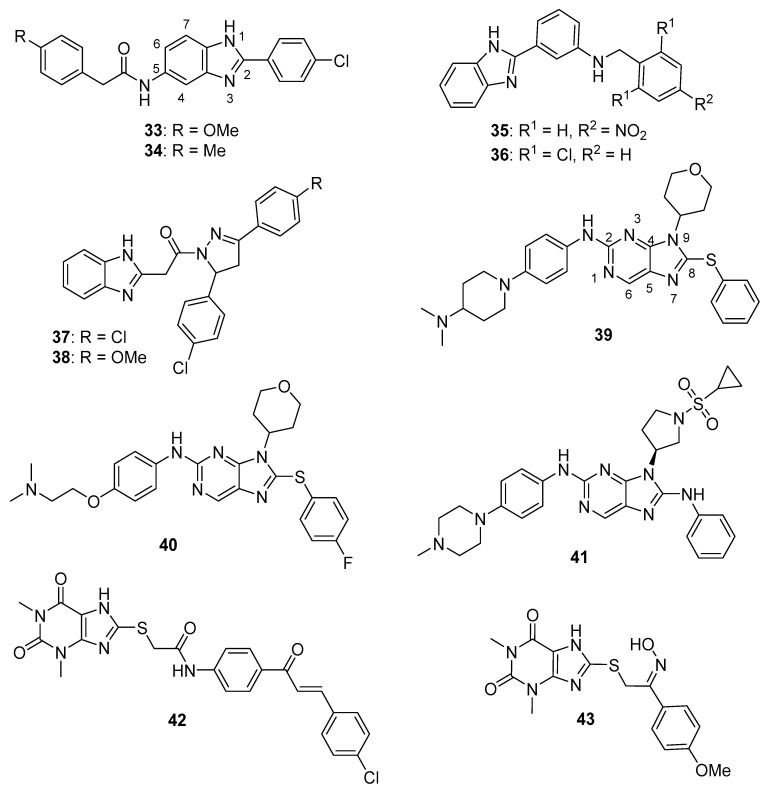
Imidazole-containing tyrosine kinase inhibitors targeting VEGFR-2 and EGFR reported in the literature (compounds **33**–**43**).

Yuan et al. reported several compounds possessing a benzoxazole/benzimidazole scaffold displaying potent in vitro antiproliferative activity against HUVEC, HepG2, MDA-MB-231, and A549 cell lines through the inhibition of VEGFR-2 kinase [53]. These compounds showed potent activity against VEGFR-2 expressing cell lines (HepG2 and HUVEC), but they were less active on EGFR expressing cell lines (MDA-MB-231 and A549). Compounds **33** and **34** (see Figure 4 for structures of compounds **33**–**43**) displayed activity against HUVECs (IC_50_ values of 1.47 and 3.21 µM) and HepG2 cells (IC_50_ values of 2.57 and 5.56 µM). The compounds possessing a benzimidazole scaffold were generally more active against these cell lines than benzoxazole-containing compounds. Compound **33** displayed an IC_50_ value of 51.4 nM against VEGFR-2, while inhibiting EGFR by 58.3%, when tested at a concentration of 1 µM. Compound **33** displayed little or no activity on a panel of five other kinases (PGDFR-α and β, FLT1, FLT4, and MET). Moreover, compound **33** displayed 56.4% inhibition against LO2 (normal human liver) cells at a concentration of 10 µM. Dose-dependent anti-angiogenic activity was observed with compound **33** using a chick chorioallantoic membrane assay (79% at 10 nM/eggs). Western blotting studies showed that compound **33** inhibited VEGFR-2 phosphorylation in HUVECs at concentrations of 2 µM and 4 µM.

Mostafa et al. synthesized 2-phenyl benzimidazole derivatives as potential anticancer agents that were evaluated against the MCF-7 cell line and also against VEGFR-2 [54]. Of the target compounds, **35** and **36** displayed IC_50_ values of 3.37 and 6.30 μM against MCF-7 cells, respectively, while the IC_50_ value of the standard drug doxorubicin was 4.17 µM. When these compounds were tested against three normal human cell lines (breast epithelial (MCF-10F), skin fibroblast (BJ), and lung fibroblast (MRC-5) cells), IC_50_ values ranged from 17.3–79.3 μM, while the IC_50_ value for doxorubicin against these cells ranged from 15.2–22.6 μM. In an ELISA-based VEGFR assay, these compounds displayed IC_50_ values of 6.7 and 6.9 nM, respectively, while the reference drug sorafenib displayed an IC_50_ value of 7.6 nM.

#### 3.1.2. Epidermal Growth Factor Receptor (EGFR) Inhibitors

The epidermal growth factor receptor (EGFR) is a receptor tyrosine kinase (RTK) known to play an important role in the physiology of epithelial cells by regulating several cellular processes including cell survival and differentiation. Activation of EGFR results in signaling through several pathways, including the PI3K/AKT and Ras/MAPK pathways. Mutation or overexpression of EGFR occurs in many cancers, including lung cancer and glioblastoma, and the EGFR is the target of small molecule inhibitor drugs, such as erlotinib and gefitinib, as well as EGFR-binding antibodies (see Reference [55] for a review of the roles of the EGFR in cancer). Thus, the development of new EGFR-directed agents continues to be of interest.

Akhtar et al. synthesized new benzimidazole-pyrazole derivatives as potential anticancer agents targeting EGFR [56]. Among the synthesized compounds, **37** and **38** displayed the greatest inhibition of EGFR phosphorylation in the KB cell line as assessed by ELISA, exhibiting IC_50_ values of 0.97 μM and 1.7 μM, respectively. The reference standard gefitinib exhibited an IC_50_ value of 0.011 μM in this assay. Of the four cancer cell lines examined, compounds **37** and **38** displayed the greatest antiproliferative activity against A549 cells, with IC_50_ values of 2.2 and 2.8 μM, respectively. When incubated with A549 cells at 5 and 10 µM for 24 h, compound **37** caused a dose-dependent increase in the percentage of cells in the G_2_/M cell cycle phase. No mortality was observed 24 h post-administration when a single 500 mg dose of compound **37** was given to female albino rats by oral gavage.

Hei et al. synthesized new 2,9-disubstituted purine derivatives also containing 8-phenylthio and 8-phenylsulfinyl substitutions as potential antiproliferative agents against A549, H1975, and HCC827 human lung adenocarcinoma cell lines and tested these compounds for their EGFR inhibitory activities [57]. These compounds displayed high activity against the HCC827 cell line, which harbors a deletion mutation that activates EGFR, compared to A549 cells (possessing wild type EGFR) and H1975 cells (harboring EGFR T790M and L858R mutations). Compounds **39** and **40** exhibited IC_50_ values of 29.4 nM and 47.8 nM against the HCC827 cell line and were at least two orders of magnitude less potent against A549 and H1975 cells. Among 8-phenylthio and 8-phenylsulfinyl purines with the same substitutions at positions 2 and 9, the former compounds were in general more active against HCC827 cells. In an EGFR enzymatic assay against EGFR^WT^, EGFR^L858R^, EGFR^L858R/T790M^, and EGFR^L858R/T790M/C797S^, compounds **39** and **40** displayed IC_50_ values of 1.6, 1.9, 104, 331 nM and 2.5, 1.2, 189, 114 nM, respectively. In Western blotting studies with compound **39** in the HCC827 cell line, a dose-dependent inhibition of EGFR phosphorylation was observed at concentrations of **39** ranging from 0.1–3.0 µM. When evaluated in an HCC827 xenograft model in nude Balb/c mice, compound **39** (5 mg/kg administered orally once a day for 20 days) significantly reduced tumor volume and tumor weight compared to the vehicle control group.

In a follow-up to the previous study, Lei et al. synthesized a series of 29 new purine derivatives targeting the mutant EGFR tyrosine kinases which have resulted in resistance to first, second, and third generation EGFR inhibitors [58]. The target compounds were highly active against the HCC827 cancer cell line, with IC_50_ values ranging from 0.00088–1.81 μM. Compound **41** displayed high potency against HCC827, H1975, A549, and A431 cancer cell lines, with IC_50_ values in the range between 0.0016–1.67 μM. The presence of a sulfonamide group on the substituent at the N9 position of the purine core resulted in high potency in this group of analogs, although activity against HCC827 cells was retained upon replacement of a pyrrolidinyl sulfonyl moiety with phenyl acetamide or piperidinyl amide groups. In an in vitro EGFR kinase assay performed with EGFR^L858R/T790M/C797S^, compound **41** displayed an IC_50_ value of 40 nM, while the third generation EGFR inhibitor osimertinib displayed an IC_50_ value of 110 nM. Western blotting studies with compound **41** and other analogs in HCC827, H1975, and A549 cells showed that the compounds exhibited dose-dependent inhibition of EGFR phosphorylation and AKT phosphorylation at 0.1–10-µM concentrations.

A series of 20 xanthine/chalcone hybrids were prepared and evaluated as potential EGFR-directed anticancer compounds [59]. While these molecules were not toxic to the MCF-10A human mammary gland epithelial cell line, IC_50_ values for these compounds ranged from 1.0 to 11.6 µM against A549, PANC-1, HT29, and MCF-7 cancer cells. By comparison, the standard drug doxorubicin displayed IC_50_ values ranging from 0.90 to 1.41 µM against these cell lines. In terms of the antiproliferative structure-activity relationship, the 1,3-dimethylxanthine derivatives were generally more potent than the 1-methylated xanthines. The six most active compounds in the antiproliferative assay were examined for their inhibition of EGFR kinase in a cell-free assay. IC_50_ values ranged from 0.3–1.6 µM in this assay, while the standard compound staurosporin exhibited an IC_50_ value of 0.4 µM. Compound **42**, which displayed IC_50_ values ranging from 1.3–1.8 µM against the cancer cell lines and an IC_50_ value of 0.3 µM against EGFR in vitro, also caused increased expression of the apoptotic marker caspase-3 in PANC-1 cells.

Hisham et al. synthesized substituted xanthine derivatives as EGFR-targeting anticancer agents and tested their antiproliferative activity against PANC-1, HT29, A549, and MCF-7 cancer cells and MCF-10A normal human breast epithelial cells [60]. These compounds displayed IC_50_ values in the range between 0.8–11.9 μM against the cancer cell lines, while the authors reported that all compounds were non-cytotoxic to MCF-10A cells. Compound **43** displayed IC_50_ values ≤ 1.7 μM against these cancer cell lines and was most active against MCF-7 cells (IC_50_ = 0.8 μM). In terms of the SAR against cancer cell lines, compounds containing an oxime moiety at the benzylic position generally possessed higher antiproliferative activity compared to the corresponding ketone-containing precursors. Of these target molecules, compound **43** displayed the most potent EGFR inhibitory activity (IC_50_ = 0.32 μM), while the standard drug erlotinib displayed an IC_50_ value of 0.08 μM in this assay. In PANC-1 cells, the most potent antiproliferative compounds increased the expression of caspase-3 by 4- to 8-fold with respect to controls.

**Figure 5:**
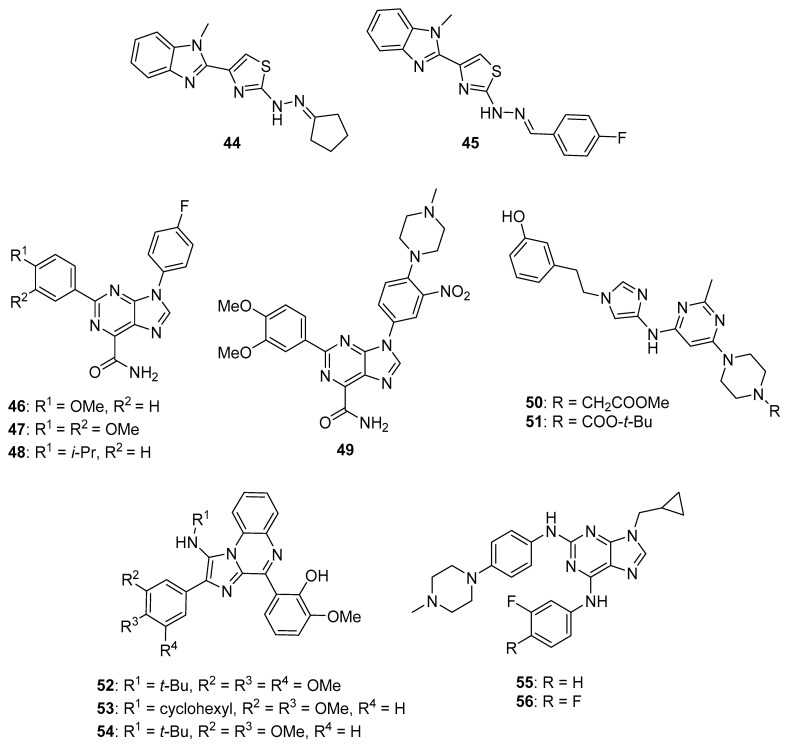
Additional imidazole-containing tyrosine kinase inhibitors and candidate inhibitors reported in the literature targeting EGFR, SFKs, and Bcr-Abl/Btk (compounds **44**–**56**).

Srour et al. synthesized thiazole-benzimidazole derivatives as potential anticancer agents directed against EGFR [61]. These compounds displayed IC_50_ values against EGFR kinase in the range between 71.7–1235 nM. Compounds that showed potent activity against EGFR were evaluated for their cytotoxicity to the MCF-7 cell line. Of these, compounds **44** and **45** (see Figure 5 for structures of compounds **44**–**56**) possessed the greatest potency against the MCF-7 cells, exhibiting IC_50_ values of 6.30 μM and 5.96 μM, respectively, while the IC_50_ of the standard drug erlotinib was 4.15 μM against this cell line. Considering that EGFR inhibitory activity did not directly correlate with activity against MCF-7 cells for these molecules, the authors speculated that other mechanisms of action may also be responsible for their anticancer activity. When tested at its IC_50_ concentration in MCF-7 cells, compound **44** caused the accumulation of these cells in the pre-G_1_ and G_2_/M cell cycle phases. At this same concentration, compound **44** increased the percentage of early and late apoptotic cells compared to the DMSO treated controls as assessed by annexin V/propidium iodide staining. Moreover, at its IC_50_ concentration, **44** increased the levels of the pro-apoptotic markers p53, Bax, and caspase-3 by 9.85-, 4.95-, and 12.28-fold in MCF-7 cells compared to control, while the anti-apoptotic marker Bcl-2 was decreased by approximately 2-fold in treated MCF-7 cells.

Kalra et al. synthesized imidazole and purine derivatives as EGFR-directed anticancer agents and tested them for antiproliferative activity against MDA-MB-231, T47D, MCF-7, HT29 and A549 cancer cell lines [62]. Purine target compounds **46** and **48** displayed IC_50_ values of 1.22 and 2.29 μM against the MDA-MB-231 cell line in an MTT assay. Purine **47** exhibited IC_50_ values in the range between 2.29–9.96 μM against these five cancer cell lines, showing the most potent activity against A549 cells. Counter-screening of compounds **46**–**48** at a concentration of 10 µM indicated that they were non-cytotoxic to normal human peripheral blood mononuclear cells (HPBMCs) and normal breast cells (HBL-100). Although selected imidazoles showed good activity against A549 cells, they exhibited greater toxicity than the purines to one or the other of the normal cell lines. Derivatization of compound **47** gave **49**, which displayed slightly greater potency than **47** (IC_50_ values for compound **49** were in the range 1.98–4.07 μM against these five cancer cell lines). Compounds **47**, **48**, and **49** displayed IC_50_ values of 617, 710, and 236 nM, respectively, against EGFR-mediated phosphorylation, while these compounds exhibited IC_50_ values > 500 nM against CDK2, CDK4, and CDK6.

#### 3.1.3. Src Family Kinase (SFK) Inhibitors

The SFKs are nonreceptor tyrosine kinases that play important roles in the regulation of cell cycle progression, apoptosis, transcription, migration, differentiation, development, and survival. Since SFKs are downstream of oncogenic drivers, such as EGFR, and are also part of pathways that regulate survival and proliferation, the inhibition of SFKs provides a good strategy for the discovery of new anticancer candidates (see Reference [63] for a review of SFKs and SFK inhibitors).

Francini et al. described the synthesis of new aminoimidazole anticancer candidates that inhibit SFKs [64]. The target compounds were screened against K562, U87 (glioblastoma multiforme (GBM)), and SH-SY5Y (neuroblastoma) cancer cell lines. Compounds **50** and **51** displayed antiproliferative activities with IC_50_ values of 8.6 and 7.8 µM against SH-SY5Y cells, 11.7 and 18.9 µM against K562 cells, and 12.6 and 13.3 µM against U87 cells, respectively. These two compounds displayed IC_50_ values against SFKs (Src, Fyn, Lyn, and Yes) in the range between 3–50 nM. In terms of the SAR for these compounds, molecules displaying *ortho* or *meta* hydroxy substitutions on the phenyl ring exhibited the highest potency against SFKs in vitro.

Rezaei et al. prepared a series of diphenylimidazo-quinoxalinamine derivatives as anticancer candidates [65]. Compounds **52, 53**, and **54** showed the greatest potencies against the K562 cell line (IC_50_ values of 9.77, 12.02, and 15.84 µM, respectively). Evaluation of compounds **52**–**54** against the tyrosine kinases c-Src, LCK (an SFK), and ABL1 (see below) revealed that only compound **54** possessed activity against ABL1 and *c*-Src with IC_50_ values of 5.25 and 3.94 µM, respectively, while this compound was not active against LCK.

#### 3.1.4. Bcr-Abl and Bruton’s Tyrosine Kinase (BTK) Inhibitors

Bcr-Abl is a tyrosine kinase that is known to play a major role in chronic myelogenous leukemia (CML), while Bruton’s tyrosine kinase (BTK) is implicated in B-cell malignancies (chronic lymphocytic leukemia and non-Hodgkin lymphomas). The expression of the Bcr-Abl fusion protein causes dysregulation of kinase activity, while BTK plays a central role in B cell receptor signaling pathways. Thus, Bcr-Abl and BTK are important targets against leukemia. Drugs, such as imatinib, nilotinib (**4**), and ponatinib (**5**), are known to target Bcr-Abl, while ibrutinib targets the BTK pathway (please see Reference [66] for a review of Bcr-Abl inhibitors and Reference [67] for a review of anticancer drugs targeting BTK). Bertrand et al. synthesized 2,6,9-trisubstituted purine derivatives as candidate dual Bcr-Abl/Btk inhibitors [68]. Most of the target compounds synthesized displayed IC_50_ values below 4 μM against Abl and Btk. Among these, compounds **55** and **56** displayed the greatest potency, both exhibiting IC_50_ values of 0.040 μM against Abl and 0.66 and 0.58 μM against Btk, respectively, while showing little activity against Cdk-2/cyclin E (IC_50_ values of 24.0 and 18.5 μM, respectively). Compounds **55** and **56** displayed IC_50_ values in the range between 0.77–8.89 μM against K562, HL-60, CEM, Ramos, MV4-11, and MCF-7 cells. Western blotting analysis revealed that the expression of the Bcr-Abl substrates pSTAT5 and pCRKL were decreased in a dose-dependent manner when compound **55** was incubated with K562 cells at concentrations ranging from 0.1–10 μM. Moreover, exposure to compound **55** at concentrations of 5, 10, and 20 μM reduced the levels of pBTK (Y223) synthesized through autophosphorylation and also lowered the levels of downstream targets pERK1/2, pPLCγ2, and pAKT in Ramos cells stimulated with Ig-M in a dose-dependent manner.

### 3.2. Serine-Threonine Kinase Inhibitors

#### 3.2.1. Activin Receptor-Like Kinase 5 (ALK5) Inhibitors

**Figure 6:**
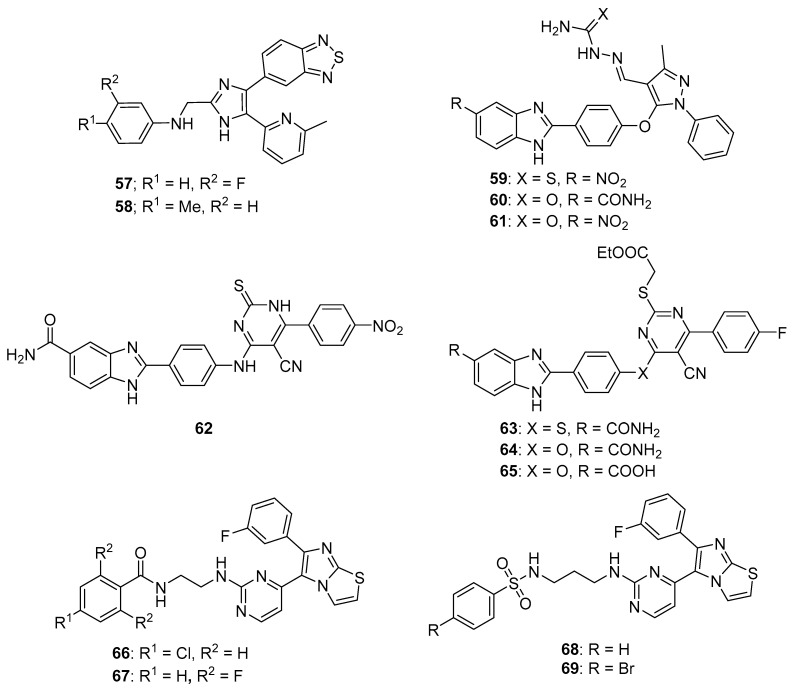
Imidazole-containing inhibitors of ALK5, Chk2, and BRAF reported in the literature (compounds **57**–**69**).

TGF-β modulates various cellular processes including cell proliferation, migration, differentiation and apoptosis. In a process that involves TGF-β binding to the TGF-β receptor-2 (TβRII), TGF-β receptor-1 (ALK5) is activated and phosphorylates Smad2 and Smad3, which then combine with Smad4 to modulate transcription. Although TGF-β acts as a tumor suppressor early in tumor development, it acts as a tumor promotor later in tumorigenesis. Thus, ALK5 inhibitors have been investigated as potential anticancer therapeutics (see Reference [69] for a review of TGF-β signaling and opportunities presented by this process for anticancer therapeutics). A series of novel benzothiadiazole-imidazole and thienopyridine-imidazole derivatives were synthesized by Guo et al. and evaluated for activity against ALK5 [70]. These compounds inhibited ALK5 kinase activity with IC_50_ values in the range between 0.008–0.043 µM, with compound **57** (see Figure 6 for structures of compounds **57**–**69**) displaying the highest potency against ALK5. Compound **57** displayed 19-fold selectivity for ALK5 compared to p38α MAP kinase, which possesses a kinase domain similar to that of ALK5. Compound **58** exhibited an IC_50_ value of 7.70 µM against p38α MAP kinase and 0.022 µM against ALK5 (selectivity of 350-fold). In terms of the SAR, imidazole-containing compounds possessing a benzothiadiazole ring were in general slightly more active compared to those bearing a thienopyridine ring, while substitutions on the aniline moiety had little effect on activity. Western blotting analysis revealed that treatment of HepG2 and SPC-A1 cells with 0.1 µM and 0.5 µM concentrations of compound **57** resulted in the inhibition of TGF-β1-stimulated phosphorylation of Smad2. In a wound healing assay, treatment with compound **57** at 2 µM and 10 µM inhibited TGF-β1-induced migration of HUVECs.

#### 3.2.2. Inhibitors of Checkpoint Kinases

The checkpoint kinases (Chk1 and Chk2) are intracellular serine/threonine protein kinases which play an important role in the DNA damage response pathway limiting cell cycle progression in the presence of such damage. Chk1 is activated by ATR (ataxia telangiectasia mutated and Rad3-related serine/threonine kinase), while Chk2 is activated by ATM (ataxia telangiectasia mutated serine/threonine kinase; see references [71,72] for reviews focused on these kinases). Activated checkpoint kinases are responsible for downstream phosphorylation processes causing cell-cycle arrest in the presence of DNA damage, leading to DNA repair. Considering the roles played by Chk1 and Chk2 in the response to DNA damage, inhibition of Chk1 and/or Chk2 may improve the efficacy of anticancer agents that damage DNA and are, thus, an attractive target for the development of new anticancer drugs (see Reference [73] for a review of Chk1 and Chk2 inhibition in cancer). The development of Chk inhibitors, such as UCN-01, LY2603618, PF477736, AZD7762, and MK-8776, have provided a path toward the discovery of new molecules targeting checkpoint kinases [71,73].

Analogs bearing a pyrazole-benzimidazole motif were synthesized by Galal et al. and were tested for their cytotoxic activity on MCF-7, HeLa, and HepG2 cell lines and were also tested in vitro for Chk2 inhibition [74]. Chk2 inhibitory activity was in the range 9.95–65.07 nM for these molecules. Compounds **59** and **60** were among the most potent of these derivatives against Chk2, exhibiting IC_50_ values of 11.49 and 11.13 nM, respectively. Assays on HepG2, HeLa, MCF-7 and baby hamster kidney (BHK) cells showed that compound **60** displayed a GI_50_ value of 6.5 μM against MCF-7 cells, while compounds **61** and **59** exhibited GI_50_ values of 11.7 μM and 6.6 μM against HeLa cells, respectively. Some of these analogs enhanced the activity of the cytotoxic anticancer agents doxorubicin and cisplatin in MCF-7 cells, while some did not. For example, a potentiation index of 276 was determined for the combination of doxorubicin with compound **61** in MCF-7 cells, while no potentiation was observed for doxorubicin paired with target compounds **59** and **60**. Of the compounds evaluated, **61** was the least toxic in vivo and was, thus, further tested alone and in combination with doxorubicin in an MNU-induced breast cancer model in rats. Serum Chk2 activity was lower in MNU-induced animals receiving compound **61** at an oral dose of 50 mg/kg/day for 10 days compared to MNU-treated animals that did not receive this compound, and the serum of MNU-induced animals receiving a combination of doxorubicin and compound **61** at this same dose displayed lower Chk2 activity compared to MNU-induced rats receiving doxorubicin alone.

These authors extended the work described above by synthesizing derivatives in which the pyrazole moiety was changed to pyrimidine [75]. These new derivatives inhibited Chk2 activity with IC_50_ values ranging from 5.55–46.2 nM. Compounds **62** and **63** exhibited IC_50_ values of 6.60 and 6.24 μM, respectively, against MCF-7 cells. Target compounds bearing a C5 amide and either an ether or amine linkage at the para position of the phenyl ring at the C2 position of the benzimidazole core potentiated the activity of doxorubicin and cisplatin against MCF-7 cells. On the other hand, C5 carboxylic acid compound **65** and C5 amide compounds with a thioether linkage at the para position of the phenyl ring at the C2 position of benzimidazole antagonized the effects of cisplatin and doxorubicin in MCF-7 cells. Western blotting studies confirmed the potentiation effect of compound **64** and the antagonistic activity of compound **65**, both tested at 0.1 µM concentrations. In these experiments, the combination of the former with cisplatin blocked Chk2 phosphorylation in MCF-7 cells, while the combination of the latter with cisplatin did not. The combination of 0.1 µM compound **64** with doxorubicin at its GI_50_ concentration also caused arrest of MCF-7 cells in the S phase of the cell cycle.

#### 3.2.3. Rapidly Accelerated Fibrosarcoma (RAF) Kinase Inhibitors

RAF kinases are serine/threonine protein kinases that are activated by the human oncogene RAS. RAF acts in the RAS-RAF-MEK-ERK pathway, which plays a role in cell differentiation, survival, growth, apoptosis, and other cellular processes. ERK ultimately phosphorylates multiple cytoplasmic and nuclear substrates, resulting in the effects mentioned above. The RAF family of kinases consists of ARAF, BRAF, and CRAF, with BRAF mutations commonly occurring in papillary thyroid cancers, melanomas, and hairy cell leukemias. Sorafenib (BAY 43-9006) is a first-generation RAF inhibitor approved by the U.S. FDA for the treatment for renal cancer and hepatocellular carcinoma (HCC). The approval of sorafenib, together with the approval of second-generation RAF inhibitors vemurafenib and dabrafenib which act on cancers harboring BRAF mutations, has sparked interest in the discovery of new molecules selectively targeting this pathway (see References [76,77] for reviews covering RAF signaling and inhibition in cancer). Abdel-Maksoud et al. synthesized a series of imidazothiazole derivatives and studied their BRAF inhibitory activity and cytotoxicity against the NCI 60 cancer cell line panel [78]. These compounds were most active against the colon cancer and melanoma sub-panels. IC_50_ values for several compounds were determined against the A375 and SKMEL-28 melanoma cell lines, with the most potent compounds **66** and **67** displaying IC_50_ values of 2.57 and 2.70 µM against A375 cancer cells, while compounds **66** and **68** possessed IC_50_ values of 8.16 and 6.85 µM against SKMEL-28 cells. In terms of the SAR for these molecules in the melanoma cell lines, among amide-containing compounds, derivatives containing a two carbon linker, such as **66** and **67**, showed greater potency compared with agents bearing three carbon linkers. In the case of sulfonamide derivatives, however, compounds with a three carbon linker were generally more potent than those bearing a two carbon linker. The most potent derivatives were tested in the in vitro kinase assay against ^WT^BRAF, ^V600E^BRAF, and CRAF, with the compounds displaying IC_50_ values in the range 9.30–312 nM. These compounds generally showed the highest potency against ^V600E^BRAF, with compound **69** displaying an IC_50_ value of 9.30 nM.

#### 3.2.4. Cyclin-Dependent Kinase (CDK) Inhibitors

The CDKs are an important class of serine/threonine kinases divided into two subfamilies: cell-cycle-related subfamilies (CDK1, 4, and 5) and transcriptional subfamilies (CDK7, 8, 9, 11, and 20; please see Reference [79] for a review of CDKs). Cell-cycle-related subfamilies control various transitional phases of the cell cycle, as well as mitotic progression and transcriptional subfamilies generally control the initiation and elongation of mRNA by the phosphorylation of RNA polymerase II, while some CDKs have a wider range of functions that are often tissue specific. Since cancer cells frequently show dysregulation in cell-cycle or transcriptional CDKs, inhibition of these enzymes may result in a selective antitumor effect (see References [80,81] for further information regarding CDKs in cancer and the use of CDK inhibitors as anticancer drugs).

**Figure 7:**
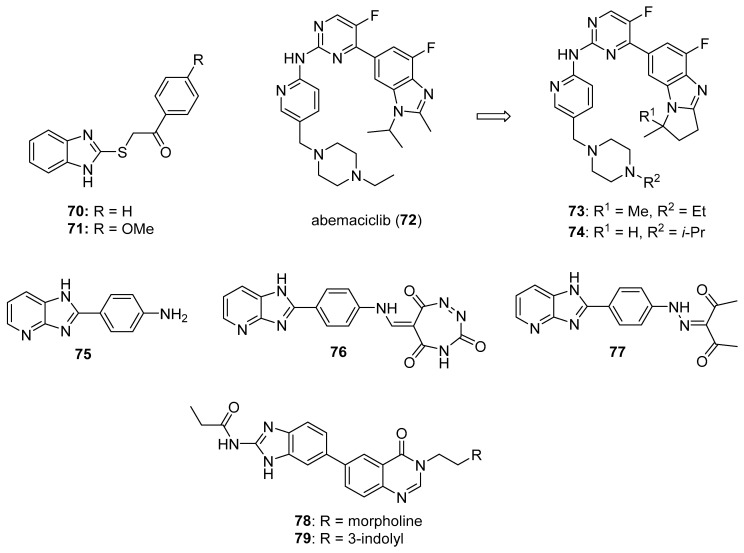
Additional imidazole-containing serine-threonine kinase inhibitors targeting CDK and AURK reported in the literature (compounds **70**–**79**).

CDK2 is involved in cell cycle regulation and is considered to be a member of the CDK1 subfamily [79]. Al-Warhi et al. synthesized six imidazole/benzimidazole thio-arylethanone derivatives as potential cytotoxic agents against T47D and MCF-7 breast cancer cell lines; these compounds were also tested for CDK2 inhibitory activity [82]. Among these molecules, imidazoles **70** and **71** (see Figure 7 for structures of compounds **70**–**79**) displayed the greatest potency. Compounds **70** and **71** exhibited IC_50_ values of 8.04 and 11.17 μM against T47D cells, respectively, while, against MCF-7 cells, the compounds displayed IC_50_ values of 12.90 and 4.53 μM, respectively. In an in vitro CDK2 assay, compound **70** exhibited an IC_50_ value of 0.89, while the corresponding IC_50_ value for compound **71** was 0.62 μM. Compounds **70** and **71** displayed docking energy scores of −8.7 and −8.9 kcal/mol when docked using the crystal structure of CDK2, while lower docking energy scores were obtained when these compounds were docked with crystal structures of CDK4/6, CDK7, and CDK9.

CDK4 and CDK6 promote progression through the G_1_ cell cycle phase, while CDK1 is critical in mitosis and is the only CDK that is essential for cell cycle progression in mammalian cells [81]. A series of analogs was designed based on the structural features of the approved drug abemaciclib (**72**), a selective oral CDK4/6 inhibitor indicated for metastatic breast cancer [83]. The synthesized compounds showed high selectivity toward CDK4/6 compared to CDK1/D3. Compounds **73** and **74** displayed high activity against CDK4/6 with IC_50_ values of 7.4/0.9 nM and 0.6/12.7 nM, respectively, and low activity against CDK1/D3 (IC_50_ values of 2670 and 3072 nM, respectively). Of these compounds, only **73** displayed an IC_50_ value against the MDB-MA-231 cancer cell line (232 nM) comparable to **72** (191 nM). Compound **73** was nearly twice as active as **72** in a hERG channel assay (IC_50_ values of 6.38 and 10.9 µM, respectively) and displayed pharmacokinetic and physicochemical properties similar to those of abemaciclib. Docking studies with **73** indicated binding of this compound to the catalytic domain of CDK6.

Ghanem et al. synthesized imidazopyridine derivatives and studied their inhibitory activity against CDK9, as well as their antiproliferative activities, on HCT116 and MCF-7 cell lines [84]. Compounds **75** and **76** were the most potent against the MCF-7 cell line, exhibiting IC_50_ values of 0.71 and 0.63 µM, respectively, while compound **77** displayed the greatest activity against the HCT116 cell line (IC_50_ = 1.69 µM). Compounds **75**, **76**, and **77** also exhibited IC_50_ values against CDK9 of 0.95, 0.50, and 1.00 µM, respectively, while sorafenib displayed an IC_50_ value of 0.76 µM in this assay. SAR analysis indicated that compounds containing an amine functionality *para* to the imidazopyridine attachment point generally displayed greater potency against the MCF-7 cell line compared to compounds bearing a diazo group at this position.

#### 3.2.5. Aurora Kinase (AURK) Inhibitors

Aurora kinases (A, B and, C) are cell cycle regulated serine-threonine kinases which play roles in different aspects of mitosis; the overexpression of AURKs is associated with wide variety of tumors. The inhibition of AURKs with small molecules, thus, represents an intriguing strategy towards the discovery of novel anticancer agents (see Reference [85] for a review of AURK inhibitors against cancer). Fan et al. synthesized a novel series of benzimidazole-quinazolinones and evaluated these molecules for cytotoxicity against MDA-MB-231, PC-3, and SH-SY5Y cancer cell lines [86]. The most potent molecule in this series, compound **78**, displayed IC_50_ values of 0.38, 1.09, and 0.77 µM against MDA-MB-231, PC-3, and SH-SY5Y cell lines, respectively. Potency was maintained when the morpholine side chain was replaced by an indole side chain, as compound **79** exhibited IC_50_ values of 0.55, 1.67, and 1.23 µM, respectively, on the three cell lines. While most of the compounds were active on all three cell lines, attaching the morpholino side chain via an amide linkage rather than a C-N linkage resulted in loss of activity. Both compounds **78** and **79** showed selectivity towards AURKA (IC_50_ values of 21.9 and 27.1 nM, respectively) compared to AURKB (IC_50_ values of 273 and 330 nM, respectively). Western blotting studies indicated a dose-dependent reduction in AURKA phosphorylation and histone H3 phosphorylation upon treatment of MDA-MB-231 cells with compound **78** at concentrations ranging from 1.0–4.0 µM.

#### 3.2.6. Nek2 Kinase Inhibitors

**Figure 8:**
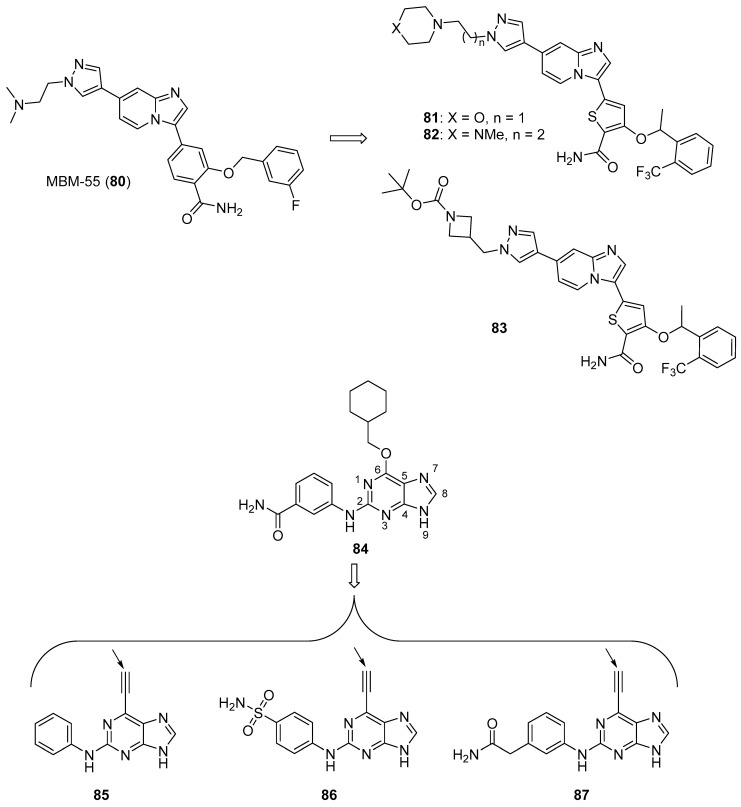
Imidazole-containing Nek2 kinase inhibitors reported in the literature (compounds **80**–**87**). For compounds **85**–**87**, the proposed sites for covalent inhibition of Nek2 kinase are indicated by the solid arrows.

Nek2 is a serine/threonine kinase involved in the regulation of several aspects of mitosis that is overexpressed in several cancers, making it an intriguing antineoplastic drug target (anticancer Nek2 inhibitors are reviewed by Fang and Zhang [87]). Wang et al. synthesized imidazopyridine derivatives based on the Nek2 kinase inhibitor MBM-55 (**80**) (see Figure 8 for structures of compounds **80**–**87**) and evaluated these compounds for activity against the MGC-803 gastric cancer cell line (high expression of Nek2 frequently occurs in gastric cancer cell lines) [88]. Compounds **81**, **82**, and **83** displayed potent anticancer activity against this cancer cell line, with IC_50_ values of 44, 54, and 38 nM, respectively. Compounds **81**–**83** were active against the HCT116 cell line (IC_50_ values ranging from 0.36–0.48 µM, while compound **80** displayed an IC_50_ = 0.70 µM), the Hep3B cell line (IC_50_ values ranging from 1.18–1.25 µM, while compound **80** exhibited an IC_50_ = 1.07 µM) and the BEL-7402 cell line (IC_50_ values ranging from 4.33–10.44 µM, while compound **80** displayed an IC_50_ = 6.37 µM). In general, compounds having amide substitutions on the thiophene ring were more potent compared to their ester analogs. Docking studies indicated that compound **83** forms an interaction with Nek2 similar to that shown by compound **80**.

Matheson et al. designed and synthesized novel molecules targeting Nek2 based on a co-crystal structure of compound **84**, a non-covalent reversible Nek2 inhibitor, bound to Nek2 [89]. Through examination of this structure, the authors hypothesized that the replacement of the alkoxy group at the purine C6 position of compound **84** with an ethynyl group could result in covalent binding of the ligand to the nearby Cys22 residue of Nek2. Target compounds **85** and **86** both displayed IC_50_ values of 140 nM against Nek2. Incubation of compound **86** with Nek2 at 10× its IC_50_ concentration followed by rapid dilution of the Nek2 sample resulted in 3% recovery of activity compared to 11% recovery of activity after treatment of Nek2 with control competitive inhibitors, providing evidence for the irreversible inhibition of Nek2 by compound **86**. Compound **85** displayed weak competitive inhibition of Nek2-C22A (in which the Cys22 residue of Nek2 was mutated to Ala) with an IC_50_ value of 3.5 μM. When compounds **85** and **86** were tested against a panel of Ser-Thr kinases, they typically displayed >10-fold selectivity for Nek2 over other kinases, although this was not the case for the inhibition of Cdk2 by compound **86** (IC_50_ = 0.84 μM). Target compound **85** displayed GI_50_ values of 1.8, 1.2, 1.1, and 0.2 μM against U2OS, HeLa, MDA-MB-231, and HEK-293 cell lines, respectively, while compound **86** was inactive against U2OS and HeLa cells (presumably due to a lack of cell penetration). The synthesis of analogs based on **85** provided compounds generally displaying mid-nanomolar IC_50_ values against Nek2, but a poor correlation was observed between Nek2 activity and cellular GI_50_ values against the SK-BR-3 cell line. Nonetheless, compound **87** (Nek2 IC_50_ = 0.062 μM, cellular SK-BR-3 GI_50_ = 2.2 μM) displayed selectivity for Nek2 when this compound was profiled against a panel of 121 kinases, and compounds **85** and **87** inhibited the phosphorylation of C-Nap1, a specific substrate of Nek2, in U2OS cells. X-ray co-crystal structures of **86** and **87** complexed with Nek2 confirmed that the ethynyl group of these ligands both formed a covalent bond with Cys22 of Nek2.

### 3.3. Phosphatidylinositol-3-Kinase (PI3K)/AKT/mTOR Inhibitors

Signaling through the PI3K/AKT/mTOR pathway is initiated by receptor tyrosine kinases and G-protein coupled receptors (GPCRs) present on the cell surface. Kinase interactions downstream of PI3K are known to involve a complex signaling cascade which influences cell proliferation, cell cycle progression, cell growth, and cell survival. Dysregulation of the PI3K/AKT/mTOR signaling pathway has been found in most cancers, such as breast, lung, blood, ovarian, and colorectal malignancies, indicating that this pathway is worthy of exploration in a wide range of cancers (please see References [77,90] for reviews of PI3K signaling and inhibition). The PI3K inhibitors idelalisib and copanlisib are approved for the treatment of leukemia and lymphoma [91], while the PI3Kα inhibitor alpelisib has been approved in combination with fulvestrant for the treatment of certain types of breast cancer [92]. These successes, together with the desire to improve side effect profiles, have driven continued research on compounds that affect the PI3K/AKT/mTOR pathway.

**Figure 9:**
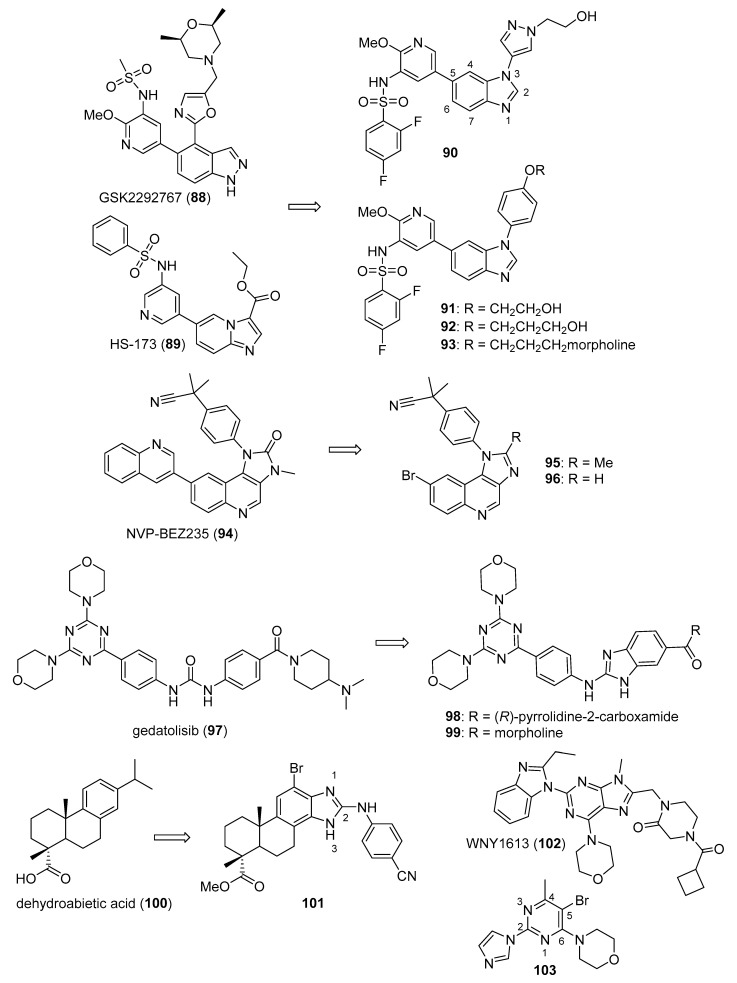
Proposed imidazole-containing PI3K/AKT/mTOR inhibitors reported in the literature (compounds **88**–**103**).

Ding et al. synthesized a novel series of disubstituted benzimidazoles based on fragment features taken from GSK2292767 (**88**) and the previously reported PI3K inhibitor HS-173 (**89**) (structures for compounds **88**–**103** are provided in Figure 9) possessing potent anticancer activity towards the T47D, MCF-7, and HCT116 cell lines [93]. Most of the synthesized compounds were active against these cell lines, possessing IC_50_ values below 5.0 µM. Compounds **90** (IC_50_ values of 0.27, 0.57, and 0.13 µM), **91** (IC_50_ values of 0.36, 0.31, and 0.14 µM), **92** (IC_50_ values of 0.45, 0.59, and 0.85 µM), and **93** (IC_50_ values of 0.29, 0.41 and 0.34 µM) were among the most potent compounds against the three cell lines, respectively. Compounds **91** and **92** showed improved activity against PI3Kα, PI3Kβ, PI3Kγ, and PI3Kδ when compared to **89**, with IC_50_ values ranging from 0.50–5.5 nM. Western blotting analysis indicated that compound **91** inhibited AKT phosphorylation when incubated with HCT116 cells at a 3 µM concentration.

Xiao et al. synthesized imidazoquinoline derivatives and screened them for antitumor activity against PC-3, HepG2, A549, and MCF-7 cell lines [94]. Compounds **95** and **96** displayed activity against HepG2, A549, and PC-3 cell lines with IC_50_ values of 1.43, 13.43, 6.67 µM and 2.42, 6.29, 5.11 µM, respectively. The dual PI3K-mTOR inhibitor NVP-BEZ235 (**94**) displayed IC_50_ values of 0.54, 0.36, and 0.20 µM against HepG2, A549, and PC-3 cell lines, respectively. Compounds **95** and **96** inhibited PI3Kα (IC_50_: 6.8, 0.9 µM) and mTOR kinases (IC_50_: 4.6, 1.4 µM). Since the IC_50_ values of **94** against PI3Kα and mTOR kinases were 4 and 6 nM, respectively, compounds **95** and **96** may display anticancer activity through other mechanisms. SAR studies showed that the placement of bromine at the C6 position of quinoline is essential for activity.

Wu et al. synthesized a novel series of molecules possessing a triazine-benzimidazole scaffold based on the PI3K inhibitor gedatolisib (**97**) designed to act as potential anticancer agents through the inhibition of PI3K/mTOR [95]. All but one of the target compounds inhibited PI3K and mTOR with IC_50_ values below 200 nM. Compounds **98** and **99** were among the most potent target compounds and were more active towards PI3Kδ compared to other PI3K isoforms, with IC_50_ values of 5.1 and 13.0 nM, respectively, compared to **97** (IC_50_ = 156 nM). Compounds **98** and **99** displayed IC_50_ values of 0.9 and 0.3 µM against the HCT116 cell line, while **97** displayed an IC_50_ value of 1.4 µM. Western blotting studies in this cell line showed that 10 µM concentrations of compounds **98** and **99** almost completely inhibited the phosphorylation of AKT and p70S6K, the latter protein being downstream of the PI3K/AKT/mTOR signaling pathway.

A series of arylthio- and arylamino-benzimidazole derivatives of dehydroabietic acid (**100**) synthesized by Yang et al. were tested for PI3K inhibition, as well as activity against HCT116, MCF-7, HeLa, and HepG2 cancer cell lines [96]. Counter-screening was also performed against a normal human gastric mucosal cell line (GES-1). Among these compounds, **101** displayed the highest potency, with IC_50_ values of 0.18, 0.43, 0.71, and 0.63 μM against these four cancer cell lines, respectively. Compound **101** also displayed lower cytotoxicity against the GES-1 cell line, with an IC_50_ value of 22.0 μM. In general, compounds having 2-arylthio substitutions at the benzimidazole C2 position were less potent compared to 2-arylamino derivatives. Moreover, compounds substituted with electron-withdrawing substituents at the para position of the arylamino or arylthio rings displayed stronger activities. Compound **101** displayed IC_50_ values of 0.012, 0.21, 0.18, and 0.11 against PI3Kα, β, γ, and δ, respectively, while it was inactive against mTOR (IC_50_ > 10 μM). Western blotting studies with compound **101** in HCT116 cells showed that **101** inhibited AKT phosphorylation in a dose-dependent manner at concentrations of 0.5 and 1.0 μM.

Zuo et al. synthesized piperazinone-purine hybrids as selective PI3Kδ inhibitors targeting non-Hodgkin’s lymphomas [97]. WNY1613 (**102**) displayed an IC_50_ value of 1.2 nM against PI3Kδ and selectivity indexes (IC_50_ vs. the kinase of interest/IC_50_ vs. PI3Kδ) of 219, 120, and 250 relative to PI3Kα, PI3Kβ, and PI3Kγ, respectively. Moreover, this compound only displayed activity >50% against PI3K isoforms when tested at a concentration of 500 nM against a panel of 300 other kinases. Compound **102** displayed antiproliferative activity against the SU-DHL-4 and SU-DHL-6 (B-cell lymphoma) cell lines (IC_50_ values of 0.048 and 0.038 nM), and also exhibited an IC_50_ value of 0.077 μM against the JEKO-1 mantle cell lymphoma line. The reference drug idelalisib displayed IC_50_ values of 0.30, 0.12, and 0.12 μM against these cell lines, respectively. Western blotting analysis with compound **102** in SU-DHL-4, SU-DHL-6, and JEKO-1 cell lines revealed a dose and time dependent inhibition of phosphorylation of S6, AKT, and 4EBP1, all downstream components of PI3K signaling. In xenograft models employing JEKO-1 and SU-DHL-6 cells in non-obese diabetic SCID mice, idelalisib and compound **102** administered orally at 25 mg/kg for 18 days displayed tumor growth inhibition of 45.9% and 55.5% for the JEKO-1 model and 47.7% and 51.4% for the SU-DHL-6 model compared to the control groups, respectively. Increasing the dose of compound **102** to 50 mg/kg did not result in a significant improvement of its antitumor effect.

Gaonkar et al. synthesized substituted pyrimidine derivatives as antiproliferative agents that were evaluated against HeLa, MCF-7, HepG2, NCI-H460, and IMR-32 cancer cell lines [98]. Target compound **103** contained imidazole at the C2 position of the pyrimidine core and displayed the greatest potency against HeLa cells (IC_50_ = 5.88 μM). Treatment of HeLa cells with compound **103** at 5, 10, and 20 µM concentrations resulted in the dose-dependent accumulation of cells in the sub-G_1_ phase of cell cycle and the induction of apoptotic cell death. Since morpholinopyrimidines, such as apitolisib (GDC-0980), have shown activity as PI3K/mTOR inhibitors [99], molecular docking studies were performed between compound **103** and mTOR’s catalytic domain. The authors reported several interactions between compound **103** and amino acids at the ATP binding pocket of mTOR.

## 4. Imidazoles as Inhibitors of Other Targets

### 4.1. DNA Intercalators

Intercalators are classes of small molecules that insert between adjacent DNA base pairs, causing significant deformation of the double helix. These compounds typically contain planar polyaromatic regions which stack with DNA bases, but intercalators may also make ionic and hydrogen bonding interactions with DNA. Due to these alterations in DNA, intercalators may interfere with replication, transcription, cell growth, and cell division. Interest in DNA intercalators as potential anticancer agents remains robust (see References [100,101] for reviews of DNA intercalating agents), and a better understanding of the interactions between DNA and anticancer candidates may help medicinal chemists to design intercalating molecules with improved anticancer activity.

**Figure 10:**
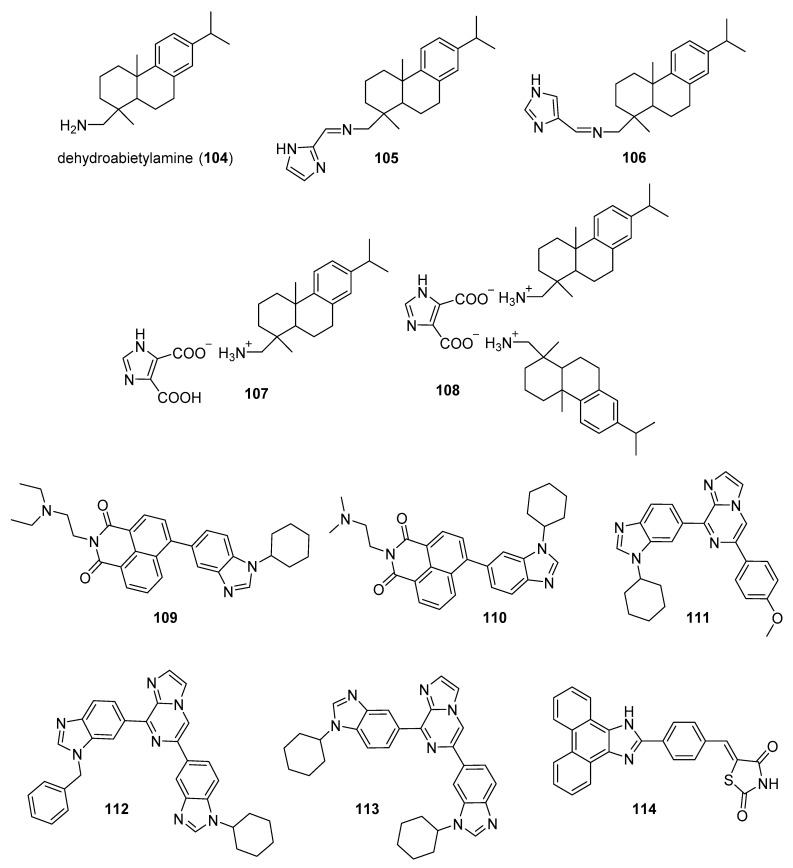
Imidazole-containing compounds reported as DNA intercalators (compounds **104**–**114**).

Zhao et al. synthesized Schiff base derivatives of dehydroabietylamine (**104**) (see Figure 10 for structures of compounds **104**–**114**) as potential anticancer agents and evaluated these compounds for activity against MCF-7, A549, HeLa, and HepG2 cancer cell lines, with HUVECs used as a normal reference cell line [102]. Most of the derivatives displayed IC_50_ values ranging from 2.5–15 µM against MCF-7 cells, while compounds **105** and **106** exhibited the highest potency against HepG2 cells (IC_50_ values of 0.24 and 0.14 µM, respectively). By comparison, **104** displayed an IC_50_ value of 2.56 µM against HepG2 cells. Interestingly, **106** was 180 times more potent against HepG2 cells compared to HUVECs. Addition of compounds **105** and **106** to salmon sperm DNA complexed with ethidium bromide resulted in a reduction in fluorescence, consistent with binding of compounds **105** and **106** to DNA. A hypochromic shift was observed upon adding more DNA to solutions containing compounds **105** and **106**, consistent with the hypothesis that these compounds bind to DNA via intercalation.

Further extension of the work by Zhao et al. conveyed the synthesis of imidazole derivatives of compound **104** (organic salts and amides) [103]. All the compounds derived from **104** were more potent against MCF-7 cancer cells compared to the parent compound. Among these, compounds **107** and **108** displayed IC_50_ values of 6.47 and 0.75 µM against MCF-7 cells compared to compound **104** (IC_50_ = 19.45 µM). Compound **107** displayed greater potency against A549 cells (IC_50_ = 1.85 µM) compared to compound **104** (IC_50_ = 5.02 µM). As above, spectroscopic experiments were used to provide evidence for the binding of these compounds to DNA via intercalation.

A series of compounds reported by Singh et al. explored the benzimidazole scaffold for the synthesis of DNA-interactive anticancer candidates [104,105,106,107]. In one such effort, naphthalimide–benzimidazole hybrid compounds were prepared as potential antiproliferative agents against the NCI panel of 60 human cancer cell lines [104]. Based on the evaluation of 18 target compounds against this panel at a single 10 µM dose, compounds **109** and **110** were chosen for testing against the full panel of cell lines at concentrations ranging from 0.01 to 100 µM. Mean graph midpoint (GI_50_) values of 1.43 and 1.83 μM were determined for compounds **109** and **110**, respectively, with GI_50_ values ranging from 0.372–2.23 µM for compound **109** and 0.422–3.95 µM for compound **110** against the sixty cell line panel. Compounds **109** and **110** increased the melting temperature of calf thymus DNA by 14.2 and 5.9 °C, respectively, while circular dichroism studies indicated that these compounds bound to calf thymus DNA via intercalation.

Benzimidazole-imidazopyrazine conjugates were synthesized and were also evaluated for their antiproliferative activity on the NCI 60 panel of cancer cell lines [105]. Compound **111** showed GI_50_ values ranging between 312–890 nM against K562 and SR leukemia, NCI-H460 and NCI-H522 non-small cell lung cancer, HCT116 and HT29 colon cancer, SF-295 CNS cancer, and MDA-MB-435 melanoma cancer cell lines. Compound **112** displayed a GI_50_ value of 799 nM against the 786-0 renal cancer cell line. Spectroscopic studies indicated the binding of compounds **111** and **112** to calf thymus DNA. In thermal denaturation studies, compounds **111** and **112** displayed Δ*Tm* values of 22 °C and 12 °C, respectively, in the presence of calf thymus DNA, indicating that compound **109** has a strong affinity for DNA. Exposure to increasing concentrations of compounds **111** and **112** caused a dose-dependent decrease in the fluorescence intensity of the ethidium bromide-DNA complex, indicating displacement of ethidium bromide from DNA by these compounds. Circular dichroism studies with compounds **111** and **112** in the presence of calf thymus DNA suggested an intercalative mode of DNA binding for these molecules. In an extension of the work described above, compound **113** displayed broad spectrum activity with GI_50_ values in the range between 0.80–2.87 μM against 59 human cancer cell lines [106]. This compound increased the melting temperature of calf thymus DNA by 12 °C. A binding constant (K_b_) of 1.25 × 10^4^ M^−1^ was determined for the interaction of compound **113** with calf thymus DNA, and spectroscopic studies indicated that this compound bound to DNA via intercalation. Compound **113** displayed effective binding with bovine serum albumin (BSA) (K_b_ = 3.79 × 10^4^ M^−1^), indicating that albumin may be capable of transporting this molecule in the blood.

Singh et al. also synthesized a novel series of phenanthrene/acenaphthalene-fused imidazole derivatives which were screened for in vitro antitumor activity against the NCI 60 cancer cell line panel [107]. At a concentration of 10 µM, compound **114** displayed >50% inhibition against leukemia (HL-60, K562, CCRF-CEM, RPMI8226, and SR), colon (HCT116 and HCT-15), melanoma (MDA-MB-435), renal (A498), prostate (PC-3), and breast (BT-549 and MDA-MB-468) cancer cell lines. Compound **114** exhibited an IC_50_ value of 1.70 µM against A549 cells but showed little effect on the HEK-293 cell line at a concentration of 100 µM. Spectroscopic studies showed that compound **114** bound to DNA via intercalation. The binding constant (K_b_) between compound **114** and BSA was 6.57 × 10^4^ M^−1^, while the corresponding K_b_ for the human serum albumin-compound **114** complex was 9.35 × 10^4^ M^−1^.

### 4.2. G-Quadruplex Stabilizers

G-quadruplexes (G4s) are four-stranded guanine-rich sequences present in the telomere and promotor regions of the human genome that play important roles regulating replication, transcription, and translation. The level of G4s is elevated in tumors and G4s frequently occur in proto-oncogenes; stabilization of these sequences by G4 ligands may block replication, transcription, and translation that is vital to cancer cells (see Reference [108] for a review of G-quadruplexes in cancer). For example, the c-MYC protein, which is dysregulated in several cancers, is coded by a gene that contains a G-rich promoter element that forms G4s [109]. Stabilization of *c-MYC* G4s with a small molecule has been shown to be promising anticancer strategy [110]. The design of ligands that bind to G-quadruplexes and regulate various genetic functions through stabilization of G4s has, thus, become an interesting focus area for anticancer drug discovery.

**Figure 11:**
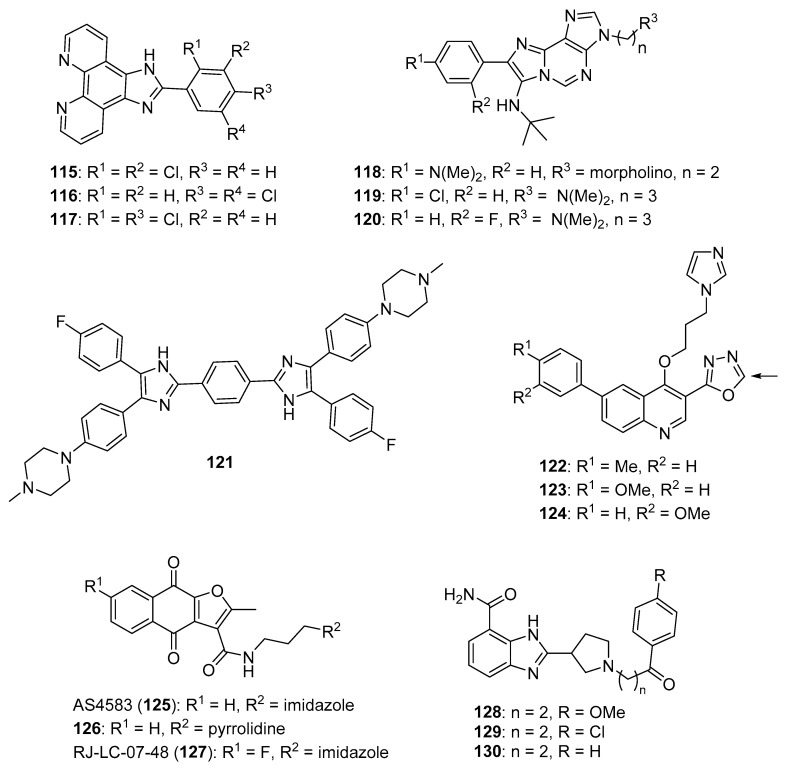
Imidazole-containing compounds and their derivatives targeting G4s, TPI, MCMs, and PARP (compounds **115**–**130**). For the scaffold represented by compounds **122**–**124**, the arrow indicates the position where loss of activity was observed upon modification.

Phenanthroimidazole derivatives were synthesized by Wu et al. as potential anticancer agents targeting *c-MYC* G-quadruplexes [111]. Compound **115** (see Figure 11 for structures of compounds **115**–**130**) displayed an IC_50_ value of 1.1 µM against the CNE-1 cell line, while compound **116** exhibited an IC_50_ value of 0.9 µM against the MCF-7 breast cancer cell line. Binding of compounds **115**, **116**, and **117** to *c-MYC* G4 DNA was demonstrated by hypochromic shifts observed upon addition of this DNA to these compounds at a concentration of 20 µM. Western blotting experiments showed that treatment of CNE-1 cells with compound **115** at concentrations of 0.5–2.0 µM resulted in decreased expression of the c-MYC protein in a dose-dependent manner. When given at doses of 3 and 6 µM, compound **115** inhibited the migration and proliferation of CNE-1 cells in a zebrafish xenograft model of nasopharyngeal carcinoma.

Pelliccia et al. synthesized imidazopurine derivatives as potential G4-targeting anticancer agents [112]. The authors initially synthesized compound **118**, which displayed a significant effect on the melting temperature (Δ*Tm*) of G4 forming DNA sequences from oncogene promotors (Δ*Tm* for *BCL2* G4, *c-MYC* G4, and *c-KIT1* G4 of 9.2, 7.2, and 5.2 °C, respectively), while increasing the Δ*Tm* of telomeric G4 *Tel23* by only 2.4 °C. Moreover, compounds **119** and **120** were synthesized as part of a focused library of analogs of **118**; these compounds showed selectivity for increasing the melting temperature of *BCL2* and *c-MYC* G4 regions over telomeric G4 *Tel23* and duplex DNA. Compound **119** displayed an IC_50_ value of 17 µM against the Jurkat cell line but showed little effect on the growth of MCF-7, HCT116, or A375 cancer cell lines or against nonmalignant human dermal fibroblasts (HDF) or human keratinocytes (HaCaT). Compound **120** decreased the survival of Jurkat cells by approximately 60% at a concentration of 50 µM. Reduction of *c-MYC* expression by 66% and 56% was observed by qPCR when Jurkat cells were exposed to 25 µM concentrations of compounds **119** and **120**, respectively. Similarly, treatment with compounds **119** and **120** caused 67% and 43% inhibition of *BCL2* expression in Jurkat cells. Western blotting analysis revealed that exposure of Jurkat cells to compound **119** at a concentration of 25 µM reduced c-MYC and BCL-2 protein levels by approximately 50% and 40%, respectively, with compound **120** having less effect on the expression of these proteins.

In a subset of cancers, a telomerase-independent mechanism (alternative lengthening of telomeres or the ALT pathway) maintains telomeres (reviewed in Reference [113]). Hu et al. synthesized dimeric arylimidazole derivatives designed to target telomeric G4s, which are multimeric, and inhibit the ALT pathway [114]. Among these target molecules, compound **121** displayed high selectivity towards a multimeric G4 sequence (*tel45*) over a monomeric G4 sequence (*tel21*). Compound **121** displayed Δ*T_m_* values of 12.7 °C and 1.7 °C for *tel45* and *tel21*, respectively, in line with the smaller dissociation constant determined for the DIZ-3/*tel45* complex (4.6 µM) compared to the DIZ-3/*tel21* complex (18.4 µM). The results of titration experiments with **121** together with *tel45* substituted with the fluorescent base analog 2-aminopurine were consistent with **121** binding to *tel45* via intercalation. **121** displayed an IC_50_ value of 2.1 μM against U2OS cells, an ALT cancer cell line, while **121** exhibited an IC_50_ value of 29.3 μM against normal BJ fibroblasts. This compound did not inhibit the transcription of the G4-driven oncogenes *c-MYC*, *HRAS*, *VEGF*, *BCL-2*, and *c-KIT* in U2OS cells, indicating its selectivity for multimeric G4s.

### 4.3. Topoisomerase Inhibitors

DNA topoisomerases play important roles in modifying DNA topology, with type 1 topoisomerases (TopI, which make single strand cuts in DNA) and type II topoisomerases (TopII, which result in DNA double strand cleavage) both serving as targets for clinical anticancer drugs. Anticancer agents, such as doxorubicin, etoposide, and topotecan, promote topoisomerase-mediated DNA cleavage, while inhibitors of catalytic function have not enjoyed significant clinical success (see Reference [115] for a review of topoisomerases in cancer). Despite the clinical efficacy of these topoisomerase-targeted agents, their shortcomings, such as dose-limiting toxicities, drug resistance, and poor solubility, limit their use, leading to the exploration of additional compounds designed to target DNA topoisomerases (see Reference [116] for a recent review of anticancer topoisomerase inhibitors). Kundu et al. tested the activity of novel quinoline-based compounds bearing an imidazole side chain in vitro against recombinant TopI and on TopI activity in MCF-7 cellular lysates [117]. Compounds **122**, **123**, and **124** displayed IC_50_ values against recombinant TopI of 1.06, 0.029, and 1.054 µM, respectively, and IC_50_ values against this activity in MCF-7 lysates of 3.75, 2.74, and 4.11 µM, respectively. SAR results show that substitution at the oxadiazole position indicated by an arrow with hydrophilic and hydrophobic groups, such as amino or methyl, caused a drastic loss of activity. Compound **123** stabilized TopI-mediated cleavable complex in MCF-7 cells at a concentration of 5 µM, and treatment of MCF-7 cells with this compound also resulted in the formation of less reversible TopI-induced DNA strand breaks compared to the known TopI modulator camptothecin. Results from DNA unwinding assays and ethidium bromide (EtBr) displacement assays suggested that compound **123** was not a DNA intercalator. Further, compound **123** displayed IC_50_ values of 2.74, 2.61, 2.34, and 2.35 μM against MCF-7, HeLa, HCT116, and NIH:OVCAR-3 cancer cell lines and an IC_50_ value of 8.34 μM against the HEK-293 kidney cell line.

### 4.4. Inhibitors of Minichromosomal Maintenance Proteins (MCMs)

MCMs display helicase activity and play important roles in DNA replication and cell cycle progression (a review of these proteins is given in Yu et al. [118]), and overexpression of MCMs has been linked to poor outcomes in cancer [119]. The search to find novel molecules which target MCMs is, therefore, an emerging area in anticancer drug discovery. High-throughput screening of a library containing two million molecules performed by Lin et al. identified the furanonaphthoquinone-based molecule AS4583 (**125**) that acted as a potent antiproliferative agent in tyrosine kinase inhibitor (TKI)-sensitive and TKI-resistant NSCLC cells (IC_50_ = 77 nM) by targeting the MCM complex [120]. Treatment of athymic nude mice bearing H1975 tumors with compound **125** for 5 days a week over 4 weeks resulted in reduced tumor volumes compared to the control group (44% and 59% reduction compared to control at 1 and 4 mg/kg, respectively). Compound **125** interfered with DNA replication by inhibiting the formation of replication forks in H1975 cells as determined by immunofluorescence staining of MCM2. Moreover, this compound promoted the dose-depended degradation of MCM2, MCM6, and MCM7 in H1975, H3255, and PC9 lung cancer cell lines at concentrations of 100 and 200 nM. In terms of the SAR, the benzoquinone moiety and the furan oxygen atom were essential for activity. Substitution of imidazole with pyrrole or a phenyl group decreased the antiproliferative activity. Replacement of imidazole with pyrrolidine (compound **126**) led to an increase in potency (IC_50_ = 24 nM), but this compound displayed toxicity against Hs68 skin fibroblasts (IC_50_: 77 nM). The compound **125** analog RJ-LC-07-48 (**127**) displayed high potency with an IC_50_ value of 17 nM against H1975 cells. When given by i.p. injection for 4 weeks, administration of compound **127** in the H1975 murine xenograft model resulted in average tumor volumes of 128.2 mm^3^ and 109.5 mm^3^ at doses of 1 mg/kg and 5 mg/kg in these groups, respectively, while the average tumor volume was 546.2 mm^3^ in the control group.

### 4.5. Poly(ADP-Ribose)polymerase (PARP) Inhibitors

PARP family enzymes play an important role in DNA repair, especially the repair of single strand breaks or the excision of a DNA base, by the transfer of ADP-ribose to target proteins from the substrate NAD^+^. If PARP activity is inhibited during DNA repair, single strand breaks result in the formation of double strand breaks (DSBs). Cells exhibiting DSBs require homologous recombination (HR) for DNA repair, making tumor cells that are deficient in HR (those harboring *BRCA1* or *BRCA2* mutations) sensitive to PARP inhibition. PARP is, thus, an inviting target in anticancer drug discovery (see References [121,122] for recent reviews on PARP and PARP inhibitors). Benzimidazole carboxamide derivatives developed by Min et al. were tested for PARP inhibition, as well as for cytotoxicity, against MDA-MB-436 (a *BRCA1* mutant breast cancer cell line) and Capan-1 (a *BRAC2*-deficient pancreatic cancer cell line) [123]. Compounds **128**, **129**, and **130** were excellent PARP-1 and PARP-2 inhibitors, with IC_50_ values of 5.9 and 4.5 nM, 3.9 and 4.2 nM, and 3.6 and 3.2 nM, respectively, whereas the known PARP inhibitor veliparib exhibited IC_50_ values of 5.3 and 1.6 nM against PARP-1 and PARP-2. Compounds **129** and **130** displayed the highest potencies against the MDA-MB-436 and Capan-1 cell lines, with IC_50_ values ranging from 11.4–19.8 µM, while the IC_50_ values for veliparib were >100 µM against these cell lines. In this series, compounds possessing a phenyl ketone moiety exhibited the greatest potency against these cell lines, irrespective of the two to four carbon length of the linker between the pyrrolidine and phenyl rings.

### 4.6. Histone Deacetylase (HDAC) Inhibitors

Histone deacetylases (HDACs) reverse the action of histone acetyltransferases by removing acetyl groups from lysine residues of both histone and nonhistone proteins. There are four classes of HDACs that deacylate lysine substrates via a Zn^2+^ or NAD^+^ dependent mechanism, and the abnormal expression of HDACs has been reported in various types of cancers. HDAC inhibitors have emerged as a class of anticancer agents capable of regulating gene expression, inhibiting DNA repair, causing growth arrest, inducing apoptosis, and inhibiting angiogenesis (see References [124,125,126] for reviews of HDACs and anticancer HDAC inhibitors). The utility of FDA-approved drugs, such as vorinostat, romidepsin, belinostat, and panobinostat, along with the challenges remaining concerning the use of these HDAC inhibitors for cancer treatment, have encouraged the design and synthesis of new compounds as selective HDAC inhibitors [127].

**Figure 12:**
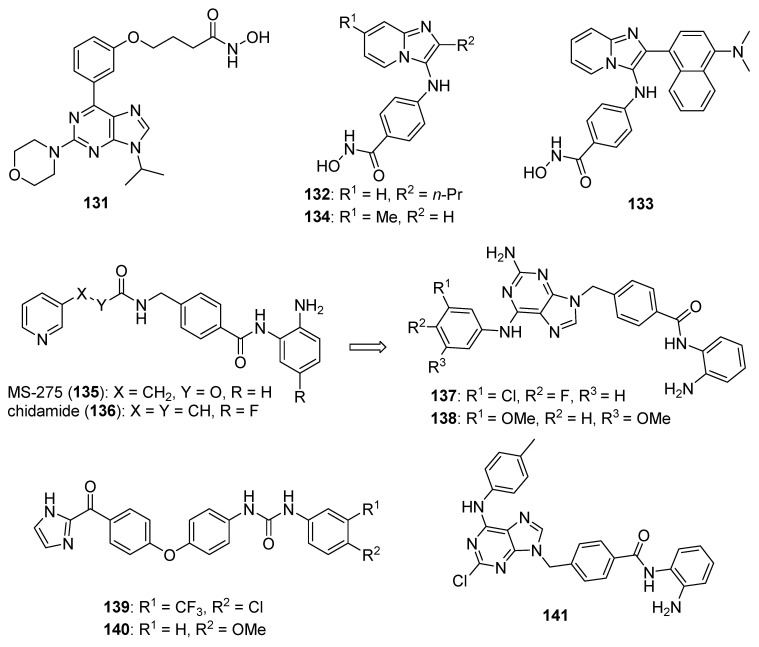
Imidazole-containing HDAC inhibitors reported in the literature (compounds **131**–**141**).

Chen et al. prepared thirty-one 6-phenylpurines linked to hydroxamate via an alkoxy chain of variable length [128]. Compound **131** (see Figure 12 for structures of compounds **131**–**141**) exhibited promising activity against MV4-11 and PC-3 cancer cell lines, with IC_50_ values of 0.16 and 1.16 µM, respectively. This compound also showed excellent potency as an inhibitor of HDAC activity in HeLa cell nuclear extracts, with an IC_50_ value of 35 nM. By comparison, the known HDAC inhibitor vorinostat displayed an IC_50_ value of 57 nM against HeLa nuclear extract-derived HDAC activity and IC_50_ values of 0.36 and 1.49 µM against MV4-11 and PC-3 cancer cell lines, respectively. For these thirty-one derivatives, a positive correlation was shown between HDAC IC_50_ and antiproliferative potency against the MV4-11 cell line, consistent with the hypothesis that the compounds evaluated target HDACs in these cancer cells. Compound **131** was also found to be a strong inhibitor of class I and IIb HDACs but a weak inhibitor of class IIa and IV HDACs. When compound **131** was evaluated against a panel of 97 kinases, ≥50% inhibitory activity was not observed for any of these kinases at a 1 µM concentration of this compound. Histone H3 acetylation was increased in BALB/c nude mice bearing PC-3 tumors when compound **131** was administered orally to these animals at a dose of 150 mg/kg.

Due to the proposed unique roles of HDAC6 in carcinogenesis (reviewed in Reference [129]), HDAC6 inhibitors are of interest as potential selective anticancer agents. Mackwitz et al. reported a multicomponent reaction using aldehydes, 2-aminopyridines, and methyl 4-isocyanobenzoate under microwave irradiation as a key step to access new imidazopyridine derivatives as candidate HDAC6 inhibitors [130]. These derivatives were screened for inhibition of human recombinant HDAC1 and HDAC6, as well as for activity against a human tongue squamous cell carcinoma cell line (CAL27). Compound **132** displayed IC_50_ values of 2.20 µM against HDAC1 and 58 nM against HDAC6 (selectivity index = 38). Compound **133** displayed the best activity in a whole-cell HDAC inhibition assay employing CAL27 cells with an IC_50_ value of 1.75 µM. In terms of the SAR for these compounds, derivatives bearing an alkyl group at C7 of the imidazopyridine core displayed the highest selectivity for HDAC6 over HDAC1. The substituent present at C2 was less important in dictating cellular activity, however, as a subset of target compounds bearing substituents ranging from H (**134**) to 4-(dimethylamino)-1-naphthyl (**133**) at C2 displayed IC_50_ values in a narrow range (3.22–4.78 µM) against CAL27 cells. The X-ray crystal structure of **132** bound to catalytic domain 2 from *Danio rerio* (zebrafish) HDAC6 revealed that the hydroxamate group of **132** was coordinated to the catalytic zinc ion.

Nepali et al. designed a new series of purine-benzamide compounds based on MS-275 (**135**) and chidamide (**136**). These compounds were evaluated for their action on MDA-MB-231, MDA-MB-468, and HepG2 cell lines and also for HDAC inhibition [131]. Target compound **137** displayed IC_50_ values of 1.48, 0.65, and 2.44 μM against the MDA-MB-231, MDA-MB-468, and HepG2 cell lines, respectively, while target compound **138** displayed IC_50_ values of 3.17 and 4.20 μM against the MDA-MB-231 and HepG2 cell lines. By comparison, **135** and **136** displayed IC_50_ values of 2.60 and 3.60 μM against the MDA-MB-231 cell line, respectively, and 4.54 μM and >8 μM against HepG2 cells, respectively. When tested against **135** sensitive (YCC11) and **135** resistant (YCC3/7) gastric cancer cells, compound **137** displayed IC_50_ values of 4.77 and 4.79 μM, respectively, while the corresponding IC_50_ values for **135** were 6.03 and 12.98 μM, respectively. Compound **137** displayed selectivity towards HDAC1, 2, and 3, exhibiting IC_50_ values in the range 0.108–0.585 μM, while exhibiting IC_50_ values > 5 μM against HDAC4-10. Western blotting studies revealed a dose-dependent elevation in histone H3 acetylation due to the incubation of compound **137** with MDA-MB-231 cells at concentrations ranging from 0.5–5 μM. In an MDA-MB-231 breast cancer xenograft model in mice, 56.3% tumor growth inhibition was observed compared to the control group when compound **137** was administered at a dose of 50 mg/kg by i.p. injection daily for 23 days.

Novel benzoylimidazole derivatives were synthesized as dual RAF/HDAC inhibitors and were screened for activity against five different human cancer cell lines [132]. Compounds **139** and **140** displayed the greatest potency, with IC_50_ values of 0.086/1.710 µM and 1.853/0.635 µM against BRAF^V600E^/HDAC1, respectively. Compounds **139** and **140** were further tested against RAF and HDAC isoforms, with compound **139** displaying IC_50_ values of 0.045, 0.134, and 0.102 µM against ARAF, ^wt^BRAF, and CRAF isotypes, respectively. Compound **140** was less active against the RAF isoforms, while both of these compounds were inactive against HDAC6 and HDAC8 at a concentration of 50 µM. Compound **139** also displayed antiproliferative activity against A549, SK-Mel-2, and MV4-11 cell lines, with IC_50_ values of 9.11, 5.40, and 0.38 µM, respectively. By comparison, the IC_50_ values determined for SAHA (vorinostat) and sorafenib against these three cell lines were 18.1, 8.20, 0.11 µM and 10.6, 9.30, 0.42 µM, respectively.

Yun et. al. reported a series of purine-based dual inhibitors of HDAC1/2 and CDK2 as potential multi-targeting anticancer agents and tested these compounds for activity against HCT116, HeLa, A375, HepG2, and H460 cancer cell lines [133]. The authors designed compounds by combining the aminobenzamide moiety common to one class of HDAC inhibitors with the purine-based pharmacophore present in CDK2 inhibitors. Of the target compounds, **141** was the most potent against four of the five cancer cell lines tested, displaying IC_50_ values ranging from 0.47–1.59 μM against these cell lines. Compound **141** also displayed IC_50_ values of 70.7, 23.1, and 800 nM against HDAC1, HDAC2, and CDK2, respectively. By comparison, the HDAC inhibitor CS055 displayed IC_50_ values of 260 and 66.5 nM against HDAC1 and HDAC2, respectively, while the CDK2 inhibitor roscovitine exhibited an IC_50_ value of 110 nM against this enzyme. Docking simulations with **141** suggested hydrogen bonding interactions and coordination with the zinc ion are important for binding of the aminobenzamide moiety of this compound to HDAC1 and HDAC2, while hydrogen bonds formed by the purine ring and the 1,2-diaminobenzene moiety promote binding of compound **141** to CDK2. Antitumor activity for **141** was evaluated in a HCT116 xenograft colon cancer model in nude mice. When compound **141** was administered to these mice at 25, 50, and 100 mg/kg by i.p. injection once daily for 21 days, tumor growth inhibition of 28%, 40%, and 44% was observed, respectively, compared to the control group.

### 4.7. Lysine-Specific Demethylase 1 (KDM1A) Inhibitors

**Figure 13:**
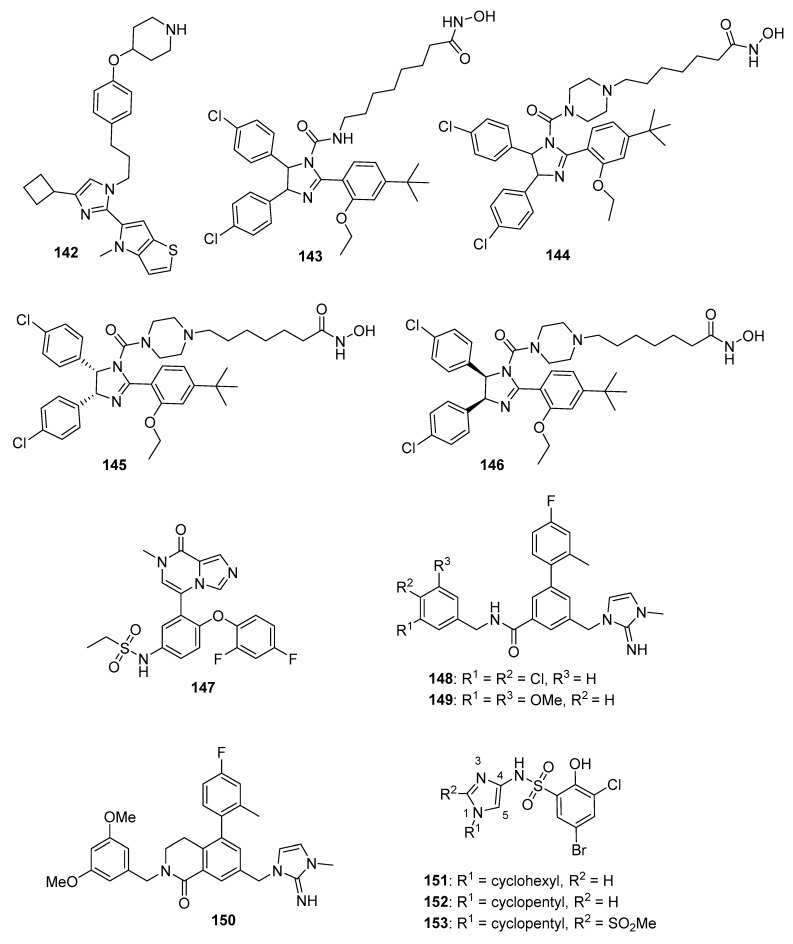
Imidazole-containing compounds reported as inhibitors of KDMA1, MDM2, BET, and WDR5 (compounds **142**–**153**).

Lysine-specific demethylase 1 (LSD1 or KDM1A) demethylates lysine residues (K4 and K9) of histone H3 protein and also regulates lysine methylation in other proteins. KDMA1 plays an important role in cancer cell growth and survival through the regulation of transcription, and the overexpression of KDMA1 has been associated with a range of cancers (reviewed in Reference [134]). KDMA1 has, thus, emerged as an anticancer target. New imidazolylthienopyrroles have been reported which act as reversible inhibitors of KDM1A [135]. Compound **142** (see Figure 13 for structures of compounds **142**–**153**) displayed an IC_50_ value of 0.10 nM against KDM1A. A dose-dependent increase in the expression of CD86, a biomarker related to KDMA1 inhibition, was also observed in THP-1 cells treated with **142** at concentrations of 5 and 50 nM. In addition, 38.8% and 72.3% inhibition of colony formation was observed when THP-1 cells were treated with compound **142** at concentrations of 50 and 500 nM. When compound **142** was given orally at a dose of 30 mg/kg/day, 5 days a week for 2 weeks in separate murine models of acute promyelocytic and acute myeloid leukemia, survival in these groups of mice was prolonged by 55% and 70%, respectively.

### 4.8. p53-Murine Double Minute 2 (MDM2) Inhibitors

The transcription factor p53 plays a critical ‘gate keeper’ role in cancer suppression, while the murine double minute 2 (MDM2) protein is the major inhibitor of p53 within the cell. Interaction between MDM2 and wild-type p53 is responsible for inhibition of p53 function via three separate mechanisms: (1) direct binding to p53 resulting in inhibition of p53 binding to target DNA, (2) nuclear export of p53, and (3) ubiquitination of p53 via MDM2′s E3 ligase activity. Several MDM2 inhibitors are in clinical trials, and the search for additional antagonists as anticancer candidates is in progress (see References [136,137] for reviews of MDM2 inhibition in cancer). He at al. designed and synthesized putative dual inhibitors of MDM2 and HDACs based on known substituted dihydroimidazole MDM2 inhibitors and HDAC inhibitors [138]. Compound **143** showed good activity toward both MDM2 (K_i_ = 0.34 μM) and HDAC1 (IC_50_ = 0.27 μM). To improve binding affinity and solubility, piperazine and piperidine moieties were incorporated into the aliphatic chain. Compound **144** exhibited balanced activity, displaying a K_i_ of 0.11 µM against MDM2 and an IC_50_ value of 0.82 µM against HDAC1. Testing of compounds for their antiproliferative activities against HCT116, MCF-7, A549, and NCI-H1299 cancer cell lines revealed the effect of compound **144** on the A549 cell line (IC_50_ = 0.91 μM) and the HCT116 cell line (IC_50_ = 1.08 μM). Compound **144** was selective for HDAC6 (IC_50_ = 17.5 nM) over HDAC1 (IC_50_ = 821 nM), HDAC2 (IC_50_ = 421 nM), HDAC3 (IC_50_ = 178 nM), and HDAC8 (1224 nM). Chiral separation of compound **144** gave rise to isomers **145** and **146**; the latter was more active. Compound **146** displayed 180 times greater potency towards MDM2 (K_i_ of **145** = 10.89 μM and **146** = 0.06 μM) and almost 10 times better antiproliferative activity against HCT116 (IC_50_ value of **145** = 10.03 μM and **146** = 1.11 μM) and A549 (IC_50_ value of **145** = 9.02 μM and **146** = 0.99 μM) cell lines compared to **145**. Similar activity was observed for the two isomers against HDAC1, however (IC_50_ value of **145** = 0.91 μM and **146** = 0.89 μM). In an A549 xenograft model in nude mice, compound **144** exhibited 65.4% tumor growth inhibition when given orally at 100 mg/kg/day for 21 days, while tumor growth inhibition values of 57.3% and 44.0% were observed for the known HDAC and MDM2 inhibitors SAHA and Nutlin-3, respectively, when given by the same route and dosing regimen.

### 4.9. Bromodomain and Extraterminal (BET) Protein Inhibitors

The BET protein family (BRD2, BRD3, BRD4, and BRDT) plays an important role in oncogene expression. These proteins have tandem bromodomains (BD1 and BD2) that regulate gene expression through recognition of acetylated histone lysine residues, and disruption of this association by BET inhibitors is a promising approach in cancer drug discovery and development (see Reference [139] for a review of bromodomains as a drug target). Novel derivatives of 7-methylimidazopyrazinone synthesized by Yang et al. were evaluated for their activity against BET proteins and for their activity against a lymphoblast-like cell line (Raji), a leukemia cell line (HL-60), and a human pancreatic (BxPC-3) cancer cell line [140]. Compound **147** displayed IC_50_ values of 33 nM against BRD4/BD1 and 25 nM against BRD4/BD2 in vitro. This compound also exhibited an IC_50_ value of 110 nM against the HL-60 cell line along with 43.2% inhibition of BxPC-3 cell proliferation at a concentration of 2 µM. In terms of the SAR for these molecules, replacement of the difluorophenyl substituent with a pyridyl group generally caused a significant decrease in potency. Western blotting analysis revealed that the treatment of BxPC-3 cells with compound **147** at concentrations of 5, 10, and 15 µM caused upregulation of the BRD4 protein, caspase 3, and caspase 9, as well as downregulation of c-Myc and Bcl-xl, suggesting that compound **147** modulates apoptosis in this cell line.

### 4.10. WD Repeat Domain 5 (WDR5) Inhibitors

WDR5 belongs to the family of WD40 repeat (WDR) domain proteins, a group of proteins containing a central pocket for peptide binding that is amenable to the design of small molecule ligands. WDR5 is known to play a role in the transcriptional activation of oncogenes by facilitating histone methylation, resulting in the proliferation of cancer cells, tumor growth, and metastasis (see Reference [141] for a review of WDR domain proteins and Reference [142] for a review of WDR5 in cancer). The transcription factor MYC, a key driver of many tumors, has also been shown to rely on its interaction with WDR5 to bind to chromatin [143]. WDR5 has, thus, emerged as an attractive target for cancer therapeutics.

Tian et al. synthesized new compounds based on dihydroimidazole imine **148** in which the structure-guided optimization of this compound led to improved WDR5 inhibitory activity, as well as anticancer activity [144]. Replacement of the 4-fluoro-2-methylpyridin-3-yl and 3,4-dichlorobenzyl moieties present in compound **148** with 4-fluoro-2-methylphenyl and 3,5-dimethoxy benzyl groups, respectively, resulted in compound **149**. This compound exhibited potent WDR5 binding with a K_i_ value of 0.049 nM, potent MLL1 histone methyltransferase inhibition (IC_50_: 3.4 nM), and antiproliferative activity with GI_50_ values of 15,000, 470, and 480 nM against K562 leukemia, MV4-11, and MOLM-13 cell lines (the former cell line is p53 null, while the latter two MLL-fusion cell lines express wild-type p53). These values were similar to those obtained for compound **148**. In general, substitutions at the 3,4 or 3,5 positions of the benzylamine group were optimal for WDR5 activity, as well as cellular inhibitory activity. Conformational restriction of the amide functionality occurring in compound **149** provided dihydroisoquinolinone containing compound **150**, which displayed enhanced potency (WDR5 K_i_ value of < 0.02 nM; MLL1 histone methyltransferase IC_50_ of 2.2 nM; GI_50_ values of 8000, 38, and 78 nM against K562, MV4-11, and MOLM-13 cell lines, respectively). Moreover, compound **150** displayed an improved K562:MV4-11 selectivity index of 210. Compound **150** also reduced the expression of the WDR5 bound genes *RPS24* and *RPL35* in MV4-11 cells treated with a 300 nM concentration of this compound for 3 days; however, the expression of genes not bound to WDR5 (*RPS11* and *RPL14*) was not reduced under these conditions. Moreover, treatment of MV4-11 cells with 600 nM compound **150** resulted in reduced MYC binding to chromatin at the *RPS24* and *RPL35* genes. Compound **150** displayed antiproliferative activity in MYC-driven cancer cells with GI_50_ values of 0.26, 0.49, 0.24, 1.2, 0.58, and 3.2 μM against CHP-134, Ramos, Raji, Daudi, and SW620 cell lines, respectively, although the MYC expressing human colon cancer cell line SW480 was resistant to treatment with compound **150**.

Applying NMR-based fragment screening of ^15^N-labeled WDR5 (consisting of amino acids 23–334), an imidazole sulfonamide (compound **151**) was designed and synthesized that bound to WDR5 and disrupted the interaction between this protein and a fluorescent probe with a K_d_ of 1.0 μM [145]. To optimize the binding of compound **151** to WDR5, various hydrophobic substitutions were made at the N1 position of imidazole, leading to the synthesis of compound **152**, which displayed a K_d_ value of 0.40 μM. Replacement of imidazole with its isosteres and bioisosteres, such as 1,2,4-triazole, pyrazole, thiazole, and benzothiazole, led to either a modest or significant decrease in binding affinity. Further optimization of compound **152** via modification at the C2 position of imidazole resulted in the discovery of compound **153**, which displayed a K_d_ value of 0.10 μM. While small substitutions, such as methyl, ethyl, ethyl ester, and methyl sulfone, at the C2 position in compound **152** resulted in molecules possessing K_d_ values in the range between 0.10–0.22 μM, placement of larger groups such as 4-substituted piperazine-amide and piperidine-amide led to a decrease in the K_d_. Six of the seven active compounds disrupted the interaction between WDR5 and MYC in HEK-293 cell lysates in a co-immunoprecipitation assay at a concentration of 50 μM, while a structurally related compound showing no measurable WDR5 affinity (K_d_ > 40 µM) did not disrupt the WDR5-MYC complex. Active compounds also inhibited the histone methyltransferase activity of MLL-1 when in complex with WDR5, RBBP5, ASH2L, and DPY30.

### 4.11. Signal Transducer and Activator of Transcription 3 (STAT3) Inhibitors

The transcription factor STAT3 is vital for cancer cell survival and proliferation. STAT3 is activated in many forms of cancer, resulting in the proliferation of cancer cells and, through the effect of STAT3 on its target genes, immunosuppression. Inhibition of STAT3 activity by small molecules can impede the growth of cancer cells, making STAT3 an important target in anticancer drug discovery (reviewed in Reference [146]).

**Figure 14:**
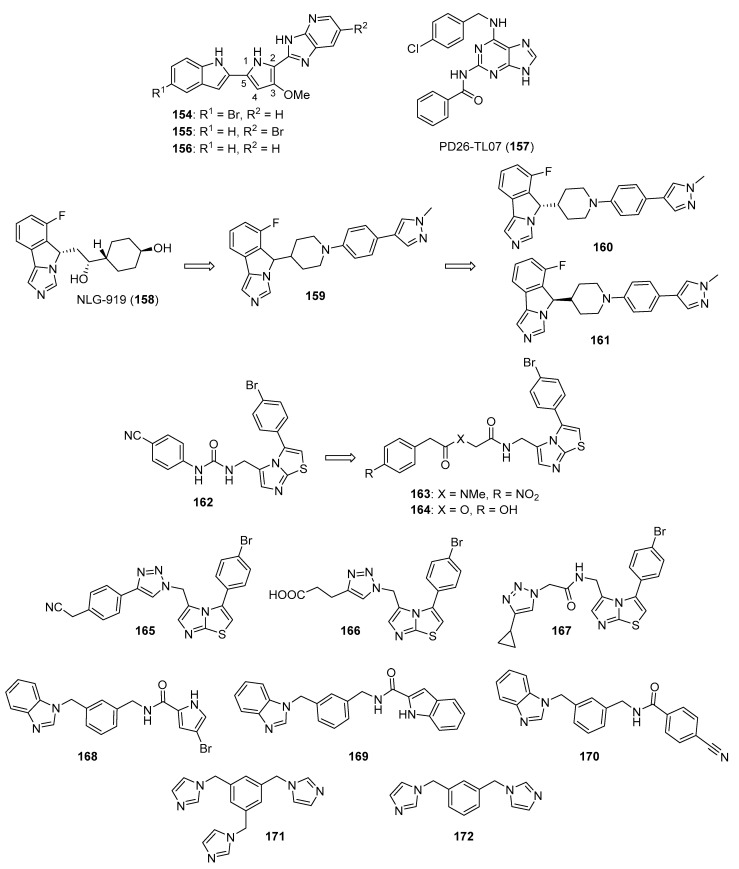
Imidazole-containing compounds reported as inhibitors of STAT3, IDO, and aromatase (compounds **154**–**172**).

A series of 2-imidazopyridines was synthesized and evaluated for activity against the PLC5 hepatoma cell line [147]. Of the synthesized compounds, **154**, **155**, and **156** (see Figure 14 for structures of compounds **154**–**172**) displayed activity against this cell line with IC_50_ values of 9.3 µM, 10.4 µM, and 11.7 µM, respectively. Antiproliferative activity is greater when the compounds contain the imidazopyridine ring system rather than benzimidazole at the C2 position of pyrrole. Mechanistic studies employing 10 µM concentrations of compounds **154**, **155**, and **156** revealed reduction of STAT3 phosphorylation in PLC5 cells. In addition, treatment of PLC5 cells with 10 µM concentrations of these compounds increased the activity of SHP-1, a phosphatase that dephosphorylates and, thus, deactivates STAT3, by approximately 2-fold compared to control. The expression of survivin, cyclin D1, and MCL-1, which are downstream targets of STAT3, were also decreased upon treatment of PLC5 cells with compound **155** at concentrations of 5 and 10 µM.

Wang et al. synthesized C2,C6-disubstituted purines as potential anticancer agents targeting STAT3 [148]. The target compounds were tested for their antiproliferative activity against HCT116, SW480, and MDA-MB-231 cancer cell lines, as well as for their effects, on a human breast epithelial cell line (MCF-10A). PD26-TL07 (**157**) displayed potent antiproliferative activity with IC_50_ values of 1.77, 1.51, and 1.25 μM against HCT116, SW480, and MDA-MB-231 cancer cells, respectively, while exhibiting low toxicity to MCF-10A cells (IC_50_ > 100 μM). IL6-induced phosphorylation of STAT3 was inhibited in MDA-MB-231 cells treated with 1–10 µM concentrations of compound **157** as assessed by Western blotting, but the same concentrations of this compound had no effect on IL6-induced STAT1 phosphorylation. In an MDA-MB-231 murine xenograft model, administration of compound **157** (30 mg/kg by i.p. injection) resulted in a significant decrease in tumor weight compared to animals receiving vehicle (TGI of 47.8%), while no difference in body weight between the two groups was observed.

### 4.12. Indoleamine-2,3-Dioxygenase (IDO)/Tryptophan 2,3-Dioxygenase (TDO) Signaling Inhibitors

IDO and TDO catalyze the oxidation of tryptophan to N-formylkynurenine, the first step of tryptophan breakdown. Accumulation of tryptophan metabolites results in immunosuppression through multiple mechanisms, and the overexpression of the predominant IDO isoform (IDO1) occurs in some cancers. Since the immune system plays an important role in limiting cancer cell growth, invasion, and metastasis, compounds that inhibit IDO and TDO are of interest as anticancer candidates. While there are currently no approved drugs that function as IDO modulators, candidates, such as epacadostat, indoximod, and GDC-0919, are in various phases of clinical trials, and the identification of novel IDO1 and TDO inhibitors remains of interest in anticancer drug discovery (see Reference [149] for a review of IDO in cancer and cancer therapy).

Tu et al. synthesized new imidazoisoindole derivatives based on **158** (NLG-919), a dual inhibitor of IDO and TDO [150]. Among these derivatives, compound **159** displayed IC_50_ values of 26 nM on IDO and 132 nM on TDO in target-based assays and 101 nM against the HeLa cell line in a phenotypic assay. In addition, compound **159** showed moderate inhibition of CYP3A4 (IC_50_ = 8.4 µM) and IC_50_ values > 10 µM against four other CYP450 isoforms. Chiral separation of compound **157** gave rise to enantiomers **160** and **161**. Among these, only compound **160** was active against IDO (IC_50_ = 9.7 nM) and TDO (IC_50_ = 47 nM); this compound also displayed an IC_50_ value of 76 nM against HeLa cells. A single 100 mg/kg oral dose of compound **160** administered to C57 mice caused a 57% reduction in kynurenine (a metabolite formed in tryptophan breakdown) at 2 h after administration. At a 50 mg/kg oral dose of compound **160** given in an MC38 murine xenograft model twice daily for 14 days, 78% tumor growth inhibition was observed. Moreover, the combination of compound **160** given by the same dosing regimen, together with the anti-PD-1 (programmed death 1) monoclonal antibody SHR-1210 (administered i.p. at 5 mg/kg qod ×8), displayed improved efficacy (TGI > 90%) in the MC38 xenograft model.

Griglio et al. synthesized a series of imidazothiazole derivatives based on compound **162** (IDO1 IC_50_ = 77 nM) [151], employing the Ugi and Passerini multicomponent reactions, and studied their inhibitory activities against IDO1 [152]. Some of these compounds displayed IC_50_ values below 1.0 μM against the recombinant human IDO1 (rhIDO1) enzyme; among those, **163** and **164** displayed enzymatic IC_50_ values of 0.81 and 0.20 μM, respectively. However, only compound **163** displayed moderate inhibition (24% at a concentration of 10 μM) in an IDO cellular inhibition assay in the human melanoma A375 cell line, while other compounds were inactive in this assay. The authors speculated that poor solubility, avid serum protein binding, or efflux from the target cells may have led to the disconnect between inhibition of rhIDO1 activity and activity in the cell-based assay. In terms of the SAR for these compounds against rhIDO1, the placement of benzyl groups at the “left” side of the target molecules generally resulted in better inhibitory activity compared to derivatives possessing phenyl substitutions. Serafini et al. further extended the work by Griglio, synthesizing two additional series of imidazothiazole derivatives by replacing the benzylamide/benzyl ester moiety in **163** and **164** with 1,4-disubstituted 1,2,3-triazole and 1,2,3-triazole amides [153]. Of the synthesized imidazothiazole-1,4-disubstituted 1,2,3-triazole derivatives, **165** and **166** displayed IC_50_ values of 0.5 and 1.1 μM against rhIDO1, respectively, while, among triazole amide derivatives, compound **167** displayed an IC_50_ value of 0.2 μM against this enzyme.

As a follow-up to these studies, Serafini et al. identified benzimidazoles as IDO1 inhibitors with the aid of a virtual screen of the IDO1 active site using the ZINC15 database [154]. Fifty of the top 500 molecules identified in this screen were purchased and evaluated in a cell-based assay measuring kynurenine levels in the A375 melanoma cancer cell line (which expresses high kynurenine levels when stimulated with INF-γ). Among these compounds, the hit molecule **168** displayed an IC_50_ value of 16 nM in this assay. Compound **168** was resynthesized and subsequently displayed a K_d_ value of 0.55 μM against recombinant human IDO1, while epacadostat displayed a K_d_ value of 3.46 μM against this enzyme. The authors then designed a new series of molecules based on compound **168**. The replacement of benzimidazole with indole led to a loss of activity, consistent with docking studies demonstrating a structural requirement for benzimidazole for IDO1 inhibition through heme binding at the active site. The replacement of the amide functionality with an ester group abolished activity, while replacement of pyrrole with furan and thiophene led to a decrease in potency. Of the derivatives synthesized, compounds **169** and **170** displayed potency comparable to compound **168** in the cellular IDO1 inhibition assay, with IC_50_ values of 72 and 90 nM, respectively. Further testing of compound **168** in a mastocytoma cell line expressing either murine TDO (P1.TDO) or IDO1 (P1.IDO1) showed that this compound displayed IC_50_ values of 5.46 μM against P1.TDO and 12.7 nM against P1.IDO1, with the assay being performed by measuring the secretion of L-kynurenine into the medium. Compounds **169** and **170** also displayed high selectivity for IDO1 over TDO in this assay. Compound **168** exhibited IC_50_ values ranging from 16–605 nM against six cancer cell lines, while compounds **169** and **170** displayed IC_50_ values ranging from 72–140 nM and 90–1940 nM on a subset of three of these cell lines. Pretreatment of immunosuppressive monocytes taken from pancreatic ductal adenocarcinoma patients with 30 µM concentrations of compounds **168** and **169** (a concentration that was not toxic to these monocytes) reduced the antiproliferative effect of the monocytes on CD3^+^ T cells.

### 4.13. Aromatase Inhibitors

Aromatase, a member of the CYP450 superfamily of enzymes, aids in the conversion of androgens to estrogen. The production of estrogen due to the action of the aromatase enzyme enhances tumor cell growth in hormone-dependent breast cancer, therefore aromatase inhibitors, such as letrozole, have been used clinically for the treatment of this type of cancer (see Reference [155] for a review of the use of aromatase inhibitors in breast cancer). Kalalinia et al. synthesized new azole derivatives as candidate aromatase inhibitors [156]. Neither the target compounds nor letrozole reached 50% inhibitory activity against either MCF-7 or HepG2 cancer cell lines at a concentration of 100 µM. However, compounds **171** and **172** efficiently inhibited aromatase in cultured cells using a commercial estrone ELISA with IC_50_ values of 0.2 nM and 6.8 nM, respectively, while letrozole displayed an IC_50_ value of 0.3 nM in this assay (the cell line used was not specified). Compound **171** decreased androstenedione-induced uterine hypertrophy in immature female rats when administered orally at doses of 10, 50, and 100 µg/kg once a day for four days, while letrozole (given orally at 10 µg/kg) produced a similar effect. In adult male rats treated with adrenocorticotropic hormone (ACTH, which stimulates cortisol and aldosterone production), a single oral dose of letrozole (4 mg/kg) significantly decreased serum concentrations of cortisol and aldosterone in comparison to control, while the same oral dose of compound **171** did not have significant effects on the serum concentration of these steroids, showing the specificity of compound **171**.

### 4.14. Inhibition of Aldehyde Dehydrogenase

**Figure 15:**
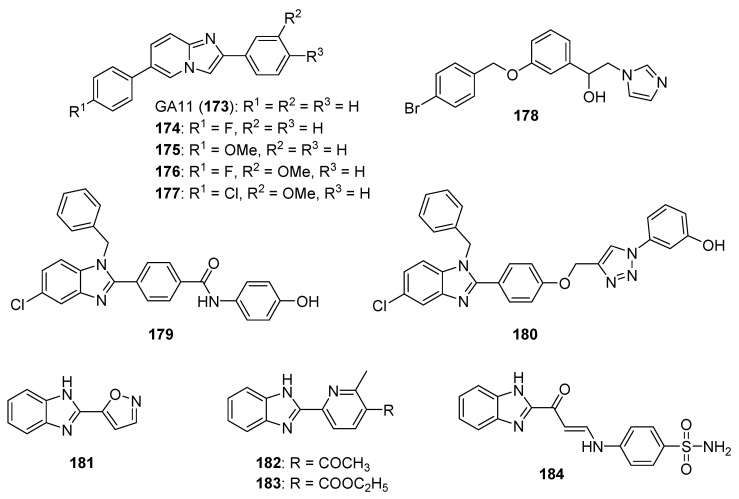
Imidazole-containing compounds reported as inhibitors or putative inhibitors of ALD1A3, HO-1, galectin-1, and GST (compounds **173**–**184**).

Aldehyde dehydrogenase subtype 1A3 (ALD1A3) is overexpressed in high mortality glioblastomas and plays an important role in glioma cell invasion [157]. The inhibition of this enzyme may, thus, provide an attractive strategy for the treatment of certain brain tumors. Toward this end, Quattrini et al. synthesized novel derivatives of imidazopyridine targeting ALD1A3 [158]. The authors crystallized preliminary hit GA11 (**173**) (see Figure 15 for structures of compounds **173**–**184**) with human recombinant ALDH1A3 to aid inhibitor design. Analogs **174**–**177** displayed selectivity towards ALDH1A3, with IC_50_ values of 22.8, 17.8, 21.2, and 6.4 μM, respectively, while displaying no significant inhibition of aldehyde dehydrogenase subtypes ALD1A1 and ALD1A2 when tested at concentrations of 25 μM. Of these compounds, **174** displayed the greatest potency against human patient-derived glioma specimens with IC_50_ values of 25.2 nM, 63.4 nM, and 2.58 pM against PN-157, MES-267, and MES-374 cells, respectively.

### 4.15. Heme Oxygenase-1 (HO-1) Inhibitors

HO-1 participates in the degradation of heme; overexpression of this enzyme is linked to protective effects on cancer cells and may promote cancer progression, cancer cell growth and survival, and metastasis (see Reference [159] for a review of HO-1 as an anticancer target). Ciaffaglione et al. synthesized a series of arylethanolimidazole-based derivatives as candidate HO-1 inhibitors [160]. Compound **178** displayed significant activity against HO-1, exhibiting an IC_50_ value of 0.90 µM. In terms of the SAR for these compounds, removal or oxidation of the hydroxyl group led to diminished activity against HO-1. Compound **178** exhibited moderate potency against the hormone-sensitive MCF-7 cell line (IC_50_ = 47.4 µM), while this compound was inactive against the hormone-resistant MDA-MB-231 cancer cell line.

### 4.16. Galectin-1 Inhibitors

Galectin-1 is a carbohydrate-binding protein that plays an important role in tumor invasion, progression, metastasis, and angiogenesis when overexpressed (reviewed in Reference [161]). Thus, galectin-1 inhibition is a potential anticancer strategy. Goud et al. synthesized 1-benzyl-benzimidazole derivatives as potential galectin-1 targeted anticancer agents and evaluated their activity against MDA-MB-231, A549, MCF-7, DU-145, HT29, and HCT116 cancer cell lines [162]. Compound **179** was as active as any of the target compounds against MCF-7 cells, exhibiting an IC_50_ value of 7.01 µM against this cell line. Treatment of MCF-7 cells with compound **179** (10–300 µM) caused a dose-dependent reduction of the expression of Gal-1 protein as determined by ELISA; the binding of compound **179** with the galectin-1 protein was confirmed by fluorescence spectroscopy and surface plasmon resonance experiments. In an extension of this work, benzimidazole-1,2,3-triazole hybrid compounds were synthesized as galectin-1 targeted agents based on compound **179** and were evaluated for their activity against NCI-H460, A549, MDA-MB-231, and MCF-7 cancer cell lines [163]. Target compound **180** displayed IC_50_ values of 0.99, 0.63, 0.94, and 1.3 μM against these four cell lines, respectively. Indicators of apoptosis were observed in A549 cells treated with compound **180** at 0.5–2.5 µM concentrations, including damage to the nucleus along with dose-dependent increases in sub-G_1_ cells and annexin V positive cells. Incubation of A549 cells in the presence of compound **180** at concentrations ranging from 10–300 µM resulted in a dose-dependent decrease in galectin-1 expression as determined by ELISA. Surface plasmon resonance and fluorescence spectroscopy studies indicated the binding of compound **180** to galectin-1.

### 4.17. Glutathione S-Transferase Inhibitors

Glutathione S-transferase (GST) is best known as a phase 2 metabolic enzyme involved in the detoxification of electrophiles. The non-hepatic GSTP1 isotype is abundantly expressed in some cancers and may be responsible for anticancer drug resistance by deactivating electrophilic drugs or by influencing cell signaling pathways (reviewed in Reference [164]). GSTP1 inhibitors may, thus, be useful for treating some forms of cancer, as well as for reversing resistance. Abd El-Karim et al. synthesized new benzimidazoles with the goal of inhibiting GST as an anticancer strategy [165]. Compounds **181** and **182** displayed IC_50_ values of 1.7 µg/mL and 0.67 µg/mL, respectively, against the GST enzyme prepared from human placenta, while the standard ethacrynic acid exhibited an IC_50_ value of 5.5 µg/mL. In general, placement of isoxazole or a 2-pyridyl group at the C2 position of benzimidazole resulted in greater potency against GST, while placement of pyrazole or N-methyl pyrazole at this position led to a decrease in potency. Compounds **183** and **184** displayed the greatest potency against the MCF-7 cell line (IC_50_ values of 3.2 and 2.7 μg/mL, respectively), while these compounds exhibited IC_50_ values of 3.7 and 4.9 μg/mL against the HCT cell line, respectively. These results indicate that compounds **183** and **184** may act on a target distinct from GST or that compounds **181** and **182** (IC_50_ values of 7.5 and 15.5 μg/mL against MCF-7 cells, respectively) may exhibit poor permeability to these cancer cell lines. Molecular docking studies revealed good binding between compounds **181** and **182** and GSTP1.

### 4.18. Lipoxygenase Inhibitors

**Figure 16:**
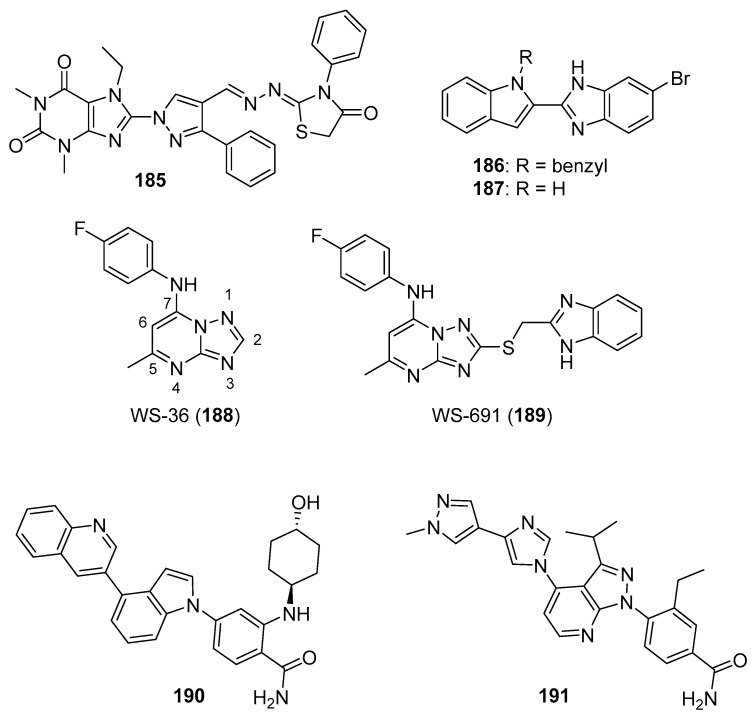
Imidazole-containing compounds reported as inhibitors of LOX-15, ER-α, ABCB1, and HSP (compounds **185**–**191**).

Lipoxygenases (LOXs) catalyze the conversion of polyunsaturated fatty acids into fatty acid hydroperoxides. With arachidonic acid as a substrate, different LOX isotypes can perform this oxidation at different sites of unsaturation. Since the 15-LOX isotype has been implicated in several forms of cancer, 15-LOX inhibitors are of interest as new anticancer candidates (see Reference [166] for a review of anticancer 15-LOX inhibitors). Afifi et al. described the synthesis and evaluation of purine-pyrazole hybrids as potential inhibitors of the 15-LOX isoform [167]. The target compounds exhibited IC_50_ values against 15-LOX in the range 1.76–6.12 μM. These compounds were also screened for in vitro activity against the MCF-7, PC-3, A549, HepG2, and Caco-2 cancer cell lines. Of the compounds tested, **185** was the most potent against all five cancer cell lines, exhibiting IC_50_ values ranging from 18.5–23.4 µM. While compound **185** (see Figure 16 for structures of compounds **185**–**191**) was one of the stronger 15-LOX inhibitors identified (IC_50_ = 2.33 µM), it displayed the best antioxidant activity in an assay measuring the reduction of 2,2′-diphenyl-1-picrylhydrazyl, displaying an IC_50_ value of 0.93 µg/mL (1.64 µM) in this assay. In molecular docking studies, compound **185** bound to 15-LOX (PDB ID code: 1-LOX) with a docking score of −3.37 kcal/mol.

### 4.19. Estrogen Receptor-α (ER-α) Inhibitors

As stated in Section 4.13, estrogen plays a vital role in mammary tumorigenesis. The effect of estrogen is mediated by estrogen receptors α and β (ER-α and ER-β); in most breast cancers, initiation and progression of tumors occurs by the activation of ER-α. Compounds, such as tamoxifen, which selectively bind to ER-α, thus, provide an important strategy for the treatment of breast cancer (see Reference [168] for a review of the use of ER-α antagonists in breast cancer). Singla et al. synthesized a series of indole-benzimidazole derivatives and examined their activity using the ER-α responsive T47D breast cancer cell line and an ER-α binding assay [169]. Target compounds **186** and **187** displayed IC_50_ values of 15.48 and 4.99 µM, respectively, against T47D cells; these molecules were more potent than the standard compound bazedoxifene (IC_50_ = 16.43 µM). Upon analysis of the structural features of these molecules, it was observed that bromoimidazole derivatives **186** and **187** were more potent than their unsubstituted imidazole analogs and significantly more potent than analogs containing an imidazopyridine ring system. Compounds **186** and **187** displayed affinity for ER-α, with IC_50_ values of 73.61 and 80.36 nM, respectively, in a competitive binding assay (the IC_50_ of bazedoxifene in this assay was 31.71 nM). Expression of ER-α mRNA and ER-α protein were both diminished by treatment of T47D cells with 15 µM and 5 µM concentrations of compounds **186** and **187**, respectively.

### 4.20. ABCB1 Inhibitors

Multidrug resistance (MDR) is a major cause of failure in cancer chemotherapy, as efflux of intracellular drugs by P-gp (the drug transporter MDR1 or ABCB1; overexpressed in multidrug resistant cancer cells) causes a decrease in the intracellular drug concentration (reviewed in Reference [170]). Thus, the modulation of ABCB1 activity has been explored as a strategy for cancer treatment. In this regard, Wang et al. designed new molecules based on WS-36 (**188**), which increases sensitization of the ABCB1-overexpressing SW620/Ad300 cell line to paclitaxel [171]. The combination of compound **188** (20 μM, a nontoxic concentration alone) with paclitaxel was toxic to SW620/Ad300 cells with an IC_50_ value of 2.34 μM, while paclitaxel alone displayed an IC_50_ value of 4.23 μM against this cell line. Various structural modifications at C8, C6, and C2 of the triazolopyrimidine core of compound **188** were carried out, resulting in the identification of compound WS-691 (**189**) as the most potent derivative. Paclitaxel displayed an IC_50_ value of 0.022 µM in combination with compound **189** (20 μM, a nontoxic concentration when given alone), while paclitaxel in combination with the first-generation ABCB1 modulator verapamil (4 μM) displayed an IC_50_ value of 0.30 μM. In terms of the SAR, placement of a thiomethyl group at the C2 position of the triazolopyrimidine core leads to higher potency compared to larger thioalkyl groups, while, among the various substituted anilines at the C7 position, only molecules substituted with 4-fluoroaniline (compound **189**) or aniline displayed potent MDR reversal activity. In studies employing the KB-3-1 cell line and its corresponding multidrug resistant cell line KB-C2 (which overexpresses ABCB1), a complete reversal of paclitaxel resistance was observed in KB-C2 cells at a 20 μM concentration of compound **189**. At concentrations of 10 and 20 μM, compound **189** increased the intracellular concentration of paclitaxel in ABCB1-overexpressing SW620/Ad300 cells compared to cells that were not exposed to this compound. Compound **189** did not inhibit the critical drug metabolizing enzyme CYP3A4 when tested at a concentration of 30 µM, in contrast with verapamil. In a SW620/Ad300 murine xenograft model, compound **189** (administered at 20 mg/kg/day for 21 days by intragastric injection) in combination with paclitaxel caused a reduction in tumor volume and tumor weight that was greater than the effect of paclitaxel alone.

### 4.21. Heat Shock Protein (HSP) Inhibitors

HSPs are molecular chaperones which play important roles in protein folding and also protect protein structure and function during cellular stress. Cancer cells depend heavily on HSPs for survival, thus making these chaperones important targets for cancer therapeutics. HSP90 has been the subject of most of these drug discovery and development efforts. While a number of HSP90 inhibitors are undergoing clinical trials, there are currently no approved anticancer drugs that act on this pathway, highlighting the need for further investigation of small molecules targeting HSPs (see References [172,173] for reviews focusing on the targeting of HSPs and HSP90, respectively). Uno et al. reported a series of molecules based on preliminary hit **190** as potential HSP90 inhibitors [174]. Compound **190** was a hybrid compound designed from the X-ray crystal structures of two previous inhibitors of HSP90. The authors explored the indole nucleus in compound **190** and also examined the effect of the 3-quinoline group. The former strategy revealed pyrazolopyridine as the optimal core, and the latter strategy showed that the best results were obtained through the replacement of quinoline with substituted imidazole. Further optimization showed that the best overall activity against HSP90α (IC_50_ = 69 nM) in conjunction with effectiveness against the SK-BR-3 cell line (IC_50_ = 330 nM), and good oral exposure in mice (AUC_0–6h_ = 90.1 µM·h at a dose of 50 mg/kg) was achieved when the C4 position of imidazole was substituted with N-methyl pyrazole and the C2 position of the phenyl ring attached to imidazopyridine was substituted with an ethyl group (compound **191**). After oral administration of compound **191** in an NCI-H1975 murine xenograft model at a dose of 10 mg/kg/day for 14 days, the tumor volume in control animals was approximately four times greater than in animals receiving compound **191**. Western blotting studies of tumor proteins from this in vivo study revealed that treatment with compound **191** resulted in decreased levels of the HSP90 client protein EGFR, thus inhibiting downstream phosphorylation of AKT, RPS6, and MAPK 1/3.

## 5. Anticancer Activities Shown by Imidazole Derivatives through Undefined Mechanisms

### 5.1. Benzimidazolium Salts

**Figure 17:**
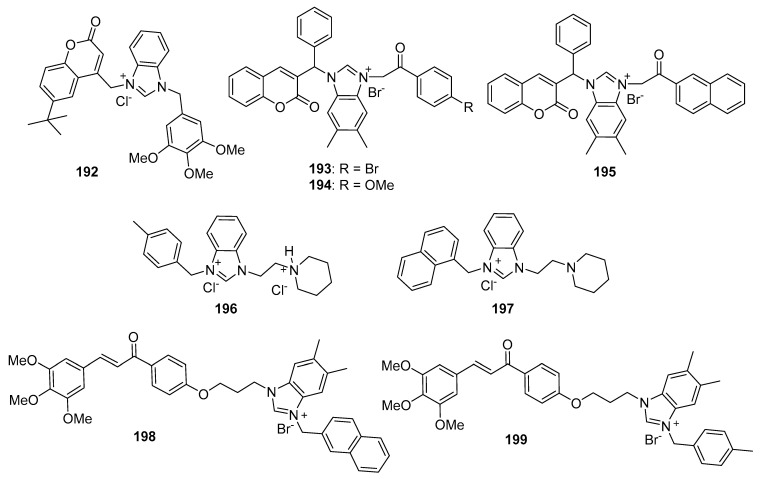
Benzimidazolium salts with anticancer activity (compounds **192**–**199**).

Karatas et al. synthesized coumarin substituted benzimidazolium salts and tested their cytotoxicities against PC-3 (prostate) and A2780 (ovarian) cancer cell lines [175]. Some of the synthesized compounds were found to have good cytotoxic activity in the PC-3 cancer cell line. PC-3 cells exposed to a 1 µM concentration of compound **192** (see Figure 17 for structures of compounds **192**–**199**) displayed a viability by 44.53%, while the viability of these cells exposed to 1 µM docetaxel was 31.45%.

Wang et al. synthesized a related series of 3-benzylcoumarin-imidazolium salts and evaluated their in vitro cytotoxicity against A549, HL-60, SMMC-7721, MCF-7, and SW480 cancer cell lines [176]. Compounds **193** and **194** exhibited potent activity on SW480 cells, displaying IC_50_ values of 0.20 and 0.36 µM, respectively, while compound **195** was active against all the cancer cell lines tested (IC_50_ values ranging from 2–5 µM). In terms of antiproliferative SAR, the activity pattern with respect to the central benzimidazole ring followed the general trend (starting with the most potent) 5,6-dimethyl benzimidazole > benzimidazole > 2-methyl benzimidazole > imidazole. In the compounds possessing 5,6-dimethyl benzimidazole and 2-methyl benzimidazole, compounds containing substitutions with phenacyl, 4-bromophenacyl, 4-trifluoromethylphenacyl, 4-methoxyphenacyl, 4-phenylphenacyl, and naphthylacyl generally displayed the most potent activities (IC_50_ values < 10 µM). Compound **195** caused apoptosis in the SMMC-7721 cell line at concentrations of 4 µM and 8 µM.

A series of piperidinylethyl-benzoimidazolium derivatives was synthesized by Akkoç et al. and was examined for cytotoxicity on Beas-2B, HEK-293T, DLD-1, HepG2, and MDA-MB-231 cancer cell lines [177]. Compound **196** displayed IC_50_ values of 17.80 µM and 15.16 µM on DLD-1 and HepG2 cell lines, respectively, while compound **197** exhibited IC_50_ values of 15.56 and 10.45 µM on DLD-1 and Beas-2B cell lines, respectively. The remaining compounds displayed IC_50_ values > 20 µM on all the cell lines tested.

Yang et al. designed, synthesized, and tested the anticancer activity of chalcone-benzimidazolium salts on five different human cancer cell lines [178]. Chalcone-imidazoles showed activity in the range 1.08–5.87 μM; however, their imidazolium salts proved to be more potent. The hybrid imidazolium salt **198**, possessing a naphthyl methyl substitution, and **199**, bearing a 4-methyl benzyl substitution, displayed the most potent IC_50_ values of 0.83 µM and 0.59 µM against HL-60 cell lines. Compound **198** exhibited IC_50_ values of 1.57 and 2.92 µM against MCF-7 and SW480 cell lines and was 11.1-fold and 5.8-fold more potent than cisplatin in the MCF-7 and SW-480 cell lines, respectively. Regarding substitution on the benzimidazole ring system, the order of activity was 5,6-dimethyl > 2-methyl > unsubstituted, while the substitutions on nitrogen, in general, followed the order napthyl > benzyl > phenacyl from most potent to least potent. Compound **198** also induced accumulation of SMMC-7721 cells in the G_0_/G_1_ phase at 2.5 and 5.0 µM concentrations and dose-dependent apoptosis in these cells at 5.0 and 10.0 µM concentrations.

### 5.2. Benzimidazole Containing and Related Molecules

**Figure 18:**
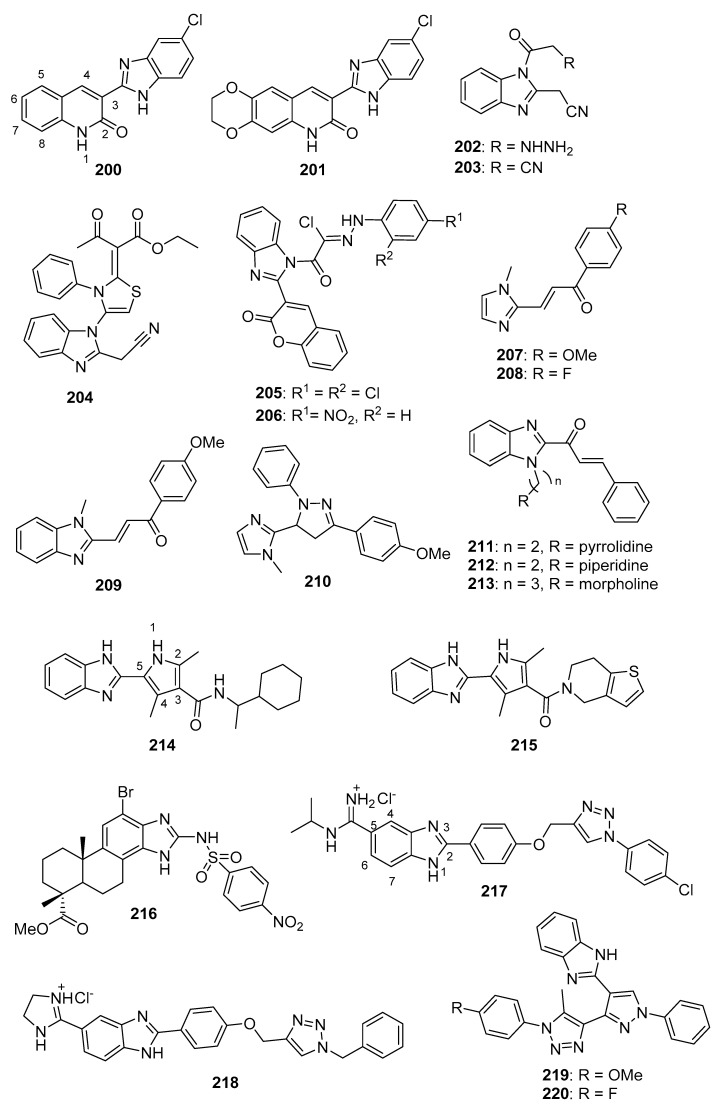
Benzimidazole containing and related molecules with anticancer activity (compounds **200**–**220**).

The in vitro antitumor potency of a new series of benzimidazole-quinolinone derivatives was evaluated against HepG2, SKOV3, NCI-H460, and BEL-7404 cancer cell lines along with the toxicity of these molecules against the HL-7702 normal human liver cell line [179]. Compounds **200** and **201** (see Figure 18 for structures of compounds **200**–**220**) exhibited the highest potency on HepG2 and BEL-7404 cell lines, with IC_50_ values of 8.45 µM and 9.06 µM, respectively. In terms of the SAR, the placement of an ethylenedioxy substitution at the 6,7 position of quinoline led to greater anticancer activity, while halide substitution on the benzimidazole portion increased activity compared to unsubstituted compounds or compounds with a methyl substitution. Treatment of HepG2 cells with **200** or **201** caused activation of p53 protein, caspase-3 and caspase-9, up-regulation of Bax/downregulation of Bcl-2, cleavage of PARP, and inhibition of CDK activity. In a murine NCI-460 xenograft model, compound **200** (50 mg/kg twice a day given by i.p. injection for 13 days) resulted in 66.9% reduction in tumor weight, while the standard drug cisplatin (2 mg/kg for 2 days by i.p. injection) reduced tumor weight by 63.2% in the same model. Administration of compound **201** (50 mg/kg given by i.p. injection for 13 days) resulted in 44.9% reduction in tumor weight in a BEL-7402 murine xenograft model, while administration of 5-FU (25 mg/kg for 2 days by i.p. injection) led to 34.5% tumor weight reduction in this model.

Novel N1, C2-substituted benzimidazoles were synthesized by Mohareb et al. and screened against A549, H460, HT29, MKN-45, U87MG, and SMMC-7721 cancer cell lines [180]. The most potent compounds in this series exhibited IC_50_ values in the range between 0.2–4.0 µM against all cancer cell lines tested, while compounds **202**–**206** were non-toxic when tested against shrimp larvae. In terms of the SAR, compounds containing hydrazine (**202**) or nitrile (**203**) at the R group possessed enhanced activity compared to the corresponding chlorine-containing derivative. The presence of -COCH_3_ and -COOC_2_H_5_ in compound **204** resulted in increased antiproliferative effects, while, among coumarin compounds, placement of 2,4-dichloro and 4-nitro substitutions on the phenyl substituent resulted in high anticancer activity on all the cancer cell lines tested.

Chouiter et al. synthesized chalcones and pyrazoline-type compounds based on an imidazole or benzimidazole core and evaluated the anticancer activity of these compounds against A549 adenocarcinoma and AGS stomach cancer cell lines; counter-screening was also performed against the MRC-5 human lung fibroblast cell line [181]. The target compounds were more potent against AGS cells. Compounds **207**, **208**, and **209** displayed IC_50_ values of 20.9, 29.3, and 15.1 µM against the AGS cell line and IC_50_ values of 67.1, >100, and 45.9 µM against the MRC-5 cell line. Compounds containing pyrazoline in place of the α, β-unsaturated ketone linker (such as compound **210**) were inactive on all the three cancer cell lines tested. Increased caspase-3 activity was observed in A549 and AGS cells exposed to compounds **207**–**209**.

A series of benzimidazole-chalcone hybrids synthesized by Hsieh et al. were screened for anticancer activity against A549, MCF-7, HepG2, and OVCAR-3 cancer cell lines [182]. Compounds **211**–**213** were active on all four cell lines tested, with IC_50_ values < 15 µM. Among these, compound **213** displayed the greatest antiproliferative activity with IC_50_ values of 10.93, 9.73, 8.91, and 10.76 μM against HepG2, A549, MCF-7, and OVCAR-3 cells, respectively. Analysis of the SAR indicated that the presence of 5- or 6-membered nitrogen-containing heterocycles linked at the *N*1 position of benzimidazole by an alkyl chain increased potency.

Suk et al. evaluated the activity of benzimidazole derivative **211** against sorafenib resistant HCC cell lines (Hep3B-SR and HuH7-SR) [183]. Compound **211** displayed significant antiproliferative effects against these two cell lines at a concentration of 25 µM. Western blotting studies revealed that, at a concentration of 15 µM, compound **211** inhibited p70S6, AKT, and STAT3 phosphorylation in these cells. The treatment of these cells with 15 µM compound **211** resulted in enhanced cleavage of PARP, as well as decreased expression of Fas, at the cell surface. When compound **211** was given at 4 mg/kg/day by i.p. injection, moderate tumor growth inhibition was observed in a sorafenib resistant murine xenograft model of HCC. Immunostaining revealed that mice treated with compound **211** displayed lower nuclear levels of Ki-67 and PCNA compared to control mice. Moreover, sorafenib resistant tumors from mice treated with compound **211** displayed lower levels of cyclin D1 and HCC biomarkers compared to tumors from mice that received vehicle.

Rasal et al. synthesized compounds containing a dimethylpyrrole carboxamide-benzimidazole scaffold as potential anticancer agents that were evaluated against the NCI 60 cancer cell line panel [184]. When tested at a concentration of 10 μM, compound **214** displayed the highest growth inhibition of 62.46% against the MDA-MB-435 and 40.24% against the MDA-MB-468 cell lines. In terms of the SAR, compounds bearing acyclic aliphatic amines in an amide linkage at the C3 position of pyrrole lacked potent anticancer activity. Introduction of imidazole or pyrazole at this position did not improve activity, while insertion of tetrahydrothienopyridine (compound **215**) has a positive influence on activity against T47D and MDA-MB-468 breast cancer cell lines.

The cytotoxic and apoptosis-inducing activity of benzimidazole derivatives of dehydroabietic acid (**100**; see Figure 9) were evaluated against MCF-7, HeLa, and HepG2 cancer cell lines and a normal hepatocyte (LO2) cell line by Li et al. [185]. Among these compounds, **216** displayed the highest activity with IC_50_ values of 0.87, 9.39, and 8.31 against MCF-7, HeLa, and HepG2 cell lines, respectively. This compound also displayed an IC_50_ value of 42.83 μM on the LO2 cell line, thus showing selectivity towards the cancer cell lines. The SAR indicated that compounds bearing electron-withdrawing groups on the phenylsulfonamide portion displayed stronger activity when compared to electron-donating groups. Moreover, sulfonamide derivatives displayed higher activities compared to amide derivatives. Incubation of MCF-7 cells with 1–4 µM concentrations of compound **216** caused a dose-dependent accumulation of cells in the S-phase of the cell cycle, as well as a dose-dependent increase in apoptotic cells.

Novel amidino substituted benzimidazoles linked to 1,2,3-triazole via a phenoxy methyl or ethyl linker were synthesized using microwave and ultrasound irradiation by Bistrovic et al. [186]. The antiproliferative activities of these compounds were evaluated against A549, CFPAC-1, HeLa, and SW620 cell lines, as well as on normal human lung fibroblasts (WI38). These compounds were highly selective towards the A549 cell line, with compounds **217** and **218** exhibiting high potency (IC_50_ values of 0.05 and 0.07 µM, respectively) and comparatively little effect on W138 fibroblasts (IC_50_ values of 8.04 and 6.89 µM, respectively). In terms of the SAR, compounds containing isopropyl amidine and imidazoline at the C5-position of benzimidazole were more active and selective towards the A549 cell line, while compounds possessing a phenoxymethyl group at the C2 position of benzimidazole also displayed potent activity against this cell line. A significant reduction in PDE5, CDK9/cyclin T1, TGM2, phospho SK1, phospho p38 MAPK, and phospho-p53 levels were observed in A549 cells treated with a 0.14-µM concentration of compound **218**. Compound **217** at a concentration of 0.10 µM did not affect PDE5, CDK9/cyclin T1, TGM2, or phospho-p53 levels but displayed similar (albeit weaker) effects on phospho SK1 and phospho p38 MAPK levels in A549 cells.

Ashok et al. prepared 1,2,3-triazole-based pyrazole-benzimidazole derivatives through conventional, as well as microwave-assisted, synthesis and evaluated their in vitro antiproliferative activity against C6 and MCF-7 cell lines [187]. These derivatives were obtained in higher yields using microwave-assisted synthesis (77–89%) compared to conventional heating in DMF (57–66%). Compound **219** displayed an IC_50_ value of 0.102 μM against the C6 cell line, while the standard drug cisplatin exhibited an IC_50_ value of 0.122 μM. Compounds **219** and **220** displayed IC_50_ values of 0.110 and 0.144 μM against the MCF-7 cell line, while the IC_50_ value for cisplatin against these cells was 0.596 μM.

### 5.3. Imidazopyridines, Imidazopyrimidines, and Related Compounds

**Figure 19:**
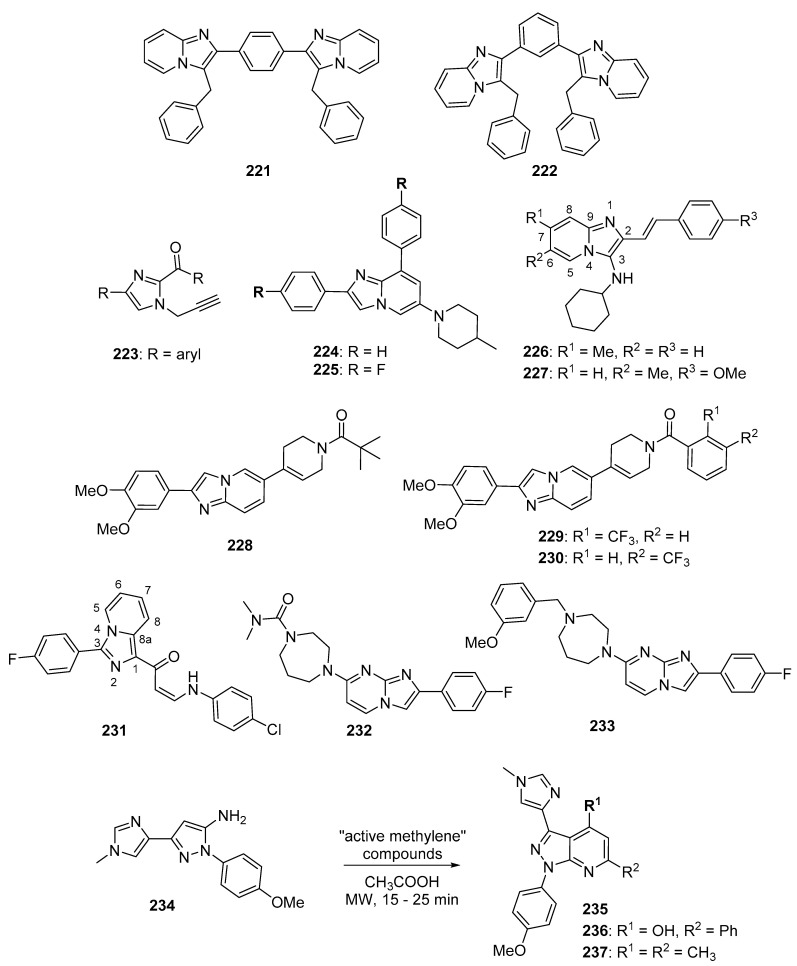
Imidazopyridines, imidazopyrimidines, and related compounds with anticancer activity (compounds **221**, **222**, **224**–**233**, **236**, and **237**). Reagents/reagent scaffolds (**223**, **234**) and target compound scaffold **235** from reactions described in the text are also shown.

A series of imidazole and imidazopyridine dimers synthesized by Meenakshisundaram et al. was tested for cytotoxicity against HeLa, MDA-MB-231, and ACHN cancer cell lines [188]. Compounds **221** and **222** (see Figure 19 for structures **221**–**237**) displayed GI_50_ values of 0.43 µM and 0.30 µM, respectively, against the MDA-MB-231 cell line, showing higher potency than the standard drug adriamycin (GI_50_ = 0.51 µM). In addition, administration of **221** and **222** (both given at 50 mg/kg for 14 days by gastric intubation) in a 7,12-dimethylbenz[*a*]anthracene-induced rat mammary carcinoma model caused a reduction of cancer markers (carcinoembryonic antigen and cancer antigen 15–3) and resulted in lower levels of the liver enzymes aspartate aminotransferase, alanine aminotransferase, and alkaline phosphatase compared to rats in the mammary carcinoma control group. In terms of the SAR for these compounds, imidazopyridine dimers were more active compared to imidazole dimers.

Güçlü et al. developed a novel method for the synthesis of imidazopyridine derivatives by reacting 4-substituted-*N*-propargyl biaryl ketones (scaffold **223**) with 4-methylpiperidine under microwave irradiation [189]. The derivatives were screened for activity against the LN-405 glioblastoma cell line and normal skin fibroblasts (WS-1). Compound **224** was the most potent analog against the LN-405 cell line, displaying an IC_50_ value of 10 µM, while compound **225** exhibited an IC_50_ value of 75 µM against this cell line. Compound **224** was selectively cytotoxic for the LN-405 cell line compared to the fibroblast cells (IC_50_ = 480 µM). Results from a comet assay in LN-405 cells treated with a 10 µM compound **224** showed that this derivative caused DNA damage (affecting 14% of the cells) compared to the control (where DNA damage was observed in 0.7% of the cells). In silico studies using Schrödinger suggested that the target compounds are likely to be orally available and should penetrate the blood-brain barrier.

Khalili et al. synthesized a library of styrylimidazopyridines and evaluated them for activity against three breast cancer cell lines [190]. Compound **226** displayed IC_50_ values of 9.59, 12.12, and 10.10 μM against MDA-MB-231, MCF-7, and T47D cell lines, respectively, while compound **227** displayed IC_50_ values of 8.13 and 9.61 μM against MCF-7 and T47D cell lines. In general, compounds synthesized from cyclohexyl isocyanides had higher potencies than derivatives synthesized from *tert*-butyl isocyanides. The placement of a methyl group at positions 5, 6, or 7 of the imidazopyridine core increased the anticancer activity of compounds obtained from cyclohexyl isocyanides, while insertion of chlorine at C6 caused the loss of activity against all three cell lines.

Chitti et al. reported the synthesis of imidazopyridine analogs as potential antiproliferative agents against A549, HeLa, and B16F10 cancer cell lines and also performed counter-screening using the HEK-293 cell line [191]. A subset of the synthesized compounds displayed antiproliferative activity in the range between 2.55–20.0 μM against the tested cancer cell lines, with compounds **228**–**230** exhibiting IC_50_ values in the range between 2.55–8.11 μM against these cancer cell lines. Among these, **229** and **230** were the most potent against the A549 cell line, displaying IC_50_ values of 2.55 and 3.69 μM against this cell line, respectively. In terms of the SAR, imidazopyridines possessing amide functionalities were more potent compared to derivatives containing a sulfonamide moiety.

A series of imidazopyridine-arylaminopropenone conjugates synthesized by Mani et al. were tested for activity against A549, HCT116, B16F10, BT474, and MDA-MB-231 cancer cell lines [192]. Among these compounds, **231** displayed the greatest potency against the HCT116 cell line (IC_50_ = 1.21 μM). While the structure-activity relationship is complex, the inclusion of an electron withdrawing group on the C3 phenyl group and on the terminal aniline generally resulted in increased potency. Compound **231** caused a dose-dependent increase in the percentage of HCT116 cells in the G_0_/G_1_ phase of the cell cycle at concentrations of 0.5–2.5 µM and increased the formation of reactive oxygen species in these cells at 1.0 and 2.5 µM concentrations.

Mantipally et al. synthesized a series of novel homopiperazine linked imidazopyrimidine derivatives as candidate cytotoxic and antimicrobial agents [193]. Evaluation of these compounds against a panel of four human cancer cell lines (SKOV3, DU-145, HeLa, and A549) revealed that compound **232** displayed an IC_50_ value of 5.98 µM against A549 cells, while compound **233** exhibited IC_50_ values of 6.24 µM, 6.54 µM, and 4.14 µM on DU-145, HeLa, and A549 cell lines, respectively. Compounds bearing an amide group on the diazepane nitrogen were generally more active when compared to analogs bearing a sulfonamide moiety at this position.

El-Borai et al. synthesized imidazolyl pyrazolopyridine derivatives (scaffold **235**) from 1-(4-methoxyphenyl)-3-(1-methyl-1*H*-imidazol-4-yl)-1*H*-pyrazol-5-amine (**234**) and “active methylene” compounds, such as ethyl benzoylacetate and pentane-2,4-dione, using microwave irradiation [194]. Comparing conventional heating to microwave irradiation, the microwave reaction occurred in high yields (81–92%) in just 15–25 min, while yields were lower (65–76%) and reaction times were longer (4–8 h) using conventional heating. Target compounds were tested for antiproliferative activity against the MCF-7 cancer cell line, with IC_50_ values in a narrow range (16.6–19.3 μg/mL). Compounds **236** and **237** displayed IC_50_ values of 16.6 and 18.1 μg/mL, respectively.

### 5.4. Naphthoquinone Imidazoles

**Figure 20:**
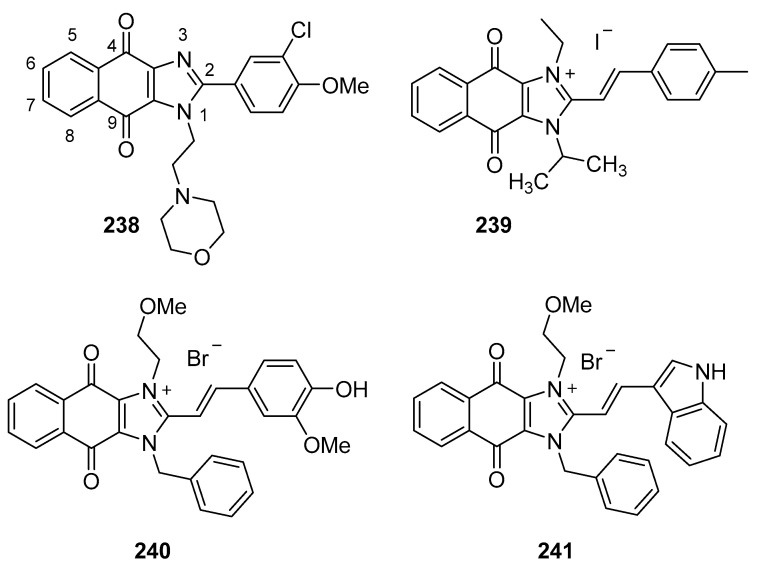
Naphthoquinone imidazoles with anticancer activity (compounds **238**–**241**).

Liu et al. synthesized morpholino ethyl-substituted naphthoquinone imidazoles that were tested for in vitro activity against MCF-7, HeLa, and A549 cell lines, as well as for their effects on the L929 normal mouse fibroblast cell line [195]. Compound **238** (see Figure 20 for structures of compounds **238**–**241**) displayed IC_50_ values of 4.3, 10.6, and 8.3 μM against A549, MCF-7, and HeLa cells, respectively, while exhibiting an IC_50_ value of 67.3 μM against the L929 cell line. In terms of the SAR, 2-alkyl-substituted compounds were less potent compared to 2-aryl-substituted compounds. In addition, compounds lacking a quinone moiety were inactive on the cancer cell lines tested.

Wei et al. synthesized a novel series of arylvinyl-naphthoimidazolium iodide and bromide derivatives and screened these compounds for antiproliferative activity against PC-3, HeLa, and A375 cell lines [196]. Compounds **239** and **240** displayed IC_50_ values of 0.022, 1.12, 1.07 µM and 0.022, 1.13, and 0.536 µM against PC-3, HeLa, and A375 cells, respectively, while indole-substituted compound **241** displayed IC_50_ values of 0.128, 0.059, and 0.212 µM against these cell lines, respectively. In general, phenyl vinyl substitutions at the C2 position of imidazole resulted in compounds that were more active against PC-3 cells; however, compounds bearing indole vinyl substituents were more active towards A375 and HeLa cell lines. In A375 cells, both compounds **239** and **241** decreased the expression of survivin and Bcl-2 and increased the percentage of annexin V positive cells at 1 µM concentrations. In a subcutaneous solid tumor model using Ehrlich’s ascites carcinoma cells, intraperitoneal administration of compound **241** at a dose of 0.6 mg/kg/day for 10 consecutive days resulted in 53.12% reduction in tumor progression compared to the vehicle control group.

### 5.5. Polysubstituted Imidazole Derivatives

**Figure 21:**
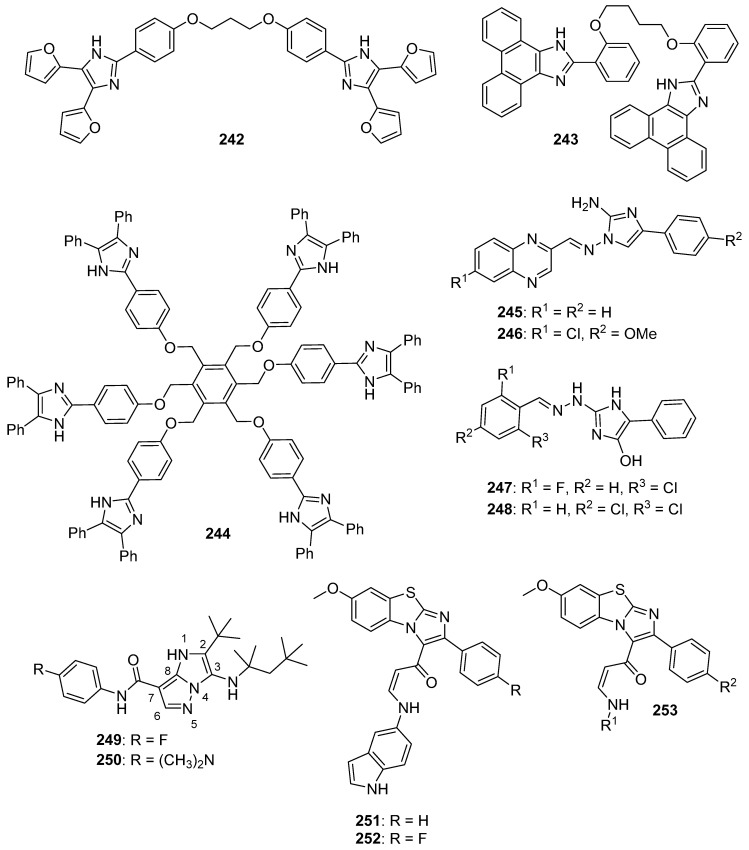
Polysubstituted imidazole derivatives with anticancer activity (compounds **242**–**253**).

Hebishy et al. reported the preparation of bis- and poly(imidazoles) via a three-component reaction among 1,2-diketones, aldehydes, and ammonium acetate using 10 mol% of a ZnO nanocatalyst under conventional heating, as well as microwave irradiation [197]. The target compounds were tested against HepG2, MCF-7, and Caco-2 cancer cell lines and were counter-screened using Vero (African green monkey kidney) cells. Among these target compounds, **242**, **243**, and **244** (see Figure 21 for structures of compounds **242**–**253**) exhibited IC_50_ values against HepG2 cells of 8.14, 9.58, and 9.77 µM, respectively, while all other compounds displayed IC_50_ values greater than 10 µM. Compounds **242** and **244** displayed selectivity indexes of 27.47 and 13.62 against HepG2 cells compared to Vero cells.

Ghanbarimasir et al. synthesized and evaluated the activity of 2-aminoimidazole-quinoxaline hybrids against MCF-7 and HCT116 cancer cell lines, along with their effects on a normal HFF cell line [198]. The most potent activity was exhibited by compounds **245** and **246**. Compound **245** displayed 83.3% inhibition against MCF-7 cells and compound **246** exhibited 77.3% growth inhibition against HCT116 cells when tested at a concentration of 50 μg/mL. The standard drug doxorubicin exhibited 93.3% and 97.2% inhibition against these cell lines, respectively, when tested at the same concentration. Unsubstituted quinoxaline compound **245** displayed the best activity; substitution of the phenyl group linked to the imidazole ring in compound **245** with electron-withdrawing or electron-releasing groups led to a decrease in potency. Among the 6-Cl and 7-Cl-quinoxaline series, the 4-methoxyphenyl substitution in the former series showed the highest potency against both cancer cell lines tested.

The anticancer activity of imidazole derivatives prepared from guanylhydrazone and phenylglyoxal was evaluated in vitro against MCF-7 and HepG2 cancer cell lines by Yavuz et al. [199]. The synthesized compounds displayed selectivity towards MCF-7 cells compared to HepG2 cells. The most potent compounds in this series (**247** and **248**) exhibited IC_50_ values against MCF-7 cells of 10.22 and 14.80 µM, respectively. Compounds substituted with electron-withdrawing groups as in **247** and **248** were more potent than compounds bearing electron-releasing groups.

Demjén et al. synthesized a library of imidazopyrazole-7-carboxamides and measured their activity against 4T1, MCF-7, and HL-60 cancer cell lines [200]. Compounds **249** and **250** displayed the greatest potency against the HL-60 cancer cell line, with IC_50_ values of 0.183 and 0.297 μM, respectively. Regarding substitution on the imidazopyrazole system, compounds having alkyl substitutions at C2 and alkylamines at C3 displayed the highest potency.

Shaik et al. reported the synthesis of benzoimidazothiazole-propenone conjugates as potential antiproliferative agents against HeLa, HepG2, A549, and MCF-7 cancer cell lines [201]. Compounds **251** and **252** displayed IC_50_ values ranging from 1.6–8.4 μM against the four cancer cell lines tested. Compound **251** showed the greatest potency against the HeLa cell line with an IC_50_ value of 1.6 μM. In terms of the SAR for these molecules, compounds possessing heteroaryl groups at R^1^ in scaffold **253** displayed superior potency compared to compounds possessing aryl rings at this position, while compounds displaying a chlorine at R^2^ generally exhibited inferior potency compared to compounds with a hydrogen atom at this position. Treatment of HeLa cells with compound **251** at a concentration of 5 μM caused a 40% decrease in the mitochondrial membrane potential and a 4-fold increase in the level of reactive oxygen species.

### 5.6. Natural Product Imidazole Derivatives

**Figure 22:**
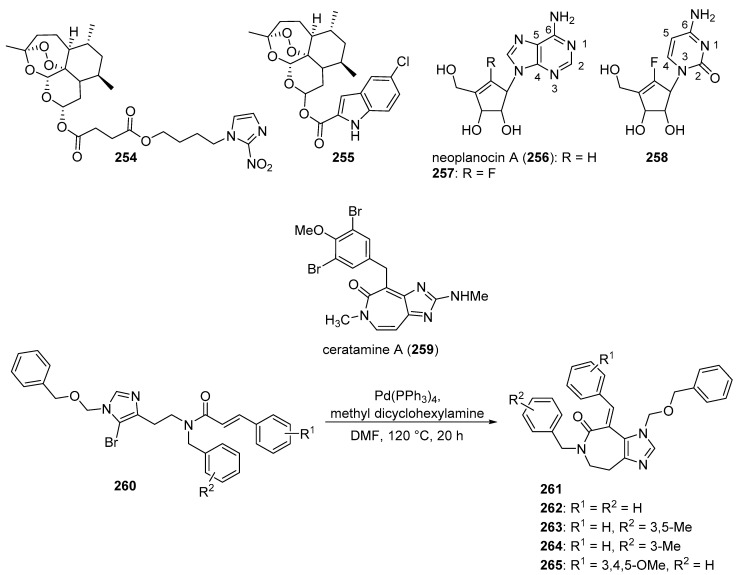
Natural product imidazole derivatives displaying anticancer activity (compounds **254**–**259** and **262**–**265**). Reagent scaffold **260** and target compound scaffold **261** are also shown.

Hu et al. synthesized a series of artemisinin-imidazole and artemisinin-indole hybrids which were tested in vitro against A549, MCF-7, HepG2, and MDA-MB-231 cancer cell lines and were counter-screened against a normal LO2 cell line [202]. Most of the artemisinin-imidazole and artemisinin-indole hybrids showed good potency (IC_50_ values below 20 μM) on MCF-7 and A549 cell lines when compared to artemisinin and dihydroartimisinin (IC_50_ values ranging from 34–51 μM against these cell lines). Artemisinin-imidazole hybrid compound **254** (see Figure 22 for structures **254**–**265**) exhibited an IC_50_ value of 9.78 μM against MCF-7 cells, while artemisinin-indole hybrid **255** displayed IC_50_ values of 5.25 and 6.17 μM on MCF-7 and A549 cell lines, respectively. In general, derivatives bearing an indole substitution instead of imidazole displayed slightly greater potency. Compound **255** showed outstanding reversal activity in MCF-7/ADR cells resistant to adriamycin, displaying a reversal fold of 117.

Based on the strong antitumor activity of the natural product neoplanocin A (**256**) and the related cyclopentenyl nucleoside **258**, a series of fluorocyclopentenyl pyrimidines and purines were synthesized and tested against six cancer cell lines [203]. Among the pyrimidine derivatives, **258** was the most active against these cell lines (A549, HCT-116, SNU-638, MDA-MB-231, SK-Hep-1, and PC-3), with IC_50_ values ranging from 0.18–0.76 µM, while the addition of a fluorine atom at the 5-position of the pyrimidine ring resulted in a significant loss of potency. For the purines, fluorocyclopentenyl analog **257** (IC_50_ values 1.10–2.17 µM) and its N6-methyl derivative (IC_50_ values 2.14–15.3 µM) displayed potency against the six cancer cell lines similar to or slightly lower than neoplanocin A (IC_50_ values 0.82–2.36 µM), but the corresponding derivative bearing an oxygen atom bound at C6 lacked activity. Addition of the bulkier cyclopropylmethyl group to the N6 amino group resulted in a loss of activity, as did addition of an amino group to C2 of the purine ring. The phosphoamidate prodrug of compound **257** was 1.6- to 17.7-fold less active than **257** itself against the cancer cell lines, leading the authors to speculate that anticancer action is through inhibition of S-adenosylhomocysteine hydrolase by **257** rather than interference with DNA or RNA polymerase by the triphosphorylated metabolite of **257**.

A novel series of imidazoazepine derivatives inspired by the natural product ceratamine A (**259**) was synthesized by Pan et al. [204]. The key step in the synthesis was the formation of the imidazoazepine core (scaffold **261**) via an intramolecular Heck reaction starting from **260**. The target compounds were tested for activity against the BGC-823, A549, HCT116, HepG2, and A2780 cancer cell lines. Derivatives **262**, **263**, and **264** displayed IC_50_ values of 20.0, 6.23, and 3.80 µM against the HCT116 cancer cell line, whereas the IC_50_ value of ceratamine A was 12.4 µM. Interestingly, compounds with substitutions possessing bulky groups, electron-withdrawing groups, or electron-donating groups on the benzyl substituent attached to the nitrogen atom of the azepine ring generally displayed decreased potency against the other four cell lines tested. Compound **265**, which contains a 3,4,5-trimethoxyphenyl substitution corresponding to the 3,5-dibromo-4-methoxyphenyl group of **259**, was active against all five cancer cell lines tested, with IC_50_ values ranging from 8.56–12.9 µM. The removal of the benzyloxymethyl group from the imidazole ring resulted in a dramatic loss of potency against the cell lines.

### 5.7. Purine Derivatives

**Figure 23:**
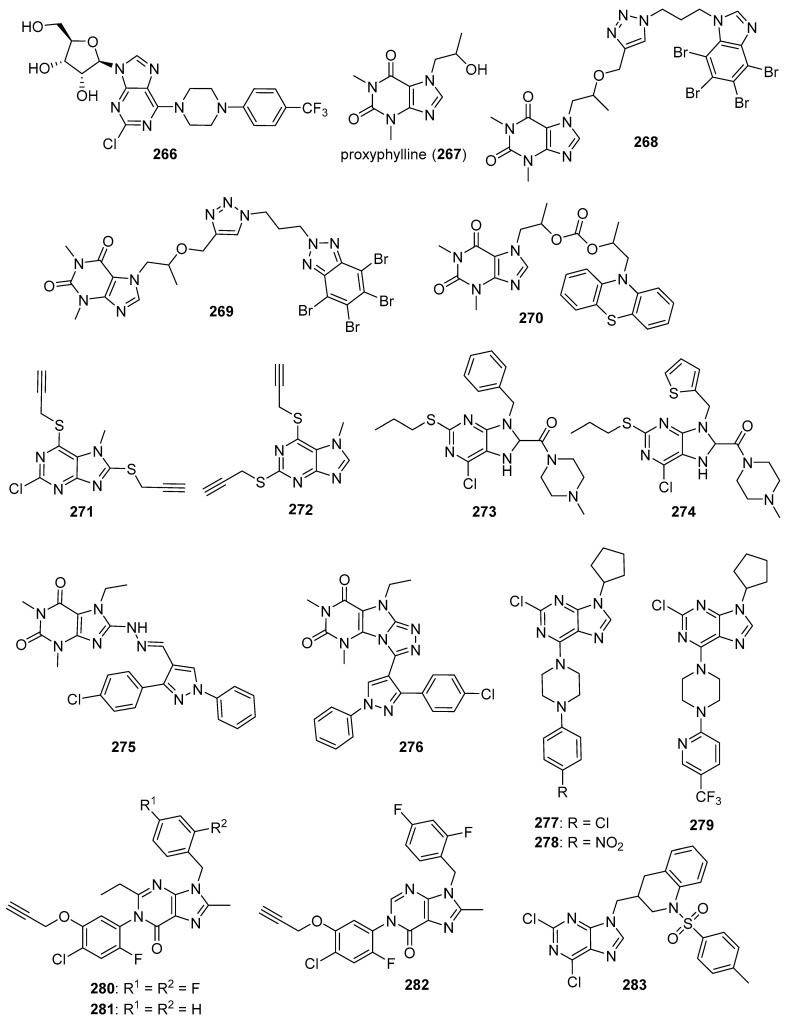
Purine derivatives with anticancer activity (compounds **266**–**283**).

Tuncbilek et al. synthesized β-d-ribofuranosyl-purine derivatives that were evaluated for anticancer activity against Huh7, HCT116, and MCF-7 cell lines [205]. Of these compounds, **266** (see Figure 23 for structures of compounds **266**–**283**) displayed the greatest activity, with IC_50_ values of 2, 1, and 4 μM against the three cell lines, respectively. By comparison, the standard drug 5-FU exhibited IC_50_ values of 30, 4, and 3 μM against the Huh7, HCT116, and MCF-7 cell lines. Compound **266** also displayed activity against HepG2, MAHLAVU, and FOCUS hepatocellular carcinoma cells (IC_50_ values of 3, 3, and 1 μM, respectively), while 5-FU exhibited IC_50_ values of 5, 10, and 4 μM against these three hepatocellular carcinoma cell lines, respectively.

Borowiecki et al. synthesized analogs of proxyphylline (**267**) and tested them for activity against CCRF-CEM and MCF-7 cancer cell lines [206]. Of the target compounds, polybrominated derivatives **268** and **269** displayed the greatest potency against CCRF-CEM cells, with EC_50_ values of 6.3 and 6.5 µM, respectively. Against MCF-7 cells, the greatest potency was observed for compounds **270** and **269**, which both exhibited EC_50_ values of 80 µM against this cancer cell line. By comparison, doxorubicin displayed an EC_50_ value of 1.4 µM against both CCRF-CEM and MCF-7 cells. These proxyphylline analogs were also tested for antifungal activity against *Candida albicans*, with compounds **270** and **269** exhibiting the best antifungal activity.

Kowalska et al. tested the anticancer activity of multisubstituted purine and xanthine derivatives against SNB-19, MDA-MB-231, and C32 cell lines and also counter-screened these compounds against normal HFF-1 cells [207]. The target compounds possessed a range of activities against the four cell lines tested. Compound **271** was highly active against SNB-19 cells, possessing an IC_50_ value of 0.07 μg/mL against this cell line with a selectivity index of 287 when compared to HFF-1 cells. Compound **272** displayed good potency against MDA-MB-231 cells, possessing an IC_50_ value of 1.00 μg/mL against this cell line with a selectivity index of 10 when compared to HFF-1 cells.

Zhao et al. synthesized 6-chloro-2-propylthio-8,9-dihydro-7H-purine derivatives as potential antiproliferative agents and evaluated their activity against A549, MGC-803, PC-3 and TE-1 cell lines [208]. Of the target compounds synthesized, **273** and **274** displayed the greatest activity against A549 cells, exhibiting IC_50_ values of 2.80 and 6.42 μM, respectively. Compound **274** displayed an IC_50_ value of 5.02 μM against PC-3 cells. For comparison, the standard drug 5-FU displayed IC_50_ values of 13.18 and 12.33 μM against A549 and PC-3 cells, respectively. When compound **273** was counter-screened against normal GES-1 cells, an IC_50_ value of 303 μM was obtained. Upregulation of E-cadherin (a biomarker of epithelial cells) and down-regulation of N-cadherin and vimentin (biomarkers of mesenchymal cells) was observed in A549 cells incubated with compound **273** at concentrations ranging from 0.5–2.0 µM. Dose-dependent inhibition of the migration of A549 cells was observed in a wound healing assay when compound **273** was incubated with these cells at concentrations ranging from 0.25–1.0 µM.

Shaaban et al. synthesized purines containing a pyrazole group as potential anticancer agents against A549, MCF-7, HepG2, Caco-2, and PC-3 cancer cells [209]. Counter-screening was performed against the HPBMC cell line. Compounds **275** and **276** displayed the lowest IC_50_ values (18.89 and 79.65 μM, respectively) against the A549 cell line (the IC_50_ of 5-FU was 83.03 μM); compound **275** exhibited a selectivity index of 32 and compound **276** displayed a selectivity index of 5.44 compared to HPBMC cells. For all other target compounds against the other cell lines, IC_50_ values were > 100 µM. In general, IC_50_ concentrations of compounds **275** and **276** increased the percentage of caspase 3/7 activation in the cancer cell lines to a greater degree than 5-FU.

Salas et al. synthesized a series of 31 purine derivatives as potential cytotoxic agents against CFPAC-1, NCI-H460, HL-60, Caco-2, HCT116, K562, MCF-7, MRC-5 cancer cell lines and normal MRC-5 cells [210]. The compounds showed little activity against CFPAC-1, Caco-2, and HCT116 cells. For the other four cell lines, the presence of a piperazine containing substituent at C6 was crucial for maintaining high activity, while a bulky substituent at C2 was detrimental. Compounds **277** and **278** displayed IC_50_ values ≤ 2.21 and ≤ 1.55 μM, respectively, against the NCI-H460, HL-60, K562, and MCF-7 cell lines, with **278** displaying a selectivity index of > 250 for HL-60 cells compared to MRC-5 cells. Compound **279** displayed the greatest potency (IC_50_ = 0.39 μM) against the K562 cell line. Compound **278** also induced apoptosis in HL-60 cells at concentrations of 5 µM and 50 µM.

Liu et al. synthesized purinone and imidazotriazinone derivatives as potential antitumor agents and tested these compounds against HepG2 and U-118 MG cancer cell lines [211]. Compound **280** displayed IC_50_ values of 2.0 and 3.8 μM, respectively, against these cancer cell lines. Compounds **281** and **282** exhibited IC_50_ values of 0.5 and 0.4 μM, respectively, against HepG2 cells. In terms of the general SAR trends, compounds possessing a purinone core were typically more active against the HepG2 cells compared to target compounds containing an imidazotriazinone core, and introduction of fluorine atoms on the benzyl substituent at N9 and the presence of a longer alkyl group at the C2 position of the purinone core conveyed increased anticancer activity.

Fernández-Sáez et al. synthesized purine derivatives substituted with various heterocyclic rings and evaluated their activity against MCF-7, A375, and HCT116 cancer cell lines [212]. Among these, 3-((2,6-dichloro-9*H*-purin-9-yl)methyl)-1-tosyl-1,2,3,4-tetrahydroquinoline (compound **283**) displayed the greatest potency. Compound **283** displayed EC_50_ values of 4.26, 5.54, and 2.80 μM against these cell lines, respectively, while the standard drug 5-FU exhibited EC_50_ values of 1.50 and 2.40 μM against MCF-7 and HCT116 cells. Compound **283** also displayed low cytotoxicity against RFP TERT normal fibroblasts (EC_50_ = 54.0 μM) and induced apoptosis in 58% of MCF-7 cancer cells when incubated with these cells at a concentration of 30 μM for 24 h, while apoptosis was observed in less than 10% of the vehicle control cells.

## 6. Conclusions

Cancer is one of the leading causes of mortality worldwide, placing a huge burden on the healthcare system and exacting a staggering human toll. Despite the many drugs available for treatment of various types of cancer, the side effects, resistance profiles, and variable efficacy of these drugs provides the motivation to discover new anticancer compounds. As illustrated in this review article, considerable attention has been devoted to the synthesis and anticancer evaluation of imidazole and fused imidazole derivatives in recent years. This review summarizes the in vitro and in vivo efficacy studies performed with these derivatives, mechanistic studies conducted on these candidates, and the SAR of the series presented. The work recapped here demonstrates that these imidazole-containing derivatives display anticancer activity through a wide range of mechanisms. While it is impossible to predict with certainty the candidates and approaches described in this review that will have the greatest impact on the treatment of cancer, the following future outlook identifies molecules and strategies that appear to be particularly promising to the authors. Although extensive work on antimicrotubule agents as anticancer compounds has been performed in the past, antitubulin candidates related to compound **8** show outstanding efficacy in murine cancer models [35,36], suggesting that compounds with this scaffold may have a future in cancer therapy. At the time of the writing of this review, a phase 3 trial is being planned to evaluate compound **8** for treating metastatic, castration-resistant prostate cancer (ClinicalTrials.gov Identifier: NCT04844749). Kinases are popular drug targets, but there have been selectivity issues due to their structural and sequence similarity. Considering the covalent binding of Nek2 inhibitors **85** and **87** to their putative target [89], such compounds may have enhanced duration of activity, may be effective at a lower dose, and could be less likely to engender drug resistance [213]. Given the effects of lead compound **125** on MCM2, a promising new target, **127** is considered an exciting, potent anticancer candidate considering its efficacy in vivo at doses of just 1 mg/kg [120] if off-target effects (including CYP450 inhibition and generation of ROS) can be minimized. Despite its challenges, polypharmacology is recognized as a promising strategy for cancer treatment due to the potential of multi-target agents to address the problem of drug resistance [214]. The dual HDAC1/2 and CDK2 inhibitor **141** displays in vivo anticancer efficacy [133], while the pharmacophore fusion strategy reported by He et al. indicated that compound **144** displays potent p53-MDM2/HDAC inhibitory activity and good activity in a murine xenograft model [138], making these molecules intriguing multi-target lead compounds. Compound **150** shows exceptionally tight binding to WDR5, strong inhibition of MLL1 histone methyltransferase activity, and excellent activity against MYC-driven cancer cell lines [144]. Targeting WDR5 may, thus, provide a strategy to exploit MYC, a critical driver of many cancers that has remained difficult to target directly due to its lack of defined small molecule binding sites [215]. Further investigation of the pharmacokinetic properties of compound **150** and its derivatives is, therefore, needed. Cutting edge drug discovery approaches, such as NMR-based fragment screening, also represent attractive strategies for the exploitation of WDR5, and, by extension, MYC, for cancer therapy [145]. While no HSP inhibitors have been approved to date, compound **191** is undergoing phase II clinical trials for the treatment of gastrointestinal cancer refractory to current treatments [216], indicating the promise of this agent for cancer treatment.

Our understanding of cancer biology continues to increase, but the development of effective, less toxic therapeutics remains a significant challenge, with drug resistance continuing to represent a major obstacle. Selective attack of key processes/enzymes of vital importance to specific cancers (targeted therapy) has already improved clinical outcomes in cancer [3]; the work summarized in this review shows that the highlighted compounds possessing imidazole/fused imidazole exhibit selectivity towards various targets and display potent activity cancer cell lines compared to normal cell lines. In some cases, minor modifications in a particular scaffold or the inclusion of imidazole via scaffold hopping can result in promising new anticancer activities. Many of the synthesized derivatives reported here display similar or greater potency compared to the reference drugs and promising activity in animal models of cancer, reinforcing both the versatility and utility of the imidazole moiety in modern medicinal chemistry. We hope that this review aids medicinal chemists and other pharmaceutical scientists in the discovery and development of new anticancer compounds with improved efficacy and safety.

## Data Availability

Not applicable.

## List of Abbreviations and Cell Lines

4T1: mouse mammary carcinoma cell line; 5-FU: 5-fluorouracil; 786-0: human renal cancer cell line; A2780: human ovarian cancer cell line; A375: human malignant melanoma cell line; A431: human epidermoid carcinoma cell line; A498: human renal cancer cell line; A549: human lung carcinoma cell line; ACHN: human renal cancer cell line; ACTH: adrenocorticotropic hormone; ADR: adriamycin; AGS: human stomach cancer cell line; ALK5: activin receptor-like kinase 5; ALP: alkaline phosphatase; ALT: alanine aminotransferase; APC/C: anaphase-promoting complex; AST: aspartate aminotransferase; ATM: ataxia telangiectasia mutated serine/threonine kinase; ATR: ataxia telangiectasia mutated and Rad3-related serine/threonine kinase; AUC: area under the curve; AURK: Aurora kinase; B16F10: murine melanoma cell line; BD: bromodomain; Beas-2B: human bronchial epithelial cell line; BEL-7402: human hepatocellular carcinoma cell line; BER: base excision repair; BET: bromodomain and extraterminal; BGC-823: human endocervical adenocarcinoma cell line; BHK: baby hamster kidney cell line; BJ: human normal lung fibroblast; BRCA: breast cancer gene; BSA: bovine serum albumin; BT474: human breast cancer cell line; BT-549: human breast cancer cell line; BTK: Bruton’s tyrosine kinase; BxPC-3: human pancreatic cancer cell line; C32: human melanoma cell line; C6: rat glioma cell line; CA 15-3: cancer antigen 15-3; Caco-2: human epithelial colorectal adenocarcinoma cell line; CAL27: human tongue squamous cell carcinoma cell line; Caov-3: human ovarian cancer cell line; Capan-1: human pancreatic adenocarcinoma cell line; CCRF-CEM/VBL: vinblastine-resistant leukemia cell line; CCRF-CEM: human acute T-lymphoblastic leukemia; CDK: cyclin-dependent kinase; CEA: carcinoembryonic antigen; CFPAC-1: human pancreatic adenocarcinoma cell line; Chk: checkpoint kinase; CHP-134: human neuroblastoma cell line; CML: chronic myelogenous leukemia; CNE-1: human nasopharyngeal carcinoma cell line; CT26: colorectal cancer cell line; ct-DNA: calf thymus deoxyribonucleic acid; CYP450: cytochrome P450; Daudi: p53 mutant Burkitt’s lymphoma cell line; DLD-1: human colorectal adenocarcinoma cell line; DMF: N, N-dimethyl formamide; DMSO: dimethyl sulfoxide; DSBs: double strand breaks; DU-145: human prostate cancer cell line; EC_50:_ half maximal effective concentration; EGFR: epidermal growth factor receptor; ELISA: enzyme-linked immunoassay; ER: estrogen receptor; EtBr: ethidium bromide; FAK: focal adhesion kinase; FGFR: fibroblast growth factor receptor; FOCUS: human hepatocellular carcinoma cell line; G4s: G-quadruplexes; GES-1: normal human gastric epithelial cell line; GI_50_: growth inhibitory 50%; GPCR: G-protein coupled receptor; GST: glutathione S-transferase; H1975: human non-small cell lung cancer cell line; H3255: human lung cancer cell line; H460: human lung cancer cell line; HaCaT: immortalized human keratinocyte cell line; HBL-100: human normal breast cell line; HCC: hepatocellular carcinoma; HCC827: human lung adenocarcinoma cell line; HCT: human colon carcinoma cell line; HCT116: human colorectal carcinoma cell line; HCT-15: human colorectal adenocarcinoma cell line; HDACs: histone deacetylases; HDF: primary human dermal fibroblast; HEK-293: human embryonic kidney cell line; HEK-293T: human embryonic kidney cell line containing the SV40 T-antigen; HeLa: human cervical cancer cell line; Hep3B: human hepatoma cell line; Hep3B-SR: sorafenib resistant hepatocellular carcinoma cancer cell line; HepG2: human liver carcinoma cell line; HFF-1: normal human foreskin fibroblast cell line; HL-60: human promyelocytic leukemia cell line; HL-7702: human normal hepatic cell line; HO-1: heme oxygenase-1; HPBMC: human peripheral blood mononuclear cell line; HR: homologous recombination; HSP: heat shock protein; HT29: human colorectal adenocarcinoma cell line; Huh-7: human hepatoma cell line; Huh7-SR: sorafenib resistant hepatocellular carcinoma cancer cell line; HUVEC: human umbilical vein endothelial cell line; i.p: intraperitoneal; IC_50:_ half maximal inhibitory concentration; IDO: indoleamine-2,3-dioxygenase; IMR-32: human neuroblastoma cell line; JEKO-1: human mantle cell lymphoma line; Jurkat: human acute T-lymphoblastic leukemia cell line; K562: human chronic myeloid leukemia cell line; K_b_: binding constant; KB: human carcinoma; KB-3-1: human epidermoid carcinoma cell line; KB-C2: multidrug resistant human carcinoma cell line; K_d:_ dissociation constant; KDM1A or LSD1: lysine-specific demethylase 1; L929: normal mouse fibroblast cell line; LCK: lymphocyte-specific protein tyrosine kinase; LN-405: human glioblastoma cell line; LO2: human normal liver cell line; LOX: lipoxygenase; M14: human melanoma cell line; Mahlavu: human hepatocellular carcinoma cell line; MAPK: mitogen-activated protein kinase; MCF-10A: human mammary gland epithelial cell line; MCF-10F: human normal breast epithelial cell line; MCF-7: breast cancer cell line; MCM: minichromosomal maintenance protein; MDA-MB-231: human breast adenocarcinoma cancer cell line; MDA-MB-468: human breast adenocarcinoma cancer cell line; MDM2: murine double minute 2; MDR: multidrug resistance; MEK: mitogen-activated protein kinase/ERK kinase (MEK)/extracellular-signal-regulated kinase; MGC-803: gastric cancer cell line; MGM: mean-graph midpoint; MIAPaCa-2: human pancreatic cancer cell line; MKN-45: human gastric adenocarcinoma cell line; MNU: N-methyl-N-nitrosourea; MOLM-13: human acute myeloid leukemia cell line; MRC-5: human normal skin fibroblast; mTOR: mammalian target of rapamycin; MTT: 3-(4,5-dimethylthiazol-2-yl)-2,5-diphenyl tetrazolium bromide; MV4-11: acute myelogenous leukemia cell line; NCI: National Cancer Institute; NCI-H1299: human non-small cell lung carcinoma cell line; NCI-H1975: non-small cell lung carcinoma cell line; NCI-H446: human prostate cancer cell line; NCI-H522: human non-small cell lung cancer cell line; NRK-52E: normal rat kidney epithelial cell line; OVCAR-3: human ovarian cancer cell line; PANC-1: pancreatic cancer cell line; PARP: poly(ADP-ribose)polymerase; PC-3: human prostate cancer cell line; PC-3/TxR: taxane-resistant human PC-3 cell line; PC9: human lung cancer cell line; PCNA: proliferating cell nuclear antigen; P-gp: P-glycoprotein; PI3K: phosphatidylinositol-3-kinase; PLC5: human hepatoma cell line; PLK: Polo like kinase; qPCR: quantitative polymerase chain reaction; RAF: rapidly accelerated fibrosarcoma; Raji: p53 mutant Burkitt’s lymphoma cell line; Ramos: non-Hodgkin lymphoma cell line; RFP TERT: immortalized fibroblast cell line; rhIDO1: Recombinant human IDO1; ROS: Reactive oxygen species; RPMI7951: Human skin malignant melanoma cell line; RPMI8226: Human myeloma cell line; RTK: receptor tyrosine kinase; RT-PCR: reverse transcription polymerase chain reaction; SAR: structure-activity relationship; SF-295: human glioblastoma cell line; SFK: Src family kinase; SH-SY5Y: human neuroblastoma cell line; SK-BR-3: HER overexpressing human breast cancer cell line; SK-Mel-2: human malignant melanoma cell line; SKOV3: human ovarian cancer cell line; SMMC-7721: human hepatocarcinoma cell line; SNB-19: human glioblastoma cell line; SPC-A1: human lung adenocarcinoma cell line; SPR: surface plasmon resonance; SR: human leukemia cell line; STAT3: signal transducer and activator of transcription 3; SU-DHL-4: human follicular B-cell lymphoma cell line; SU-DHL-6: human follicular B-cell lymphoma cell line; SW48: human colorectal carcinoma cell line; SW480: Human colorectal adenocarcinoma cell line; SW620: metastatic colorectal adenocarcinoma cell line; T47D: human ductal breast epithelial tumor cell line; TDO: tryptophan 2,3-dioxygenase; TE-1: human squamous cell carcinoma line; TGF-β: transforming growth factor-β; TGI: tumor growth inhibition; hERG: human ether-a-go-go-related gene; THP-1: human leukemia cell line; TKI: tyrosine kinase inhibitor; TPSA; Total polar surface area; TopI: type 1 topoisomerases; TopII: type 2 topoisomerases; U.S. FDA: United States Food and Drug Administration; U-118 MG: human glioblastoma cell line; U2OS: human osteosarcoma cell line; U87: human glioblastoma cell line; U87MG: human glioblastoma cell line; VEGFR-2: vascular endothelial growth factor receptor 2; Vero: Normal African green monkey kidney cell line; W138: Human normal embryonic lung cell line; WDR5: WD repeat domain 5; WM164: human melanoma cell line; WS-1: human normal skin fibroblast cell line; YCC11: HDAC inhibitor sensitive gastric cancer cell line; YCC3/7: HDAC inhibitor resistant gastric cancer cell line; Δ*Tm*: melting temperature.
